# Indium-mediated allylation in carbohydrate synthesis: A short and efficient approach towards higher 2-acetamido-2-deoxy sugars

**DOI:** 10.3762/bjoc.10.231

**Published:** 2014-09-19

**Authors:** Christopher Albler, Ralph Hollaus, Hanspeter Kählig, Walther Schmid

**Affiliations:** 1 Department of Organic Chemistry, University of Vienna, Währingerstrasse 38, 1090 Vienna, Austria

**Keywords:** allylation, carbohydrates, epoxidation, indium, multivalent glycosystems, organocatalysis

## Abstract

Higher aminosugars are interesting targets in carbohydrate synthesis since these compounds play important roles in biological systems. However, their availability from natural sources is limited. Thus, in order to investigate their biological function, the development of facile and adaptable routes to this class of compounds is of fundamental importance. Our synthetic route towards these target molecules makes use of readily accessible pentoses and hexoses, which are subjected to indium-mediated two-carbon chain elongation. Subsequent ozonolysis and treatment with base yields α,β-unsaturated aldehydes, which are stereoselectively epoxidized using Jørgenson’s protocol. After Wittig chain elongation the obtained allylic epoxides were regio- and stereoselectively opened with trimethylsilyl azide under palladium catalysis. Finally, a suitable deprotection protocol, starting with acidic acetate cleavage and ozonolysis was established. Peracetylation of the products simplifies purification and subsequent azide reduction followed by final deacetylation using methanolic sodium methoxide furnishes the title compounds.

## Introduction

The indium-mediated allylation of carbonyl compounds has proven to be a valuable tool for carbon chain elongation [1–3] in order to access rare, biologically active carbohydrates [4–8]. Herein we report the extension of this method towards the field of higher aminosugars by additionally applying a stereoselective epoxidation–azide opening strategy. The resulting compounds, aminoheptoses and octoses, have been scarcely investigated yet. However, they comprise interesting synthetic targets. Aminoheptoses function as constituents of the cell wall lipopolysaccharides of certain bacteria [9] whereas their ^99m^Tc complexes find practical applications in medicinal chemistry as tracers for tumor detection, myocardial ischemia or infarct diagnostics [10]. The group of Perez et al has taken a particular interest in the chemistry of 2-aminoheptoses [11–14] which they prepared via an amino-nitrile synthesis [15]. This method, first described by Kuhn and Kirschenlohr [16] for the synthesis of aminohexoses, proved to be straightforward, albeit the reproducibility and yield usually suffered from the formation of multiple side products [17]. Another interesting approach for the synthesis of higher aminosugars, published by Kunz and Deloisy, consists of an aza-Cope rearrangement of *N*-galactosyl-*N*-homoallylamines [18]. Aminooctoses, on the other hand, are present in the aminoglycoside antibiotic apramycin [19], in the form of an aminooctodiose derivative. However, only few syntheses of this dipyranoid aminosugar [20–21] were reported so far. Thus, we were interested in developing a general route towards the synthesis of these higher aminosugars. Since the preparation of new potent aminoglycoside antibiotics remains an important topic in medicinal chemistry [22], our precursors used should be flexible in terms of stereochemical variety, thus potentially allowing for an evaluation of structure–activity relationships.

## Results and Discussion

**Scheme 1:**
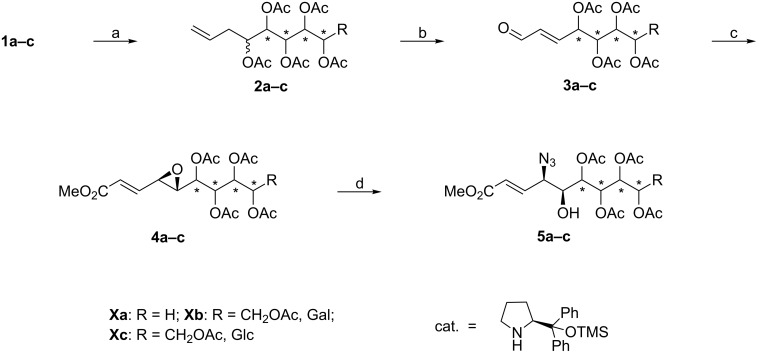
Functionalization of carbohydrates; reagents and conditions: (a) In, allyl bromide, EtOH/H_2_O, ultrasound, 2.5–7 h, then Ac_2_O/pyridine, DMAP, rt, 16 h, quant.; (b) O_3_, CH_2_Cl_2_, −78 °C then thiourea, rt, 16 h, then TEA, rt, 30–50 min, quant.; (c) H_2_O_2_, cat., CH_2_Cl_2_, −20 °C, 16 h, then Ph_3_P(CHCO_2_Me), CH_2_Cl_2_, rt, 1 h, 72–75%, 2 steps; (d) TMSN_3_, Pd(PPh_3_)_4_, THF, rt, 1 h, 76–85%.

We started our reaction sequence with an indium-mediated allylation of unprotected carbohydrates using D-arabinose (**1a**), D-galactose (**1b**) and D-glucose (**1c**) as starting materials. The Barbier-type chain elongation reaction furnished olefins **2a** and **2b** after acetylation in quantitative yield (Scheme 1). In the gluco-case a yield of 70% for **2c** was obtained owing to incomplete consumption of the starting material **1c**. Ozonolysis of the olefins **2** was performed using thiourea as reducing agent. Subsequently the generated 2-deoxyaldoses were treated with triethylamine (TEA) to yield α,β-unsaturated aldehydes **3a–c** quantitatively.

The aldehydes **3a–c** were stereoselectively epoxidized by applying the conditions developed by Jørgenson et al [23–25]. The required amine catalyst was synthesized starting from L-proline following a literature procedure [26]. In general the desired epoxyaldehydes were obtained with high diastereoselectivity at low temperatures (Table 1). The stereochemistry of compounds **3a–c** is in accordance with the proposed reaction mechanism [24] and was proven by applying various NMR methods on the final products (vide infra), which adopt rigid pyranoid conformations.

**Table 1:** Epoxidation of aldehydes **3a–c**.

entry	aldehyde	dr^a^	yield^b^ [%]

1	**3a**	9:1	64
2	**3b**	99:1	77
3	**3c**	9:1	61

^a^Determined either by comparison of integrals of representative ^1^H NMR signals of crude reaction products or after conventional column chromatographic separation of diastereomers; ^b^isolated yields of corresponding 2,3-epoxyaldehydes.

Unfortunately, we were not able to directly achieve the opening of the obtained 2,3-epoxyaldehydes with azide nucleophiles. A variety of different Lewis acids, solvent systems and azide sources were screened but all attempts led to the decomposition of these labile compounds. Conversion of the carbonyl moiety to an acetal group followed by treatment with azide nucleophiles also failed to yield any desired products. Therefore we decided to mask the aldehyde as an olefin. A Wittig reaction with methyl (triphenylphosphoranylidene)acetate (Ph_3_P(CHCO_2_Me)) generated allylic epoxides **4a–c**, which in turn permitted the application of reliable palladium chemistry for the epoxide opening [27–29]. Thus, compounds **4a–c** were regio- and stereoselectively opened with trimethylsilyl azide and Pd(PPh_3_)_4_ as a catalyst [30], furnishing *syn*-azido alcohols **5a–c**.

**Scheme 2:**
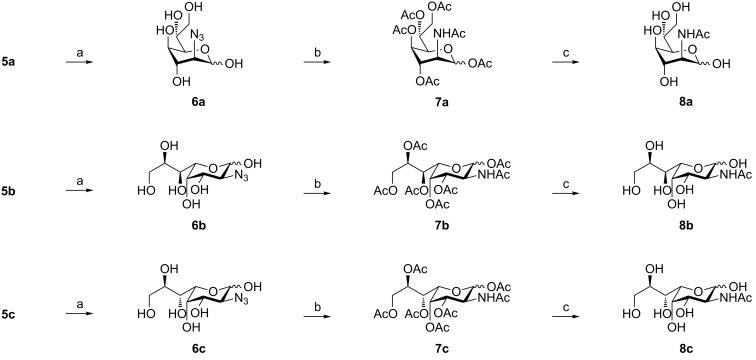
Deprotection sequence; reagents and conditions: (a): HCl/MeOH, rt, 16–24 h, then MeOH, CH_2_Cl_2_, O_3_, −78 °C then PPh_3_, rt, 16 h, 71–82%, 2 steps; (b): Ac_2_O/pyridine, DMAP, rt, 16 h then CH_3_CN, DTT, DIPA, rt, 2 h, then Ac_2_O/pyridine, DMAP, rt, 16 h, 59–66%, 3 steps; (c): NaOMe/MeOH, rt, 2–3 h, quant.

In the case of allylic azide **5a** we observed an acetate migration which resulted in a complex mixture of products bearing a free hydroxy moiety (mixture of free C5-OH/C6-OH/C7-OH = 2/1/1; Scheme 1). We reasoned that this migration was limited to the inherent *syn* relationship between C5-OH and C6-OAc in compound **5a**, since this behavior was not observed with compounds **5b** and **5c** which feature an *anti* alignment of the hydroxy groups. Next, we investigated the reduction of the azide moiety which should be followed by cleavage of the acetate protecting groups and ozonolysis in order to generate the target compounds. However, we found that compounds **5a–c** easily underwent 1,4-additions in the presence of nucleophiles or were decomposed under basic conditions. In this respect, DL-dithiothreitol/diisopropylamine (DTT/DIPA), DIBALH and NaBH_4_/MeOH failed to furnish the desired reduction products. Treatment with Pd/H_2_, PPh_3_, P(OMe)_3_ or SnCl_2_ on the other hand resulted in no or only very low conversion. Thus, we decided to change the reaction sequence. Consequently, we performed the acetate cleavage as the first step, since direct ozonolysis of compounds **5a** and **5c** again resulted in decomposition. Deacetylation in a methanolic HCl solution proved to be most efficient and the subsequent ozonolysis generated sugar azides **6a–c** (Scheme 2).

It turned out to be essential to perform the acidic acetate cleavage of compounds **5a–c** under strictly dry conditions, since even small amounts of water promoted an intramolecular Michael addition, leading to the formation of highly substituted tetrahydrofuran derivatives of the C-glycoside type [31].

Next, we investigated the azide reduction of compounds **6a–c** under different reaction conditions. Unfortunately DTT/DIPA, thioacetic acid, tributylphosphine/H_2_O and H_2_/Pd followed by *N*-acetylation with *N*-acetoxyphthalimide did not afford products **8a–c** in acceptable yields and/or purity. Since compounds **8a–c** could not be purified by conventional silica gel chromatography we decided to perform an additional acetylation step in order to avoid reversed-phase HPLC and to maintain our procedure as simple as possible. Hence, compounds **6a–c** were acetylated under standard conditions, subsequently reduced with DTT/DIPA and *N*-acetylated to yield compounds **7a–c** which could be easily purified. Final treatment with sodium methoxide in dry methanol then furnished the neat title compounds **8a–c**.

The configurations of compounds **6**, **7** and **8** were determined by applying various NMR methods. Since the stereochemistry at C-4 is given by the starting material, configurations of C-2 and C-3 can be easily deduced from the magnitude of the respective ^3^*J*_H,H_ coupling constants which reflect the torsion angles between these groups (Table 2).

**Table 2:** Characteristic coupling constants of compounds **7a–c**.

Compound	^3^*J*_1,2_ [Hz]	^3^*J*_2,3_ [Hz]	^3^*J*_3,4_ [Hz]	^3^*J*_4,5_ [Hz]

**7a**-α^a^	1.8	3.1	3.1	1.9
**7a**-β^a^	2.1	2.9	2.9	1.8
**7b**-α	3.7	11.5	3.3	0.9
**7b**-β	9.0	11.3	3.5	1.1
**7c**-α	3.7	11.6	3.3	1.0
**7c**-β	8.8	11.1	3.3	0.5

^a 4^C_1_-pyranoid form.

Therefore, the anticipated configuration of the final products could be verified. Since compounds **6a** and **8a** were found to exist as complex mixtures of conformers, only anomeric signals were assigned. This finding is in agreement with the fact that *ido*-configurated pyranose derivatives are known to adopt multiple conformations [32–34]. We were able to isolate and fully assign four distinct forms of the acetylated monosaccharide derivative **7a** (β-furanoid, ^4^C_1_ α/β-pyranoid and ^1^C_4_ α-pyranoid). Although **7a** is a known compound [15], the published NMR data are scarce. Herein, we provide the complete NMR data sets of 2-acetamido-1,3,4,6,7-penta-*O*-acetyl-2-deoxy-D-glycero-D-*ido*-heptose (**7a**). In the cases of compounds **6b**, **6c**, **7b**, **7c** and **8b** only α,β-pyranoid forms were detected (see copies of NMR spectra provided in Supporting Information File 1). However, NMR spectra of **8c** showed minor amounts of a compound which we considered to be its corresponding furanoid form. Unfortunately, we were not able to confirm this hypothesis owing to the very low concentration of this compound.

## Conclusion

In summary, we developed a simple, highly versatile route for the synthesis of rare, 2-amino-functionalized heptoses and octoses. The indium-mediated allylation strategy again revealed to be a useful tool for the preparation of two-carbon chain elongated carbohydrates. Two new stereocenters were formed with high diastereoselectivity in the course of the synthesis owing to the use of a chiral L-proline derived epoxidation catalyst. The introduction of nitrogen was achieved via a Tsuji–Trost-like azide opening of allylic epoxides. Although global deprotection proved to be cumbersome, we were able to develop a versatile reaction sequence to overcome this problem. The desired higher aminosugars were obtained in an overall yield of 21–29% over 7 steps. Additionally, these compounds should now be available in all stereochemical combinations by means of varying the starting materials, the epoxidation catalysts, or the mode of azide opening, respectively [35]. Furthermore, biologically active octodioses are now accessible from compounds **7b** and **7c**, which is subject of ongoing research.

## Supporting Information

File 1 Experimental section, spectral data and copies of ^1^H and ^13^C NMR spectra of compounds **2**–**8**.
