# Supplementary material for: Indium-mediated allylation in carbohydrate synthesis: A short and efficient approach towards higher 2-acetamido-2-deoxy sugars
Source: Beilstein J Org Chem. 2014 Sep 19;10:2230–4. doi: 10.3762/bjoc.10.231 (PMC4168925; doi:10.3762/bjoc.10.231)
Supplement: File 1 — Experimental section, spectral data and copies of 1H and 13C NMR spectra of compounds 2–8. [file Beilstein_J_Org_Chem-10-2230-s001.pdf]

Supporting Information

for

Indium-mediated allylation in carbohydrate synthesis: A short and efficient approach towards higher 2-acetamido-2-deoxy sugars

Christopher Albler, Ralph Hollaus, Hanspeter Kählig and Walther Schmid*

Address: Department of Organic Chemistry, University of Vienna, Währingerstrasse 38, 1090 Vienna, Austria

Email: Walther Schmid - walther.schmid@univie.ac.at

*Corresponding author

Experimental section, spectral data and copies of ^{1}H and ^{13}C NMR spectra of compounds 2–8

General methods

NMR spectra were recorded on a Bruker Avance DRX 400 or Bruker Avance III 600 spectrometer using $CDCl_3$, D_2O or CD_3OD for calibration. MS experiments were measured in the ESI mode on a Finnigan MAT 900 spectrometer. For chromatography Merck silica gel 60 (0.004–0.063 mm) was used. For TLC monitoring Merck plates (silica gel 60 F254) were used; plates were stained by treatment with a solution of anis aldehyde (1 ml) in AcOH (100 ml) and H_2SO_4 (2 ml), followed by charring with a heat gun. All solvents were distilled before use. Ozonolysis was performed using an Anseros Generator COM-AD-04. Optical

rotations were measured on a Perkin–Elmer Polarimeter 341. IR spectra were recorded on a Bruker Vertex 70 FT-IR spectrometer. Chemicals were purchased in reagent grade.

General Procedure for Allylation/Peracetylation of Aldoses. Synthesis of 2a-2c (Method A): To a solution of the monosaccharide in the corresponding solvent indium powder and allyl bromide were added and the resulting suspension subjected to sonication for 2.5-7 h. When TLC (acetone/2-propanol/H_2O = 5/4/1) indicated complete conversion of the starting material, the cloudy white to light blue reaction mixture was neutralized with 1 M NaOH, if necessary, and evaporated to dryness. The residue was redissolved in a mixture of pyridine (Pyr) and acetic anhydride (Ac_2O) = 1/1 under argon and a catalytic amount of 4-(dimethylamino)pyridine (DMAP) was added. The resulting reaction mixture was stirred at room temperature for 16 h and then poured into cooled (0°C) 3 M HCl. The aqueous phase was extracted three times with dichloromethane (DCM) and the combined organic layers were dried over anhydrous $MgSO_4$ and concentrated under reduced pressure. Purification by silica gel chromatography afforded olefins **2a-c** as clear viscous oils.

General Procedure for Ozonolysis/Elimination. Synthesis of 3a-3c (Method B): A stirred solution of the olefin in dry DCM was cooled to -78°C and ozone was bubbled through the reaction mixture until a blue color persisted, whereupon air was bubbled through the solution until the blue color vanished. Thiourea was then added and the reaction mixture was allowed to warm to room temperature and stirred for 16 h. The white precipitate formed was filtered off, TEA was added and the resulting light yellow solution stirred for 30-50 min at room temperature as judged by TLC (hexane/ethyl acetate [HE/EA] = 1/1). The reaction mixture was then poured into cooled (0°C) 1 M HCl. The aqueous phase was extracted three times with DCM and the combined organic layers were dried over anhydrous $MgSO_4$ and

evaporated to dryness. Compounds **3a-c** were obtained as white crystalline solids and used in the next step without further purification.

General Procedure for Epoxidation/Wittig Reaction. Synthesis of 4a-4c (Method C): A stirred solution of the unsaturated aldehyde in dry DCM was cooled to -20°C via a cryostat and (*S*)-(−)-alpha,alpha-diphenyl(pyrrolidin-2-yl)methanol trimethylsilyl ether was added followed by H_2O_2 (50%). The reaction mixture was stirred at -20°C for 16 h after which water was added and the mixture stirred vigorously at room temperature for additional 10 min. The aqueous phase was extracted three times with DCM and the combined organic layers were dried over anhydrous $MgSO_4$ and evaporated to dryness. The crude reaction product was redissolved in dry DCM and $Ph_3P(CHCO_2Me)$ was added. The resulting solution was stirred at room temperature for 1 h and then concentrated under reduced pressure. Purification by silica gel chromatography afforded epoxides **4a-c** as white to light yellow crystalline solids.

General Procedure for Epoxide Opening. Synthesis of 5a-5c (Method D): Dry tetrahydrofuran (THF) was degassed using the freeze-pump-thaw technique. Then the epoxide was added under argon followed by trimethylsilyl azide ($TMSN_3$) and tetrakis(triphenylphosphine)palladium(0) ($Pd(PPh_3)_4$). The bright yellow reaction mixture was stirred at room temperature for 1 h, then quenched with a solution of citric acid in methanol and stirred for an additional hour at room temperature. The reaction mixture was concentrated under reduced pressure and purification by silica gel chromatography afforded azides **5a-c** as colorless to light yellow viscous oils.

General Procedure for Deacetylation/Ozonolysis. Synthesis of 6a-6c (Method E): Acetyl chloride (AcCl) was added to dry methanol (MeOH) under argon and stirred at room temperature for 15 min. The resulting methanolic HCl solution was added to a solution of the

azide in dry MeOH under argon and stirred at room temperature for 16-24 h as judged by TLC (DCM/MeOH = 6/1). To avoid intramolecular 1,4-addition, powdered MS 4Å was then added and the reaction mixture was stirred vigorously for 20 minutes. The molecular sieve was filtered off and the filtrate diluted with dry MeOH and a few ml of dry DCM. The resulting solution was cooled to -78°C and ozone was bubbled through the reaction mixture until a blue color persisted, whereupon air was bubbled through the solution until the blue color vanished. Triphenylphosphine (PPh_3) was then added and the reaction mixture was allowed to warm to room temperature and stirred for 16 h. The solution was concentrated under reduced pressure and purification by silica gel chromatography afforded sugar azides **6a-c** as light yellow viscous oils.

General Procedure for Azide Reduction. Synthesis of 7a-7c (Method F): The sugar azide was dissolved in Pyr/Ac_2O = 1/1 under argon and a catalytic amount of DMAP was added. The resulting reaction mixture was stirred at room temperature for 16 h and then evaporated to dryness. The crude reaction product was redissolved in dry acetonitrile (MeCN) under argon and DTT followed by DIPA were added. The resulting solution was stirred at room temperature for 2 h and then evaporated to dryness. The residue was redissolved in Pyr/Ac_2O = 1/1 under argon and a catalytic amount of DMAP was added. The resulting reaction mixture was stirred at room temperature for 16 h and then evaporated to dryness. Purification by silica gel chromatography afforded peracetylated aminosugars **7a-c** as colorless to light yellow viscous oils.

General Procedure for Zemplén Saponification. Synthesis of 8a-8c (Method G): Similar as described in [1]. To a solution of the peracetylated aminosugar in dry MeOH a catalytic amount of sodium methoxide (NaOMe) was added under argon and stirred at room temperature for 2-3 h as judged by TLC (acetone/2-propanol/H_2O = 5/4/1). Then a small

amount of acidic ion exchange resin was added and the reaction mixture stirred for additional 10 min at room temperature. After filtration, the solution was evaporated to dryness and the residue was redissolved in water, washed three times with ethyl acetate (EA) and evaporated to dryness. The aminosugars **8a-c** obtained needed no further purification.

1,2,3,4,5-Penta-*O*-acetyl-6,7,8-trideoxy-L-gulo-7-octenitol (2a): D-arabinose (250 mg, 1.67 mmol) in ethanol/H_2O = 4/1 (40 ml) was treated according to method A with indium (382 mg, 3.33 mmol) and allyl bromide (504 µl, 5.83 mmol). Purification by silica gel chromatography was performed using HE/EA = 2/1 as eluent; yield: 670 mg, (100%) as a mixture of diastereomers (dr = 9/1). ^{1}H NMR ($CDCl_3$, 400 MHz, 25°C) (major diastereomer): δ = 2.05, 2.05, 2.06, 2.06, 2.12 (5s, 15 H, 5 OAc), 2.34 (m, 2 H, 6a-H, 6b-H), 4.12 (dd, $^3J_{1a,2}$ = 5.4 Hz, $^2J_{1a,1b}$ = 12.4 Hz, 1 H, 1a-H), 4.24 (dd, $^3J_{1b,2}$ = 3.4 Hz, $^2J_{1a,1b}$ = 12.4 Hz, 1 H, 1b-H), 5.08 (m, 4 H, 2-H, 5-H, 8a-H, 8b-H), 5.30 (dd, $^3J_{3,4}$ = 4.0 Hz, $^3J_{4,5}$ = 6.6 Hz, 1 H, 4-H), 5.42 (dd, $^3J_{3,4}$ = 4.0 Hz, $^3J_{2,3}$ = 6.9 Hz, 1 H, 3-H), 5.73 (m, 1 H, 7-H); ^{13}C NMR ($CDCl_3$, 100 MHz, 25°C): δ = 21.0, 21.1, 21.1, 21.2, 21.2 (5 OAc), 35.6 (6-C), 61.9 (1-C), 68.7 (4-C), 68.9 (2-C), 70.4 (3-C), 70.7 (5-C), 119.4 (8-C), 132.4 (7-C), 170.2, 170.2, 170.3, 170.6, 170.9 (5 CO-OAc); HRMS (ESI) *m/z* $[M+Na]^+$ calculated 425.1424, found 425.1420.

1,2,3,4,5,6-Hexa-*O*-acetyl-7,8,9-trideoxy-L-galacto-D-glycero-8-octenitol (2b): D-galactose (280 mg, 1.55 mmol) in ethanol/0,1 M HCl = 4/1 (40 ml) was treated according to method A with indium (357 mg, 3.11 mmol) and allyl bromide (470 µl, 5.44 mmol). Purification by silica gel chromatography was performed using HE/EA = 1/1 as eluent; yield: 737 mg (100%) as a mixture of diastereomers (dr = 7/1). ^{1}H NMR ($CDCl_3$, 400 MHz, 25°C) (major diastereomer): δ = 2.00, 2.01, 2.06, 2.08, 2.10, 2.12 (6s, 18 H, 6 OAc), 2.41 (m, 2 H, 7a-H, 7b-H), 3.83 (dd, $^3J_{1a,2}$ = 7.1 Hz, $^2J_{1a,1b}$ = 11.6 Hz, 1 H, 1a-H), 4.28 (dd, $^3J_{1b,2}$ = 4.6 Hz, $^2J_{1a,1b}$ = 11.6 Hz, 1 H, 1b-H), 4.94 (ddd, $^3J_{6,7}$ = 4.2 Hz, $^3J_{5,6}$ = 7.9 Hz, 1 H, 6-H), 5.13 (m, 2 H, 9a-H, 9b-H), 5.20 (dd, $^3J_{4,5}$ = 2.1 Hz, $^3J_{5,6}$ = 7.9 Hz, 1 H, 5-H), 5.23 (m, 2 H, 3-H, 2-H), 5.36 (dd,

$^3J_{4,5}$ = 2.1 Hz, $^3J_{3,4}$ = 9.4 Hz, 1 H, 4-H), 5.72 (m, 1 H, 8-H); ^{13}C-NMR ($CDCl_3$, 100 MHz, 25 °C): δ = 21.0, 21.0, 21.0, 21.1, 21.1, 21.2 (6 OAc), 35.5 (7-C), 62.6 (1-C), 67.7 (4-C), 68.1 (3-C), 68.3 (2-C), 70.2 (5-C), 71.1 (6-C), 119.2 (9-C), 132.5 (8-C), 170.2, 170.3, 170.3, 170.4, 170.6, 170.8 (6 CO-OAc); HRMS (ESI) *m/z* $[M+Na]^+$ calculated 497.1635, found 497.1623.

1,2,3,4,5,6-Hexa-*O*-acetyl-7,8,9-trideoxy-L-gulo-D-glycero-8-octenitol (2c): D-glucose hydrate (298 mg, 1.50 mmol) in ethanol/0,1 M HCl = 4/1 (40 ml) was treated according to method A with indium (344 mg, 3.00 mmol) and allyl bromide (454 µl, 5.25 mmol). Purification by silica gel chromatography was performed using HE/EA = 3/1 as eluent; yield: 500 mg (70%) as a mixture of diastereomers (dr = 5/1). ^{1}H NMR ($CDCl_3$, 400 MHz, 25°C) (major diastereomer): δ = 2.05, 2.06, 2.08, 2.09, 2.12, 2.14 (6s, 18 H, 6 OAc), 2.28 (m, 2 H, 7a-H, 7b-H), 4.14 (dd, $^3J_{1a,2}$ = 5.0 Hz, $^2J_{1a,1b}$ = 12.6 Hz, 1 H, 1a-H), 4.24 (dd, $^3J_{1b,2}$ = 2.6 Hz, $^2J_{1a,1b}$ = 12.6 Hz, 1 H, 1b-H), 4.98 (ddd, $^3J_{2,3}$ = 8.3 Hz, 1 H, 2-H), 5.11 (m, 3 H, 5-H, 9a-H, 9b-H), 5.32 (m, 2 H, 4-H, 6-H), 5.43 (dd, $^3J_{3,4}$ = 2.5 Hz, $^3J_{2,3}$ = 8.3 Hz, 1 H, 3-H), 5.69 (m, 1 H, 8-H); ^{13}C-NMR ($CDCl_3$, 100 MHz, 25 °C): δ = 20.5, 20.6, 20.7, 20.7, 20.8, 20.8 (6 OAc), 34.4 (7-C), 61.4 (1-C), 68.4 (4-C), 68.9 (3-C), 69.2 (2-C), 69.9 (6-C), 70.9 (5-C), 118.6 (9-C), 132.6 (8-C), 169.7, 169.8, 170.0, 170.2, 170.4, 170.6 (6 CO-OAc); ESI-MS: *m/z* 497.16 $[M+Na]^+$, 513.14 $[M+K]^+$.

(2*E*)-4,5,6,7-Tetra-*O*-acetyl-2,3-dideoxy-D-arabino-hept-2-enose (3a): Olefin **2a** (1340 mg, 3.33 mmol) was treated with ozone in 50 ml of dry DCM according to method B and quenched with thiourea (330 mg, 4.34 mmol). For the elimination TEA (1850 µl, 13.35 mmol) was used; yield: 1146 mg, (100%). $[\alpha]^D_{20}$ = +49.5° (11.0, CH_2Cl_2); m.p. 75-77°C; ^{1}H NMR ($CDCl_3$, 400 MHz, 25°C): δ = 2.05, 2.06, 2.08, 2.15 (4s, 12 H, 4 OAc), 4.18 (dd, $^3J_{6,7a}$ = 4.7 Hz, $^2J_{7a,7b}$ = 12.3 Hz, 1 H, 7a-H), 4.26 (dd, $^3J_{6,7b}$ = 2.2 Hz, $^2J_{7a,7b}$ = 12.3 Hz, 1 H, 7b-H),

5.23 (ddd, $^3J_{6,7b}$ = 2.2 Hz, $^3J_{6,7a}$ = 4.7 Hz, $^3J_{5,6}$ = 9.1 Hz, 1 H, 6-H), 4.45 (dd, $^3J_{4,5}$ = 2.5 Hz, $^3J_{5,6}$ = 9.1 Hz, 1 H, 5-H), 5.82 (ddd, $^4J_{2,4}$ = 2.2 Hz, $^3J_{4,5}$ = 2.5 Hz, $^3J_{3,4}$ = 3.9 Hz, 1 H, 4-H), 6.17 (ddd, $^4J_{2,4}$ = 2.2 Hz, $^3J_{1,2}$ = 7.7 Hz, $^3J_{2,3}$ = 15.8 Hz, 1 H, 2-H), 6.68 (dd, $^3J_{3,4}$ = 3.9 Hz, $^3J_{2,3}$ = 15.8 Hz, 1 H, 3-H), 9.54 (d, $^3J_{1,2}$ = 7.7 Hz, 1 H, 1-H); ^{13}C NMR ($CDCl_3$, 100 MHz, 25°C): δ = 20.9, 20.9, 21.0, 21.1 (4 OAc), 62.1 (7-C), 68.5 (6-C), 69.6 (5-C), 70.1 (4-C), 133.3 (2-C), 149.6 (3-C), 169.9, 170.0, 170.1, 170.9 (4 CO-OAc), 192.6 (1-C); HRMS (ESI) *m/z* $[M+Na]^+$ calculated 367.1005, found 367.1006.

(2*E*)-4,5,6,7,8-Penta-*O*-acetyl-2,3-dideoxy-D-galacto-oct-2-enose (3b): Olefin **2b** (1258 mg, 2.65 mmol) was treated with ozone in 50 ml of dry DCM according to method B and quenched with thiourea (277 mg, 3.64 mmol). For the elimination TEA (1470 µl, 10.60 mmol) was used; yield: 1103 mg, (100%). $[\alpha]^D{}_{20}$ = -1.4° (7.6, CH_2Cl_2); m.p. 154-156°C; ^{1}H NMR ($CDCl_3$, 100 MHz, 25°C): δ = 2.02, 2.03, 2.08, 2.12, 2.16 (5s, 15 H, 5 OAc), 3.86 (dd, $^3J_{7,8a}$= 7.6 Hz, $^3J_{8a,8b}$= 11.6 Hz, 1 H, 8a-H), 4.28 (dd, $^3J_{7,8b}$= 5.0 Hz, $^3J_{8a,8b}$= 11.6 Hz, 1 H, 8b-H), 5.35 (ddd, $^3J_{6,7}$= 1.9 Hz, $^3J_{7,8b}$= 5.0 Hz, $^3J_{7,8a}$= 7.6 Hz, 1 H, 7-H), 5.38 (dd, $^3J_{4,5}$= 2.0 Hz, $^3J_{5,6}$ = 10.1 Hz, 1 H, 5-H), 5.45 (dd, $^3J_{6,7}$= 1.9 Hz, $^3J_{5,6}$ = 10.1 Hz, 1 H, 6-H), 5.70 (ddd, $^4J_{2,4}$= 1.8 Hz, $^3J_{4,5}$= 2.0 Hz, $^3J_{3,4}$= 4.0 Hz, 1 H, 4-H), 6.13 (ddd, $^4J_{2,4}$= 1.8 Hz, $^3J_{1,2}$= 7.7 Hz, $^3J_{2,3}$= 15.7 Hz, 1 H, 2-H), 6.65 (dd, $^3J_{3,4}$= 4.0 Hz, $^3J_{2,3}$= 15.7 Hz, 1 H, 3-H), 9.52 (d, $^3J_{1,2}$= 7.7 Hz, 1 H, 1-H); ^{13}C-NMR ($CDCl_3$, 100 MHz, 25 °C): δ = 20.5, 20.5, 20.5, 20.6, 20.6 (5 OAc), 62.4 (8-C), 67.6 (6-C), 67.6 (7-C), 68.3 (5-C), 69.4 (4-C), 133.2 (2-C), 149.7 (3-C), 170.0, 170.1, 170.2, 170.5, 170.8 (5 CO-OAc), 192.5 (1-C); HRMS (ESI) *m/z* $[M+Na]^+$ calculated 439.1216, found 439.1220.

(2*E*)-4,5,6,7,8-Penta-*O*-acetyl-2,3-dideoxy-D-gluco-oct-2-enose (3c): Olefin **2c** (917 mg, 1.93 mmol) was treated with ozone in 40 ml of dry DCM according to method B and quenched with thiourea (191 mg, 2.51 mmol). For the elimination TEA (1070 µl, 7.72 mmol)

was used; yield: 804 mg (100%). $[\alpha]^{D}{}_{20}$ = +9.4° (5.9, CH_2Cl_2); m.p. 83-85°C; ^{1}H NMR ($CDCl_3$, 400 MHz, 25°C): δ = 2.06, 2.06, 2.09, 2.11, 2.15 (5s, 15 H, 5 OAc), 4.11 (dd, $^3J_{7,8a}$ = 5.3 Hz, $^2J_{8a,8b}$ = 12.4 Hz, 1 H, 8a-H), 4.27 (dd, $^3J_{7,8b}$ = 3.1 Hz, $^2J_{8a,8b}$ = 12.4 Hz, 1 H, 8b-H), 5.07 (ddd, $^3J_{7,8b}$ = 3.1 Hz, $^3J_{7,8a}$ = 5.3 Hz, $^3J_{6,7}$ = 7.3 Hz, 1 H, 7-H), 5.38 (dd, $^3J_{5,6}$ = 3.84 Hz, $^3J_{4,5}$ = 6.3 Hz, 1 H, 5-H), 5.43 (dd, $^3J_{5,6}$ = 3.84 Hz, $^3J_{6,7}$ = 7.3 Hz, 1 H, 6-H), 5.65 (ddd, $^4J_{2,4}$ = 1.6 Hz, $^3J_{3,4}$ = 4.6 Hz, $^3J_{4,5}$ = 6.3 Hz, 1 H, 4-H), 6.21 (ddd, $^4J_{2,4}$ = 1.6 Hz, $^3J_{1,2}$ = 7.6 Hz, $^3J_{2,3}$ = 15.8 Hz, 1 H, 2-H), 6.76 (dd, 1 H, $^3J_{3,4}$ = 4.6 Hz, $^3J_{2,3}$ = 15.8 Hz, 3-H), 9.59 (d, $^3J_{1,2}$ = 7.6 Hz, 1 H, 1-H); ^{13}C-NMR ($CDCl_3$, 100 MHz, 25 °C): δ = 20.9, 21.0, 21.1, 21.1, 21.1 (5 OAc), 61.9 (8-C), 68.8 (6-C), 68.9 (7-C), 70.1 (5-C), 70.7 (4-C), 133.8 (2-C), 148.4 (3-C), 169.7, 169.9, 170.0, 170.2, 170.9 (5 CO-OAc), 192.6 (1-C); HRMS (ESI) *m/z* $[M+Na]^+$ calculated 439.1216, found 439.1212.

Methyl (2*E*)-6,7,8,9-Tetra-*O*-acetyl-2,3-dideoxy-4*R*,5*S*-epoxy-D-arabino-nona-2-enonate (4a): Aldehyde **3a** (500 mg, 1.45 mmol) was epoxidized with the catalyst (71 mg, 0.22 mmol) and H_2O_2 50% (108 µl, 1.88 mmol) in 25 ml of dry DCM according to method C. For the Wittig reaction $Ph_3P(CHCO_2Me)$ (970 mg, 2.90 mmol) was used. Purification by silica gel chromatography was performed using HE/EA = 3/1 as eluent; yield: 435 mg, (72%). $[\alpha]^{D}{}_{20}$ = +20.7° (4.5, CH_2Cl_2); m.p. 82-84°C; ^{1}H NMR ($CDCl_3$, 400 MHz, 25°C): δ = 2.06, 2.06, 2.12, 2.13 (4s, 12 H, 4 OAc), 3.02 (dd, $^3J_{4,5}$ = 2.0 Hz, $^3J_{5,6}$ = 4.8 Hz, 1 H, 5-H), 3.35 (ddd, $^4J_{2,4}$ = 0.7 Hz, $^3J_{4,5}$ = 2.0 Hz, $^3J_{3,4}$ = 6.7 Hz, 1 H, 4-H), 3.75 (s, 3 H, OMe), 4.18 (dd, $^3J_{8,9a}$ = 4.6 Hz, $^2J_{9a,9b}$ = 12.5 Hz, 1 H, 9a-H), 4.26 (dd, $^3J_{8,9b}$ = 2.9 Hz, $^2J_{9a,9b}$ = 12.5 Hz, 1 H, 9b-H), 5.15 (dd, $^3J_{5,6}$ = 2.7 Hz, $^3J_{6,7}$ = 4.8 Hz, 1 H, 6-H), 5.16 (ddd, $^3J_{8,9b}$ = 2.9 Hz, $^3J_{8,9a}$ = 4.6 Hz, $^3J_{7,8}$ = 8.7 Hz, 1 H, 8-H), 5.48 (dd, $^3J_{6,7}$ = 4.8 Hz, $^3J_{7,8}$ = 8.7 Hz, 1 H, 7-H), 6.15 (dd, $^4J_{2,4}$ = 0.6 Hz, $^3J_{2,3}$ = 15.7 Hz, 1 H, 2-H), 6.62 (dd, $^3J_{3,4}$ = 6.7 Hz, $^3J_{2,3}$ = 15.7 Hz, 1 H, 3-H); ^{13}C NMR ($CDCl_3$, 100 MHz, 25°C): δ = 21.0, 21.0, 21.1, 21.2 (4 OAc), 52.2 (OMe), 53.7 (4-C), 59.8 (5-C), 61.9 (9-C), 68.4 (8-C), 69.5 (6-C), 69.8 (7-C), 125.0 (2-C), 142.8 (3-C), 166.1 (1-

C), 170.1, 170.2, 170.3, 170.9 (4 CO-OAc); HRMS (ESI) *m/z* $[M+Na]^+$ calculated 439.1216, found 439.1218.

Methyl (2*E*)-6,7,8,9,10-Penta-*O*-acetyl-2,3-dideoxy-4*R*,5*S*-epoxy-D-galacto-deca-2-enonate (4b): Aldehyde **3b** (500 mg, 1.20 mmol) was epoxidized with the catalyst (59 mg, 0.18 mmol) and H_2O_2 50% (90 µl, 1.57 mmol) in 25 ml of dry DCM according to method C. For the Wittig reaction $Ph_3P(CHCO_2Me)$ (802 mg, 2.40 mmol) was used. Purification by silica gel chromatography was performed using HE/EA= 2/1 as eluent; yield: 437 mg, (75%). $[\alpha]^D_{20}$ = +31.8° (4.5, CH_2Cl_2); m.p. 182-184°C; ^{1}H NMR ($CDCl_3$, 400 MHz, 25°C): δ = 2.02, 2.08, 2.08, 2.08, 2.10 (5s, 15 H, 5 OAc), 2.94 (dd, $^3J_{4,5}$ = 1.9 Hz, $^3J_{5,6}$ = 4.3 Hz, 1 H, 5-H), 3.44 (dd, $^3J_{4,5}$ = 1.9 Hz, $^3J_{3,4}$ = 7.3 Hz, 1 H, 4-H), 3.73 (s, 3 H, OMe), 3.85 (dd, $^3J_{9,10a}$ = 7.5 Hz, $^2J_{10a,10b}$ = 11.6 Hz, 1 H, 10a-H), 4.27 (dd, $^3J_{9,10b}$ = 4.9 Hz, $^2J_{10a,10b}$ = 11.6 Hz, 1 H, 10b-H), 5.10 (dd, $^3J_{6,7}$ = 1.8 Hz, $^3J_{5,6}$ = 4.3 Hz, 1 H, 6-H), 5.33 (ddd, $^3J_{8,9}$ = 2.0 Hz, $^3J_{9,10b}$ = 4.9 Hz, $^3J_{9,10a}$ = 7.5 Hz, 1 H, 9-H), 5.37 (dd, $^3J_{8,9}$ = 2.0 Hz, $^3J_{7,8}$ = 10.0 Hz, 1 H, 8-H), 5.43 (dd, $^3J_{6,7}$ = 1.8 Hz, $^3J_{7,8}$ = 10.0 Hz, 1 H, 7-H), 6.13 (d, $^3J_{2,3}$ = 15.7 Hz, 1 H, 2-H), 6.55 (dd, $^3J_{3,4}$ = 7.3 Hz, $^3J_{2,3}$ = 15.7 Hz, 1 H, 3-H); ^{13}C-NMR ($CDCl_3$, 100 MHz, 25 °C): δ = 20.9, 20.9, 21.0, 21.0, 21.1 (5 OAc), 52.2 (OMe), 54.0 (4-C), 58.0 (5-C), 62.5 (10-C), 67.4 (7-C), 67.5 (8-C), 67.5 (9-C), 67.5 (6-C), 125.0 (2-C), 143.3 (3-C), 166.0 (1-C), 169.9, 170.1, 170.2, 170.7, 170.8 (5 CO-OAc); HRMS (ESI) *m/z* $[M+Na]^+$ calculated 511.1428, found 511.1439.

Methyl (2*E*)-6,7,8,9,10-Penta-*O*-acetyl-2,3-dideoxy-4*R*,5*S*-epoxy-D-gluco-deca-2-enonate (4c): Aldehyde **3c** (350 mg, 0.84 mmol) was epoxidized with the catalyst (41 mg, 0.13 mmol) and H_2O_2 50% (63 µl, 1.10 mmol) in 20 ml of dry DCM according to method C. For the Wittig reaction $Ph_3P(CHCO_2Me)$ (562 mg, 1.68 mmol) was used. Purification by silica gel chromatography was performed using HE/EA = 2/1 as eluent; yield 296 mg (72%). $[\alpha]^D_{20}$ = +34.3° (2.7, CH_2Cl_2); m.p. 112-114°C; ^{1}H NMR ($CDCl_3$, 400 MHz, 25°C): δ = 2.04, 2.07,

2.09, 2.10, 2.12 (5s, 15 H, 5 OAc), 2.98 (dd, $^3J_{5,6}$ = 1.8 Hz, $^3J_{4,5}$ = 5.4 Hz, 1 H, 5-H), 3.52 (dd, $^3J_{4,5}$ = 5.4 Hz, $^3J_{3,4}$ = 7.2 Hz, 1 H, 4-H), 3.74 (s, 3 H, OMe), 4.11 (dd, $^3J_{9,10a}$ = 5.8 Hz, $^2J_{10a,10b}$ = 12.3 Hz, 1 H, 10a-H), 4.30 (dd, $^3J_{9,10b}$ = 4.0 Hz, $^2J_{10a,10b}$ = 12.3 Hz, 1 H, 10b-H), 5.01 (dd, $^3J_{5,6}$ = 1.8 Hz, $^3J_{6,7}$ = 4.8 Hz, 1 H, 6-H), 5.04 (ddd, $^3J_{9,10b}$ = 4.0 Hz, $^3J_{8,9}$ = 5.7 Hz, $^3J_{9,10a}$ = 5.8 Hz, 1 H, 9-H), 5.45 (dd, $^3J_{7,8}$ = 5.4 Hz, $^3J_{8,9}$ = 5.7 Hz, 1 H, 8-H), 5.47 (dd, $^3J_{6,7}$ = 4.8 Hz, $^3J_{7,8}$ = 5.4 Hz, 1 H, 7-H), 6.16 (d, $^3J_{2,3}$ = 15.7 Hz, 1 H, 2-H), 6.59 (dd, $^3J_{3,4}$ = 7.2 Hz, $^3J_{2,3}$ = 15.7 Hz, 1 H, 3-H); ^{13}C-NMR ($CDCl_3$, 100 MHz, 25 °C): δ = 20.9, 21.0, 21.1, 21.1, 21.1 (5 OAc), 52.2 (OMe), 55.0 (4-C), 57.8 (5-C), 61.8 (10-C), 69.2 (9-C), 69.4 (7-C), 69.8 (8-C), 70.4 (6-C), 125.1 (2-C), 143.1 (3-C), 166.1 (1-C), 170.0, 170.0, 170.0, 170.2, 170.8 (5 CO-OAc); HRMS (ESI) *m/z* $[M+Na]^+$ calculated 511.1428, found 511.1432.

Methyl (2*E*)-6,7,8,9,10-Tetra-*O*-acetyl-4-azido-2,3,4-trideoxy-D-glycero-D-ido-nona-2-enonate (5a): Epoxide **4a** (100 mg, 0.24 mmol) was opened with $TMSN_3$ (64 µl, 0.48 mmol) and $Pd(PPh_3)_4$ (28 mg, 0.02 mmol) in 3 ml of degassed THF according to method D. Purification by silica gel chromatography was performed using HE/EA = 2/1 as eluent; yield: 92 mg, (83%) as a mixture of acetate migration products (~2/1/1). IR (neat): 3475, 2923, 2109, 1748, 1437, 1373, 1223, 1044 cm^{-1}; ^{1}H NMR ($CDCl_3$, 400 MHz, 25°C): (main product) δ = 2.05, 2.06, 2.09, 2.12 (4s, 12 H, 4 OAc), 2.93 (d, $^3J_{5,5\text{-}OH}$ = 8.6 Hz, 1 H, 5-OH), 3.70 (ddd, $^3J_{4,5}$ = 4.4 Hz, $^3J_{5,6}$ = 4.5 Hz, 1 H, 5-H), 3.78 (s, 3 H, OMe), 4.09-4.48 (m, 3 H, 4-H, 9a-H, 9b-H), 5.12 (ddd, $^3J_{8,9a}$ = 2.9 Hz, $^3J_{8,9b}$ = 4.2 Hz, $^3J_{7,8}$ = 8.3 Hz, 1 H, 8-H), 5.26 (dd, $^3J_{5,6}$ = 2.0 Hz, $^3J_{6,7}$ = 7.0 Hz, 1 H, 6-H), 5.38 (dd, $^3J_{6,7}$ = 7.0 Hz, $^3J_{7,8}$ = 8.3 Hz, 1 H, 7-H), 6.16 (dd, $^4J_{2,4}$ = 1.3 Hz, $^3J_{2,3}$ = 15.8 Hz, 1 H, 2-H), 6.83 (dd, $^3J_{3,4}$ = 6.1 Hz, $^3J_{2,3}$ = 15.8 Hz, 1 H, 3-H); ^{13}C NMR ($CDCl_3$, 100 MHz, 25°C): δ = 20.93, 20.96, 21.10, 21.19 (4 CH_3), 52.41 (OMe), 62.83 (9-C), 68.58 (4-C), 70.57 (8-C), 71.03 (7-C), 72.58 (6-C), 73.68 (5-C), 125.84 (2-C), 139.86 (3-C), 165.63 (1-C), 170.19, 170.27, 170.35, 170.66 (4 C=O); HRMS (ESI) *m/z* $[M+Na]^+$ calculated 482.1387, found 482.1400.

Methyl (2*E*)-6,7,8,9,10-Penta-*O*-acetyl-4-azido-2,3,4-trideoxy-D-threo-L-galacto-deca-2-enonate (5b): Epoxide **4b** (100 mg, 0.20 mmol) was opened with $TMSN_3$ (54 µl, 0.41 mmol) and $Pd(PPh_3)_4$ (24 mg, 0.02 mmol) in 3 ml of degassed THF according to method D. Purification by silica gel chromatography was performed using HE/EA = 3/2 as eluent; yield: 83 mg, (76%). $[\alpha]^D{}_{20}$ = +12.1° (9.0, CH_2Cl_2); IR (neat): 3483, 2957, 2106, 1744, 1437, 1371, 1210, 1033, 734, 630 cm^{-1}; ^{1}H NMR ($CDCl_3$, 400 MHz, 25°C): δ = 2.02, 2.08, 2.10, 2.14, 2.18 (5s, 15 H, 5 OAc), 3.44 (ddd, $^3J_{4,5}$ = 2.8 Hz, $^3J_{5,5\text{-OH}}$ = 5.4 Hz, $^3J_{5,6}$ = 9.4 Hz, 1 H, 5-H), 3.63 (dd, $^4J_{4,5\text{-OH}}$ = 0.5 Hz, $^3J_{5,5\text{-OH}}$ = 5.4 Hz, 1 H, 5-OH), 3.78 (s, 3 H, OMe), 3.78 (m, 1 H, 4-H), 3.82 (dd, $^3J_{9,10a}$ = 5.4 Hz, 8.0 Hz, $^2J_{10a,10b}$ = 11.9 Hz, 1 H, 10a-H), 4.33 (dd, $^3J_{9,10b}$ = 4.1 Hz, $^2J_{10a,10b}$ = 11.9 Hz, 1 H, 10b-H), 5.22 (dd, $^3J_{8,9}$ = 1.9 Hz, $^3J_{7,8}$ = 10.2 Hz, 1 H, 8-H), 5.25 (dd, $^3J_{6,7}$ = 1.3 Hz, $^3J_{5,6}$ = 9.4 Hz, 1 H, 6-H), 5.36 (dd, $^3J_{6,7}$ = 1.3 Hz, $^3J_{7,8}$ = 10.2 Hz, 1 H, 7-H), 5.36 (ddd, $^3J_{8,9}$ = 1.9 Hz, $^3J_{9,10b}$ = 4.1 Hz, $^3J_{9,10a}$ = 5.4 Hz, 1 H, 9-H), 6.10 (dd, $^4J_{2,4}$ = 1.1 Hz, $^3J_{2,3}$ = 15.7 Hz, 1 H, 2-H), 6.97 (dd, $^3J_{3,4}$ = 7.3 Hz, $^3J_{2,3}$ = 15.7 Hz, 1 H, 3-H); ^{13}C-NMR ($CDCl_3$, 100 MHz, 25 °C): δ = 20.6, 20.6, 20.7, 20.7, 20.9 (5 OAc), 51.9 (OMe), 62.1 (4-C), 62.7 (10-C), 67.3 (7-C), 68.0 (9-C), 68.6 (6-C), 68.9 (8-C), 70.2 (5-C), 124.8 (2-C), 141.4 (3-C), 165.9 (1-C), 169.7, 170.1, 170.4, 170.4, 172.5 (5 CO-OAc); HRMS (ESI) *m/z* $[M+Na]^+$ calculated 554.1598, found 554.1595.

Methyl (2*E*)-6,7,8,9,10-Penta-*O*-acetyl-4-azido-2,3,4-trideoxy-D-erythro-L-galacto-deca-2-enonate (5c): Epoxide **4c** (100 mg, 0.20 mmol) was opened with $TMSN_3$ (54 µl, 0.41 mmol) and $Pd(PPh_3)_4$ (24 mg, 0.02 mmol) in 3 ml of degassed THF according to method D. Purification by silica gel chromatography was performed using HE/EA = 3/2 as eluent; yield 93 mg, (85%). $[\alpha]^D{}_{20}$ = -17.2° (1.5, CH_2Cl_2); IR (neat): 3482, 2957, 2109, 1750, 1654, 1374, 1219, 1047, 717 cm^{-1}; ^{1}H NMR ($CDCl_3$, 400 MHz, 25°C): δ = 2.03, 2.10, 2.11, 2.12, 2.15

(5s, 15 H, 5 OAc), 3.16 (d, $^3J_{5,5\text{-OH}}$ = 7.6 Hz, 1 H, 5-OH), 3.63 (ddd, $^3J_{4,5}$ = 2.7 Hz, $^3J_{5,5\text{-OH}}$ = 7.6 Hz, $^3J_{5,6}$ = 8.8 Hz, 1 H, 5-H), 3.77 (s, 3 H, OMe), 3.95 (ddd, $^4J_{2,4}$ = 1.0 Hz, $^3J_{4,5}$ = 2.7 Hz, $^3J_{3,4}$ = 7.4 Hz, 1 H, 4-H), 4.13 (dd, $^3J_{9,10a}$ = 5.4 Hz, $^2J_{10a,10b}$ = 12.2 Hz, 1 H, 10a-H), 4.29 (dd, $^3J_{9,10b}$ = 4.6 Hz, $^2J_{10a,10b}$ = 12.2 Hz, 1 H, 10b-H), 5.12 (ddd, $^3J_{9,10b}$ = 4.6 Hz, $^3J_{9,10a}$ = 5.4 Hz, $^3J_{8,9}$ = 10.7 Hz, 1 H, 9-H), 5.24 (dd, $^3J_{6,7}$ = 3.4 Hz, $^3J_{5,6}$ = 8.8 Hz, 1 H, 6-H), 5.47 (dd, $^3J_{6,7}$ = 3.4 Hz, $^3J_{7,8}$ = 5.9 Hz, 1 H, 7-H), 5.50 (dd, $^3J_{7,8}$ = 5.9 Hz, $^3J_{8,9}$ = 10.7 Hz, 1 H, 8-H), 6.11 (dd, $^4J_{2,4}$ = 1.0 Hz, $^3J_{2,3}$ = 15.7 Hz, 1 H, 2-H), 6.94 (dd, $^3J_{3,4}$ = 7.4 Hz, $^3J_{2,3}$ = 15.7 Hz, 1 H, 3-H); ^{13}C-NMR ($CDCl_3$, 100 MHz, 25 °C): δ = 20.6, 20.6, 20.6, 20.7, 20.7 (5 OAc), 52.3 (OMe), 61.7 (10-C), 63.2 (4-C), 69.3 (9-C), 69.8 (6-C), 70.2 (7-C), 70.6 (8-C), 71.9 (5-C), 125.4 (2-C), 141.4 (3-C), 166.1 (1-C), 170.1, 170.1, 170.6, 170.9, 171.5 (5 CO-OAc); HRMS (ESI) *m/z* $[M+Na]^+$ calculated 554.1598, found 554.1609.

2-Azido-2-deoxy-D-glycero-D-ido-heptose (6a): Azide **5a** (157 mg, 0.34 mmol) was deacetylated in a methanolic HCl solution using AcCl (73 μl, 1.03 mmol) in 9 ml of dry MeOH according to method E. After ozonolysis, the reaction was quenched with PPh_3 (108 mg, 0.41 mmol). Purification by silica gel chromatography was performed using DCM/MeOH = 6/1 as eluent; yield: 66 mg (mixture of anomers/conformers), (82%). $[\alpha]^D_{20}$ = -26.3° (4.2, H_2O); IR (neat): 3340, 2926, 2117, 1641, 1263, 1042, 813, 737, 631 cm^{-1}; ^{1}H NMR (D_2O, 600 MHz, 25 °C): (1-H) δ = 4.98 (d, $^3J_{1,2}$ = 8.7 Hz), 5.01 (d, $^3J_{1,2}$ = 4.9 Hz), 5.18 (d, $^3J_{1,2}$ = 1.4 Hz), 5.25 (d, $^3J_{1,2}$ = 3.5 Hz), 5.52 (d, $^3J_{1,2}$ = 4.7 Hz), ^{13}C NMR (D_2O, 150 MHz, 25°C): (1-C) δ = 92.4, 93.1, 93.3, 94.8, 99.0; HRMS (ESI) *m/z* $[M+Na]^+$ calculated 258.0702, found 258.0701.

2-Azido-2-deoxy-D-threo-L-galacto-octose (6b): Azide **5b** (155 mg, 0.29 mmol) was deacetylated in a methanolic HCl solution using AcCl (62 μl, 0.87 mmol) in 9 ml of dry MeOH according to method E. Since the deacetylation product was not completely soluble in

MeOH about 1.5 mmol of ozone were bubbled through the suspension. After 1 h at -78°C additional 0.75 mmol of ozone were added. After 1 h at -78°C the reaction was quenched with PPh_3 (92 mg, 0.35 mmol). Purification by silica gel chromatography was performed using DCM/MeOH = 6/1 as eluent; yield: 55 mg (mixture of anomers, α/β = 1/2), (71%), 3 mg (3%) of not ozonolyzed deacetylation product were recovered. $[\alpha]^{D}_{20}$ = +17.9° (2.9, H_2O); IR (neat): 3341, 2927, 2118, 1591, 1350, 1064, 770, 630 cm^{-1}; ^1H NMR (D_2O, 600 MHz, 25 °C): (β-anomer) δ = 3.49 (dd, $^3J_{2,3}$ = 10.6 Hz, $^3J_{1,2}$ = 8.1 Hz, 1 H, 2-H), 3.61 (dd, $^3J_{5,6}$ = 9.4 Hz, $^3J_{4,5}$ = 0.9 Hz, 1 H, 5-H), 3.71 (m, 3 H, 8a-H, 8b-H, 3-H), 3.83 (dd, $^3J_{6,7}$ = 1.5 Hz, $^3J_{5,6}$ = 9.4 Hz, 1 H, 6-H), 3.92 (ddd, $^3J_{6,7}$ = 1.5 Hz, $^3J_{7,8a}$ = 5.6 Hz, $^3J_{7,8b}$ = 7.2 Hz, 1 H, 7-H), 4.09 (dd, $^3J_{4,5}$ = 0.9 Hz, $^3J_{3,4}$ = 3.4 Hz, 1 H, 4-H), 4.65 (d, $^3J_{1,2}$ = 8.1 Hz, 1 H, 1-H), (α-anomer) δ = 3.71 (m, 3 H, 8a-H, 8b-H, 2-H), 3.81 (dd, $^3J_{5,6}$ = 9.6 Hz, $^3J_{6,7}$ = 1.7 Hz, 1 H, 6-H), 3.87 (ddd, $^3J_{6,7}$ = 1.7 Hz, $^3J_{7,8b}$ = 5.4 Hz, $^3J_{7,8a}$ = 7.3 Hz, 1 H, 7-H), 4.02 (dd, $^3J_{3,4}$ = 3.2 Hz, $^3J_{2,3}$ = 10.8 Hz, 1 H, 3-H), 4.04 (dd, $^3J_{4,5}$ = 0.9 Hz, $^3J_{5,6}$ = 9.6 Hz, 1 H, 5-H), 4.18 (dd, $^3J_{4,5}$ = 0.9 Hz, $^3J_{3,4}$ = 3.2 Hz, 1 H, 4-H), 5.37 (d, $^3J_{1,2}$ = 3.8 Hz, 1 H, 1-H), ^{13}C NMR (D_2O, 150 MHz, 25°C): (β-anomer) δ = 63.0 (8-C), 64.7 (2-C), 67.0 (4-C), 67.4 (6-C), 70.0 (7-C), 72.0 (3-C), 73.0 (5-C), 95.6 (1-C), (α-anomer): 60.5 (2-C), 63.0 (8-C), 67.7 (6-C), 67.9 (4-C), 68.4 (3-C), 68.4 (5-C), 70.1 (7-C), 91.4 (1-C); HRMS (ESI) *m/z* $[M+Na]^+$ calculated 288.0808, found 288.0791.

2-Azido-2-deoxy-D-erythro-L-galacto-octose (6c): Azide **5c** (102 mg, 0.19 mmol) was deacetylated in a methanolic HCl solution using AcCl (41 µl, 0.58 mmol) in 6 ml of dry MeOH according to method E. After ozonolysis the reaction was quenched with PPh_3 (60 mg, 0.23 mmol). Purification by silica gel chromatography was performed using DCM/MeOH= 6/1 as eluent; yield: 38 mg (mixture of anomers, α/β = 1/2), (75%). $[\alpha]^{D}_{20}$ = -35.6° (12.9, H_2O); IR (neat): 3339, 2923, 2121, 1641, 1252, 1017, 723, 633 cm^{-1}; ^1H NMR (D_2O, 600 MHz, 25°C): (β-anomer) δ = 3.52 (dd, $^3J_{1,2}$ = 8.1 Hz, $^3J_{2,3}$ = 10.4 Hz, 1 H, 2-H), 3.66 (dd, $^3J_{7,8a}$ = 6.5 Hz, $^2J_{8a,8b}$ = 11.8 Hz, 1 H, 8a-H), 3.67 (dd, $^3J_{3,4}$ = 3.3 Hz, $^3J_{2,3}$ = 10.4 Hz,

1 H, 3-H), 3.69 (dd, $^3J_{4,5}$ = 0.9 Hz, $^3J_{5,6}$ = 5.3 Hz, 1 H, 5-H), 3.77 (dd, $^3J_{7,8b}$ = 3.3 Hz, $^2J_{8a,8b}$ = 11.8 Hz, 1 H, 8b-H), 3.82 (ddd, $^3J_{7,8b}$ = 3.3 Hz, $^3J_{6,7}$ = 5.9 Hz, $^3J_{7,8a}$ = 6.5 Hz, 1 H, 7-H), 3.94 (dd, $^3J_{5,6}$ = 5.3 Hz, $^3J_{6,7}$ = 5.9 Hz, 1 H, 6-H), 4.04 (dd, $^3J_{3,4}$ = 3.3 Hz, $^3J_{4,5}$ = 0.9 Hz, 1 H, 4-H), 4.65 (d, $^3J_{1,2}$ = 8.1 Hz, 1 H, 1-H), (α-anomer) δ = 3.67 (dd, $^3J_{7,8a}$ = 6.5 Hz, $^2J_{8a,8b}$ = 11.8 Hz, 1 H, 8a-H), 3.77 (dd, $^3J_{7,8b}$ = 3.3 Hz, $^2J_{8a,8b}$ = 11.8 Hz, 1 H, 8b-H), 3.73 (dd, $^3J_{1,2}$ = 3.8 Hz, $^3J_{2,3}$ = 10.7 Hz, 1 H, 2-H), 3.80 (ddd, $^3J_{7,8b}$ = 3.3 Hz, $^3J_{6,7}$ = 5.9 Hz, $^3J_{7,8a}$ = 6.5 Hz, 1 H, 7-H), 3.93 (dd, $^3J_{5,6}$ = 5.3 Hz, $^3J_{6,7}$ = 5.9 Hz, 1 H, 6-H), 4.01 (dd, $^3J_{3,4}$ = 3.1 Hz, $^3J_{2,3}$ = 10.7 Hz, 1 H, 3-H), 4.13 (dd, $^3J_{3,4}$ = 3.1 Hz, $^3J_{4,5}$ = 0.9 Hz, 1 H, 4-H), 4.13 (dd, $^3J_{4,5}$ = 0.9 Hz, $^3J_{5,6}$ = 5.3 Hz, 1 H, 5-H), 5.42 (d, $^3J_{1,2}$ = 3.8 Hz, 1 H, 1-H), ^{13}C NMR ($CDCl_3$, 150 MHz, 25°C): (β-anomer) δ = 61.9 (8-C), 64.5 (2-C), 69.5 (4-C), 70.8 (7-C), 71.7 (3-C), 72.3 (6-C), 73.3 (5-C), 95.6 (1-C), (α-anomer) δ = 60.3 (2-C), 62.1 (8-C), 68.1 (3-C), 68.4 (5-C), 70.7 (4-C), 70.8 (7-C), 72.5 (6-C), 91.3 (1-C); HRMS (ESI) *m/z* $[M+Na]^+$ calculated 288.0808, found 288.0808.

2-Acetamido-1,3,4,6,7-penta-*O*-acetyl-2-deoxy-D-glycero-D-ido-heptose (7a): Sugar azide **6a** (31 mg, 0.13 mmol) was peracetylated and reduced with DTT (82 mg, 0.53 mmol) and DIPA (1 ml) in 4 ml of dry MeCN according to method F. Purification by silica gel chromatography was performed using HE/EA = 1/3 as eluent; yield: 20 mg, ($^4C_1$-pyranoid form, mixture of anomers, α/β = 3/2), (33%), 16 mg (β-furanoid form/$^1C_4$ α-pyranoid form = 5/2), (26%). ^{1}H NMR ($CDCl_3$, 600 MHz, 25°C): ($^4C_1$-pyranoid form), (α-anomer) δ = 2.01, 2.02, 2.05, 2.10, 2.12, 2.14 (6s, 18 H, 6 Ac), 4.10 (dd, $^3J_{6,7a}$ = 4.6 Hz, $^2J_{7a,7b}$ = 12.4 Hz, 1 H, 7a-H), 4.33 (dddd, $^4J_{2,4}$ = 1.0 Hz, $^3J_{1,2}$ = 1.8 Hz, $^3J_{2,3}$ = 3.1 Hz, $^3J_{2,2\text{-}NH}$ = 9.8 Hz, 1 H, 2-H), 4.38 (ddd, $^4J_{5,1}$ = 0.6 Hz, $^3J_{4,5}$ = 1.9 Hz, $^3J_{5,6}$ = 9.9 Hz, 1 H, 5-H), 4.46 (dd, $^3J_{6,7b}$ = 2.4 Hz, $^2J_{7a,7b}$ = 12.4 Hz, 1 H, 7b-H), 4.84 (ddd, $^4J_{3,1}$ = 1.1 Hz, $^3J_{2,3}$ = 3.1 Hz, $^3J_{3,4}$ = 3.1 Hz, 1 H, 3-H), 5.08 (dddd, $^5J_{4,1}$ = 0.7 Hz, $^4J_{2,4}$ = 1.0 Hz, $^3J_{4,5}$ = 1.9 Hz, $^3J_{3,4}$ = 3.1 Hz, 1 H, 4-H), 5.12 (ddd, $^3J_{6,7b}$ = 2.4 Hz, $^3J_{6,7a}$ = 4.6 Hz, $^3J_{5,6}$ = 9.9 Hz, 1 H, 6-H), 5.92 (dddd, $^4J_{5,1}$ = 0.6 Hz, $^5J_{4,1}$ = 0.7 Hz, $^4J_{3,1}$ = 1.1 Hz, $^3J_{1,2}$ = 1.8 Hz, 1 H, 1-H), 6.05 (d, $^3J_{2,2\text{-}NH}$ = 9.8 Hz, 1 H, NH), ($^4C_1$-pyranoid

form), (β-anomer) δ = 2.01, 2.05, 2.09, 2.09, 2.10, 2.17, (6s, 18 H, 6 Ac), 4.13 (dd, $^3J_{6,7a}$ = 5.0 Hz, $^2J_{7a,7b}$ = 12.4 Hz, 1 H, 7a-H), 4.20 (dd, $^3J_{4,5}$ = 1.8 Hz, $^3J_{5,6}$ = 9.8 Hz, 1 H, 5-H), 4.35 (dddd, $^4J_{2,4}$ = 1.0 Hz, $^3J_{1,2}$ = 2.1 Hz, $^3J_{2,3}$ = 2.9 Hz, $^3J_{2,2\text{-NH}}$ = 9.7 Hz, 1 H, 2-H), 4.43 (dd, $^3J_{6,7b}$ = 2.3 Hz, $^2J_{7a,7b}$ = 12.4 Hz, 1 H, 7b-H), 4.97 (ddd, $^4J_{2,4}$ = 1.0 Hz, $^3J_{3,4}$ = 2.9 Hz, $^3J_{2,3}$ = 2.9 Hz, 1 H, 4-H), 5.00 (dd, $^3J_{2,3}$ = 2.9 Hz, $^3J_{3,4}$ = 2.9 Hz, 1 H, 3-H), 5.18 (ddd, $^3J_{6,7b}$ = 2.3 Hz, $^3J_{6,7a}$ = 5.0 Hz, $^3J_{5,6}$ = 9.8 Hz, 1 H, 6-H), 5.94 (d, $^3J_{1,2}$ = 2.1 Hz, 1 H, 1-H), 6.10 (d, $^3J_{2,2\text{-NH}}$ = 9.7 Hz, 1 H, 2-NH), (β-furanoid form) δ = 2.00, 2.04, 2.04, 2.10, 2.12, 2.16 (6s, 18 H, 6 Ac), 4.10 (dd, $^3J_{6,7a}$ = 6.7 Hz, $^2J_{7a,7b}$ = 12.4 Hz, 1 H, 7a-H), 4.37 (dd, $^3J_{6,7b}$ = 3.0 Hz, $^2J_{7a,7b}$ = 12.4 Hz, 1 H, 7b-H), 4.55 (ddd, $^3J_{1,2}$ = 3.1 Hz, $^3J_{2,3}$ = 5.1 Hz, $^3J_{2,2\text{-NH}}$ = 8.1 Hz, 1 H, 2-H), 4.59 (dd, $^3J_{4,5}$ = 5.1 Hz, $^3J_{3,4}$ = 6.8 Hz, 1 H, 4-H), 5.13 (ddd, $^3J_{6,7b}$ = 3.0 Hz, $^3J_{5,6}$ = 5.1 Hz, $^3J_{6,7a}$ = 6.7 Hz, 1 H, 6-H), 5.32 (dd, $^3J_{3,4}$ = 2.9 Hz, $^3J_{2,3}$ = 2.9 Hz, 1 H, 3-H), 5.36 (dd, $^3J_{4,5}$ = 5.1 Hz, $^3J_{5,6}$ = 9.8 Hz, 1 H, 5-H), 5.93 (d, $^3J_{2,2\text{-NH}}$ = 8.1 Hz, 1 H, 2-NH), 6.03 (d, $^3J_{1,2}$ = 2.1 Hz, 1 H, 1-H), ($^1C_4$ α-pyranoid form) δ = 1.95, 2.01, 2.07, 2.12, 2.13, 2.22 (6s, 18 H, 6 Ac), 4.11 (dd, $^3J_{4,5}$ = 1.5 Hz, $^3J_{5,6}$ = 9.6 Hz, 1 H, 5-H), 4.14 (dd, $^3J_{6,7a}$ = 5.1 Hz, $^2J_{7a,7b}$ = 12.3 Hz, 1 H, 7a-H), 4.42 (dd, $^3J_{6,7b}$ = 2.4 Hz, $^2J_{7a,7b}$ = 12.3 Hz, 1 H, 7b-H), 4.54 (ddd, $^3J_{2,3}$ = 3.4 Hz, $^3J_{1,2}$ = 9.3 Hz, $^3J_{2,2\text{-NH}}$ = 9.3 Hz, 1 H, 2-H), 5.04 (dd, $^3J_{4,5}$ = 1.5 Hz, $^3J_{3,4}$ = 3.6 Hz, 1 H, 4-H), 5.15 (ddd, $^3J_{6,7b}$ = 2.4 Hz, $^3J_{6,7a}$ = 5.1 Hz, $^3J_{5,6}$ = 9.6 Hz, 1 H, 6-H), 5.16 (dd, $^3J_{2,3}$ = 3.4 Hz, $^3J_{3,4}$ = 3.6 Hz, 1 H, 3-H), 5.47 (d, $^3J_{2,2\text{-NH}}$ = 9.3 Hz, 1 H, 2-NH), 5.84 (d, $^3J_{1,2}$ = 9.3 Hz, 1 H, 1-H), ^{13}C NMR ($CDCl_3$, 150 MHz, 25°C): ($^4C_1$-pyranoid form), (α-anomer) δ = 20.6, 20.7, 20.7, 20.7, 20.8, 23.2 (6 CH_3), 45.7 (2-C), 62.2 (7-C), 65.0 (4-C), 65.2 (5-C), 67.1 (3-C), 67.2 (6-C), 91.8 ($^1J_{1C,1H}$ = 177 Hz, 1-C), 168.1, 168.5, 168.8, 168.8, 169.7, 170.3 (6 CO-Ac), ($^4C_1$-pyranoid form), (β-anomer) δ = 20.6, 20.7, 20.7, 20.6, 20.8, 23.3 (6 Ac), 47.0 (2-C), 62.4 (7-C), 64.2 (4-C), 67.0 (6-C), 68.7 (3-C), 72.3 (5-C), 90.7 ($^1J_{1C,1H}$ = 166 Hz, 1-C), 168.2, 168.3, 168.6, 169.4, 169.8, 170.6 (6 CO-Ac), (β-furanoid form) δ = 20.6, 20.7, 20.8, 20.9, 21.1, 23.1 (6 Ac), 60.0 (2-C), 61.8 (7-C), 69.2 (5-C), 70.1 (6-C), 74.8 (4-C), 78.3 (3-C), 98.5 ($^1J_{1C,1H}$ = 179 Hz, 1-C), 169.5, 169.8, 170.0,

170.1, 170.3, 170.7 (6 CO-Ac), ($^1C_4$ α-pyranoid form) δ = 20.7, 20.7, 20.7, 20.9, 21.0, 23.2 (6 Ac), 47.3 (2-C), 62.5 (7-C), 64.8 (4-C), 67.3 (6-C), 70.0 (3-C), 71.1 (5-C), 91.4 ($^1J_{1C,1H}$ = 166 Hz, 1-C), 168.8, 169.4, 169.6, 169.6, 170.0, 170.6 (6 CO-Ac); HRMS (ESI) *m/z* $[M+Na]^+$ calculated 484.1431, found (7a) 484.1419, (7a') 484.1425.

2-Acetamido-1,3,4,6,7,8-hexa-*O*-acetyl-2-deoxy-D-threo-L-galacto-octose (7b): Sugar azide **6b** (55 mg, 0.21 mmol) was peracetylated and reduced with DTT (128 mg, 0.83 mmol) and DIPA (1.5 ml) in 6 ml of dry MeCN according to method F. Purification by silica gel chromatography was performed using HE/EA = 1/4 as eluent; yield 73 mg (mixture of anomers, α/β = 1/1), (66%). ^{1}H NMR ($CDCl_3$, 400 MHz, 25°C): (β-anomer) δ = 1.93, 2.00, 2.01, 2.01, 2.04, 2.12, 2.13 (7s, 21 H, 7 Ac), 3.87 (dd, $^3J_{4,5}$ = 1.1 Hz, $^3J_{5,6}$ = 9.5 Hz, 1 H, 5-H), 3.96 (dd, $^2J_{8a,8b}$ = 11.8 Hz, $^3J_{7,8a}$ = 5.8 Hz, 1 H, 8a-H), 4.17 (dd, $^3J_{7,8b}$ = 4.8 Hz, $^2J_{8a,8b}$ = 11.8 Hz, 1 H, 8b-H), 4.36 (ddd, $^3J_{1,2}$ = 9.0 Hz, $^3J_{2,2\text{-NH}}$ = 9.1 Hz, $^3J_{2,3}$ = 11.3 Hz, 1 H, 2-H), 5.12 (dd, $^3J_{3,4}$ = 3.5 Hz, $^3J_{2,3}$ = 11.3 Hz, 1 H, 3-H), 5.30 (d, $^3J_{2,2\text{-NH}}$ = 9.1 Hz, 1 H, 2-NH), 5.35 (m, 3 H, 4-H, 6-H, 7-H), 5.62 (d, $^3J_{1,2}$ = 9.0 Hz, 1 H, 1-H), (α-anomer) δ = 1.94, 2.01, 2.02, 2.10, 2.12, 2.12, 2.15 (7s, 21 H, 7 Ac), 3.94 (dd, $^3J_{7,8a}$ = 7.1 Hz, $^2J_{8a,8b}$ = 11.8 Hz, 1 H, 8a-H), 4.06 (dd, $^3J_{4,5}$ = 0.9 Hz, $^3J_{5,6}$ = 9.7 Hz, 1 H, 5-H), 4.21 (dd, $^3J_{7,8b}$ = 5.6 Hz, $^2J_{8a,8b}$ = 11.8 Hz, 1 H, 8b-H), 4.72 (ddd, $^3J_{1,2}$ = 3.7 Hz, $^3J_{2,2\text{-NH}}$ = 9.3 Hz, $^3J_{2,3}$ = 11.5 Hz, 1 H, 2-H), 5.15 (dd, $^3J_{6,7}$ = 2.0 Hz, $^3J_{5,6}$ = 9.7 Hz, 1 H, 6-H), 5.18 (dd, $^3J_{3,4}$ = 3.3 Hz, $^3J_{2,3}$ = 11.5 Hz, 1 H, 3-H), 5.31 (d, $^3J_{2,2\text{-NH}}$ = 9.3 Hz, 1 H, 2-NH), 5.35 (m, 2 H, 4-H, 7-H), 6.23 (d, $^3J_{1,2}$ = 3.7 Hz, 1 H, 1-H); ^{13}C-NMR ($CDCl_3$, 100 MHz, 25 °C): (β-anomer) δ = 20.5, 20.6, 20.6, 20.7, 20.7, 20.8, 23.7 (7 Ac), 50.1 (2-C), 62.9 (8-C), 65.1 (6-C), 65.6 (4-C), 68.7 (7-C), 70.3 (3-C), 71.6 (5-C), 93.2 (1-C), 169.2, 169.5, 169.9, 170.2, 170.4, 170.7, 171.2 (7 CO-Ac), (α-anomer) δ = 20.4, 20.6, 20.6, 20.6, 20.7, 20.8, 23.6 (7 Ac), 47.1 (2-C), 62.1 (8-C), 67.1 (4-C), 67.4 (6-C), 68.1 (3-C), 68.2 (5-C), 68.2 (7-C), 91.2 (1-C), 168.5, 169.4, 169.5, 169.6, 170.2, 170.4, 170.6 (7 CO-Ac); HRMS (ESI) *m/z* $[M+Na]^+$ calculated 556.1642, found 556.1633.

2-Acetamido-1,3,4,6,7,8-hexa-*O*-acetyl-2-deoxy-D-erythro-L-galacto-octose (7c): Sugar azide **6c** (38 mg, 0.14 mmol) was peracetylated and reduced with DTT (88 mg, 0.57 mmol)

and DIPA (1 ml) in 4 ml of dry MeCN according to method F. Purification by silica gel chromatography was performed using HE/EA = 1/4 as eluent; yield: 51 mg, (mixture of anomers, α/β = 1/2), (67%). $[\alpha]^{D}_{20}$ = -12.0° (1.0, CH_2Cl_2); ^{1}H NMR ($CDCl_3$, 400 MHz, 25°C):, (β-anomer) δ = 1.94, 2.01, 2.01, 2.07, 2.11, 2.11, 2.23 (7s, 21 H, 7 Ac), 3.90 (dd, $^3J_{4,5}$ = 0.5 Hz, $^3J_{5,6}$ = 7.6 Hz, 1 H, 5-H), 4.17 (dd, $^3J_{7,8a}$ = 5.9 Hz, $^2J_{8a,8b}$ = 11.9 Hz, 1 H, 8a-H), 4.28 (dd, $^3J_{7,8b}$ = 5.1 Hz, $^2J_{8a,8b}$ = 11.9 Hz, 1 H, 8b-H), 4.32 (ddd, $^3J_{1,2}$ = 8.8 Hz, $^3J_{2,2\text{-NH}}$ = 9.4 Hz, $^3J_{2,3}$ = 11.1 Hz, 1 H, 2-H), 4.95 (ddd, $^3J_{6,7}$ = 3.5 Hz, $^3J_{7,8b}$ = 5.1 Hz, $^3J_{7,8a}$ = 5.9 Hz, 1 H, 7-H), 5.14 (dd, $^3J_{3,4}$ = 3.3 Hz, $^3J_{2,3}$ = 11.1 Hz, 1 H, 3-H), 5.32 (d, $^3J_{2,2\text{-NH}}$ = 9.4 Hz, 1 H, 2-NH), 5.46 (dd, $^3J_{6,7}$ = 3.5 Hz, $^3J_{5,6}$ = 7.6 Hz, 1 H, 6-H), 5.52 (dd, $^3J_{4,5}$ = 0.5 Hz, $^3J_{3,4}$ = 3.3 Hz, 1 H, 4-H), 5.65 (d, $^3J_{1,2}$ = 8.8 Hz, 1 H, 1-H), (α-anomer) δ = 1.95, 2.02, 2.02, 2.07, 2.08, 2.18, 2.21 (7s, 21 H, 7 Ac), 4.05 (dd, $^3J_{4,5}$ = 1.0 Hz, $^3J_{5,6}$ = 6.6 Hz, 1 H, 5-H), 4.14 (dd, $^3J_{7,8a}$ = 6.3 Hz, $^2J_{8a,8b}$ = 12.0 Hz, 1 H, 8a-H), 4.28 (dd, $^3J_{7,8b}$ = 5.1 Hz, $^2J_{8a,8b}$ = 12.0 Hz, 1 H, 8b-H), 4.72 (ddd, $^3J_{1,2}$ = 3.7 Hz, $^3J_{2,2\text{-NH}}$ = 9.2 Hz, $^3J_{2,3}$ = 11.6 Hz, 1 H, 2-H), 5.00 (ddd, $^3J_{6,7}$= 4.1 Hz, $^3J_{7,8b}$ = 5.1 Hz, $^3J_{7,8a}$ = 6.3 Hz, 1 H, 7-H), 5.16 (dd, $^3J_{3,4}$ = 3.3 Hz, $^3J_{2,3}$ = 11.6 Hz, 1 H, 3-H), 5.32 (d, $^3J_{2,2\text{-NH}}$ = 9.2 Hz, 1 H, 2-NH), 5.37 (dd, $^3J_{6,7}$= 4.1 Hz, $^3J_{5,6}$ = 6.6 Hz, 1 H, 6-H), 5.53 (dd, $^3J_{4,5}$ = 1.0 Hz, $^3J_{3,4}$ = 3.3 Hz, 1 H, 4-H), 6.19 (d, $^3J_{1,2}$ = 3.7 Hz, 1 H, 1-H); ^{13}C-NMR ($CDCl_3$, 100 MHz, 25 °C): (β-anomer) δ = 20.6, 20.6, 20.7, 20.7, 20.8, 20.9, 23.4 (7 Ac), 50.2 (2-C), 61.1 (8-C), 66.2 (4-C), 69.2 (7-C), 69.9 (6-C), 70.3 (3-C), 73.3 (5-C), 93.2 (1-C), 169.6, 169.8, 170.1, 170.2, 170.5, 170.6, 170.9 (7 CO-OAc), (α-anomer) δ = 20.6, 20.6, 20.7, 20.7, 20.8, 20.9, 23.2 (7 Ac), 46.9 (2-C), 61.4 (8-C), 67.0 (4-C), 68.1 (3-C), 69.0 (5-C), 69.5 (6-C), 69.6 (7-C), 91.6 (1-C), 169.7, 169.8, 170.9, 170.3, 170.5, 170.52, 170.5 (7 CO-OAc); HRMS (ESI) *m/z* $[M+Na]^+$ calculated 556.1642, found 556.1638.

2-Acetamido-2-deoxy-D-glycero-D-ido-heptose (8a): Peracetylated amino sugar **7a** (20 mg, 0.04 mmol) was deacetylated according to method G in 3 ml of dry MeOH; yield: 11 mg, (100%). $[\alpha]^{D}_{20}$ = -12.8° (2.5, H_2O); ^{1}H NMR (D_2O, 600 MHz, 25 °C): (1-H) δ = 4.92 (d, $^3J_{1,2}$

= 8.9 Hz), 5.05 (d, $^3J_{1,2}$ = 3.0 Hz), 5.15 (d, $^3J_{1,2}$ = 3.8 Hz), 5.19 (d, $^3J_{1,2}$ = 1.9 Hz), 5.45 (d, $^3J_{1,2}$ = 4.9 Hz), ^{13}C NMR (D_2O, 150 MHz, 25°C): (1-C) δ = 91.4, 91.8, 92.9, 93.5, 93.6; HRMS (ESI) *m/z* $[M+Na]^+$ calculated 274.0903, found 274.0905.

2-Acetamido-2-deoxy-D-threo-L-galacto-octose (8b): Peracetylated amino sugar **7b** (44 mg, 0.08 mmol) was deacetylated according to method G in 4 ml of dry methanol; yield: 23 mg, (mixture of anomers, α/β = 1/1), (100%). $[\alpha]^D_{20}$ = -30° (5.0, H_2O), ^{1}H NMR (MeOD, 600 MHz, 25°C): (β-anomer) δ = 2.00 (s, 3 H, NHAc), 3.52 (dd, $^3J_{4,5}$ = 1.0 Hz, $^3J_{5,6}$ = 9.0 Hz, 1 H, 5-H), 3.58 (dd, $^3J_{3,4}$ = 3.4 Hz, $^3J_{2,3}$ = 10.8 Hz, 1 H, 3-H), 3.65 (m, 2 H, 8a-H, 8b-H), 3.88 (m, 3 H, 2-H, 6-H, 7-H), 4.04 (dd, $^3J_{4,5}$ = 1.0 Hz, $^3J_{3,4}$ = 3.4 Hz, 1 H, 4-H), 4.55 (d, $^3J_{1,2}$ = 8.4 Hz, 1 H, 1-H), (α-anomer) δ = 2.00 (s, 3 H, NHAc) 3.65 (m, 2 H, 8a-H, 8b-H), 3.83 (dd, $^3J_{3,4}$ = 3.2 Hz, $^3J_{2,3}$ = 10.9 Hz, 1 H, 3-H), 3.83 (ddd, $^3J_{6,7}$ = 1.6 Hz, $^3J_{7,8a}$ = 6.5 Hz, $^3J_{7,8b}$ = 6.5 Hz, 1 H, 7-H), 3.85 (dd, $^3J_{6,7}$ = 1.6 Hz, $^3J_{5,6}$ = 9.2 Hz, 1 H, 6-H), 4.03 (dd, $^3J_{4,5}$ = 1.2 Hz, $^3J_{5,6}$ = 9.2 Hz, 1 H, 5-H), 4.10 (dd, $^3J_{4,5}$ = 1.2 Hz, $^3J_{3,4}$ = 3.2 Hz, 1 H, 4-H), 4.22 (dd, $^3J_{1,2}$ = 3.7 Hz, $^3J_{2,3}$ = 10.9 Hz, 1 H, 2-H), 5.14 (d, $^3J_{1,2}$ = 3.7 Hz, 1 H, 1-H), ^{13}C NMR (MeOD, 150 MHz, 25°C): (β-anomer) δ = 22.9 (NHAc), 55.9 (2-C), 64.8 (8-C), 68.7 (4-C), 69.1 (6-C), 71.7 (7-C), 73.6 (3-C), 74.8 (5-C), 97.6 (1-C), 174.7 (CO-NHAc), (α-anomer) δ = 22.7 (NHAc), 52.1 (2-C), 64.8 (8-C), 69.5 (6-C), 69.6 (4-C), 69.9 (3-C), 70.0 (5-C), 71.8 (7-C), 93.0 (1-C), 174.0 (CO-NHAc); HRMS (ESI) *m/z* $[M+Na]^+$ calculated 304.1008, found 304.1003.

2-Acetamido-2-deoxy-D-erythro-L-galacto-octose (8c):

Peracetylated amino sugar **7c** (51 mg, 0.10 mmol) was deacetylated according to method G in 5 ml of dry methanol; yield: 27 mg, (mixture of anomers, α/β = 1/1), (100%). $[\alpha]^D_{20}$ = -31.6° (7.9, H_2O); ^{1}H NMR (D_2O, 600 MHz, 25°C): (β-anomer) δ = 2.06 (s, 3 H, NHAc), 3.67 (dd, $^3J_{7,8a}$ = 6.7 Hz, $^2J_{8a,8b}$ = 12.0 Hz, 1 H, 8a-H), 3.70 (dd, $^3J_{4,5}$ = 1.1 Hz, $^3J_{5,6}$ = 5.3 Hz, 1 H, 5-H), 3.72 (dd, $^3J_{3,4}$ = 3.3 Hz, $^3J_{2,3}$ = 10.8 Hz, 1 H, 3-H), 3.78 (dd, $^3J_{7,8b}$ = 3.5 Hz, $^2J_{8a,8b}$ = 12.0 Hz,

1 H, 8b-H), 3.83 (ddd, $^3J_{7,8b}$ = 3.5 Hz, $^3J_{6,7}$ = 6.1 Hz, $^3J_{7,8a}$ = 6.7 Hz, 1 H, 7-H), 3.92 (dd, $^3J_{1,2}$ = 8.5 Hz, $^3J_{2,3}$ = 10.8 Hz, 1 H, 2-H), 3.96 (dd, $^3J_{5,6}$ = 5.3 Hz, $^3J_{6,7}$ = 6.1 Hz, 1 H, 6-H), 4.06 (dd, $^3J_{4,5}$ = 1.1 Hz, $^3J_{3,4}$ = 3.3 Hz, 1 H, 4-H), 4.66 (d, $^3J_{1,2}$ = 8.5 Hz, 1 H, 1-H), (α-anomer) δ = 2.06 (s, 3 H, NHAc) 3.67 (dd, $^3J_{7,8a}$ = 6.5 Hz, $^2J_{8a,8b}$ = 11.9 Hz, 1 H, 8a-H), 3.78 (dd, $^3J_{7,8b}$ = 3.5 Hz, $^2J_{8a,8b}$ = 11.9 Hz, 1 H, 8b-H), 3.81 (ddd, $^3J_{7,8b}$ = 3.5 Hz, $^3J_{6,7}$ = 6.2 Hz, $^3J_{7,8a}$ = 6.5 Hz, 1 H, 7-H), 3.92 (dd, $^3J_{3,4}$ = 3.2 Hz, $^3J_{2,3}$ = 10.9 Hz, 1 H, 3-H), 3.94 (dd, $^3J_{5,6}$ = 5.0 Hz, $^3J_{6,7}$ = 6.2 Hz, 1 H, 6-H), 4.12 (dd, $^3J_{4,5}$ = 1.3 Hz, $^3J_{3,4}$ = 3.2 Hz, 1 H, 4-H), 4.14 (dd, $^3J_{4,5}$ = 1.3 Hz, $^3J_{5,6}$ = 5.0 Hz, 1 H, 5-H), 4.18 (dd, $^3J_{1,2}$ = 3.8 Hz, $^3J_{2,3}$ = 10.9 Hz, 1 H, 2-H), 5.28 (d, $^3J_{1,2}$ = 3.8 Hz, 1 H, 1-H), ^{13}C NMR (D_2O, 150 MHz, 25°C): (β-anomer) δ = 22.2 (NHAc), 53.5 (2-C), 61.8 (8-C), 69.4 (4-C), 70.1 (7-C), 71.2 (3-C), 72.4 (6-C), 73.2 (5-C), 95.5 (1-C), 175.0 (CO-NHAc), (α-anomer) δ = 22.0 (NHAc), 50.1 (2-C), 62.1 (8-C), 67.5 (3-C), 68.4 (5-C), 70.3 (4-C), 71.0 (7-C), 72.6 (6-C), 91.0 (1-C), 174.7 (CO-NHAc), HRMS (ESI) *m/z* $[M+Na]^+$ calculated 304.1008, found 304.1002.

References

1. Albler, C.; Schmid, W. *Eur. J. Org. Chem.* **2014**, 2451-2459.

Copies of ^{1}H and ^{13}C NMR spectra

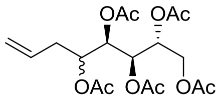

(2a)

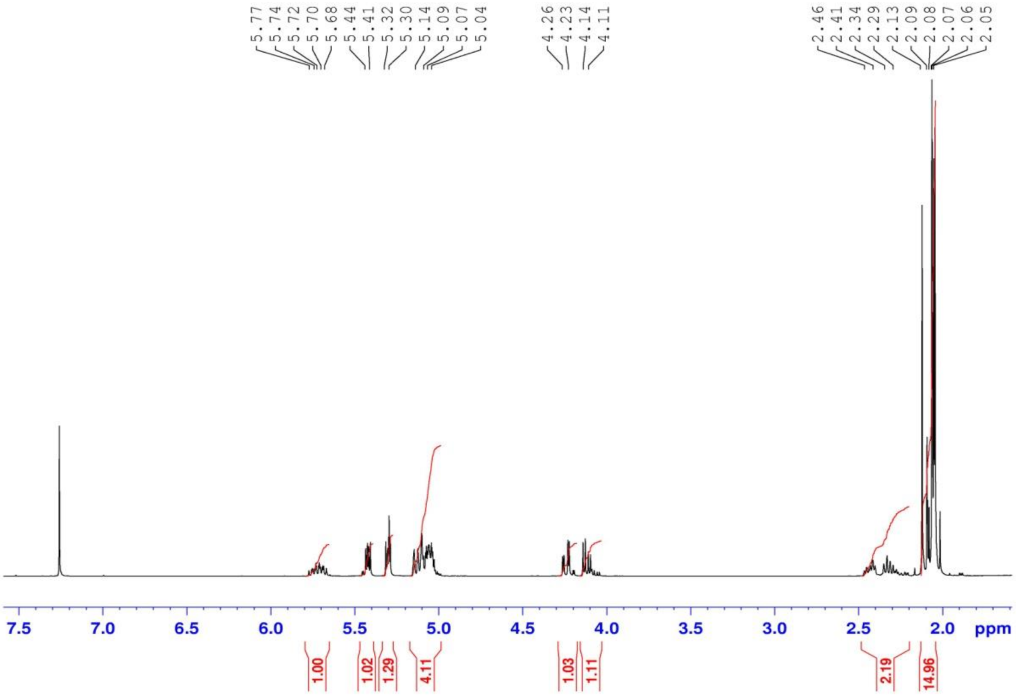

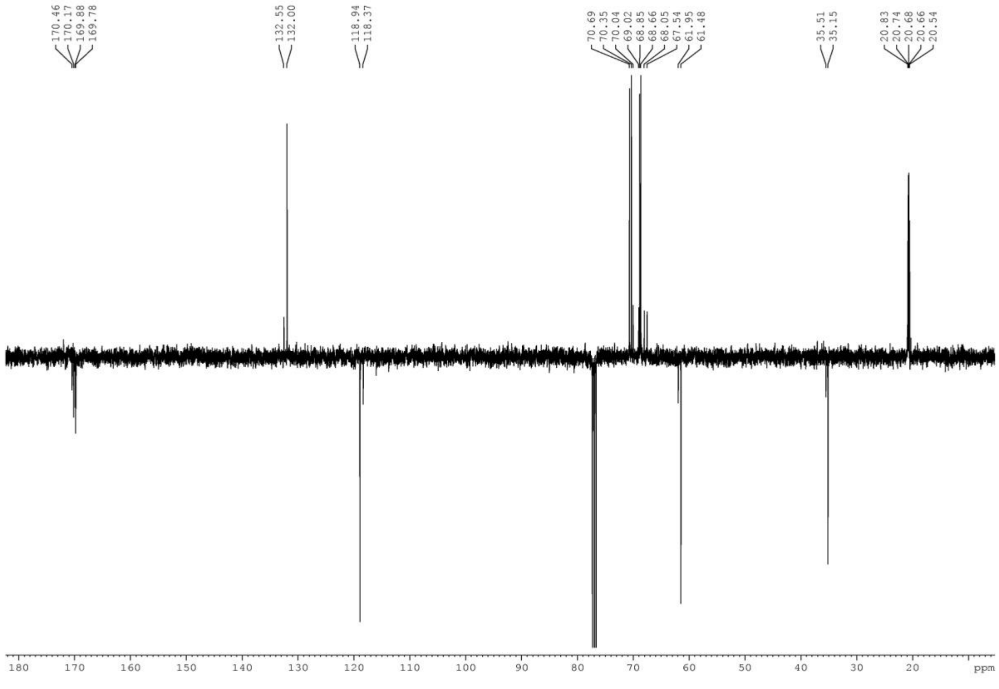

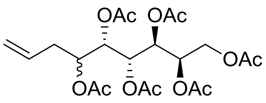

(2b)

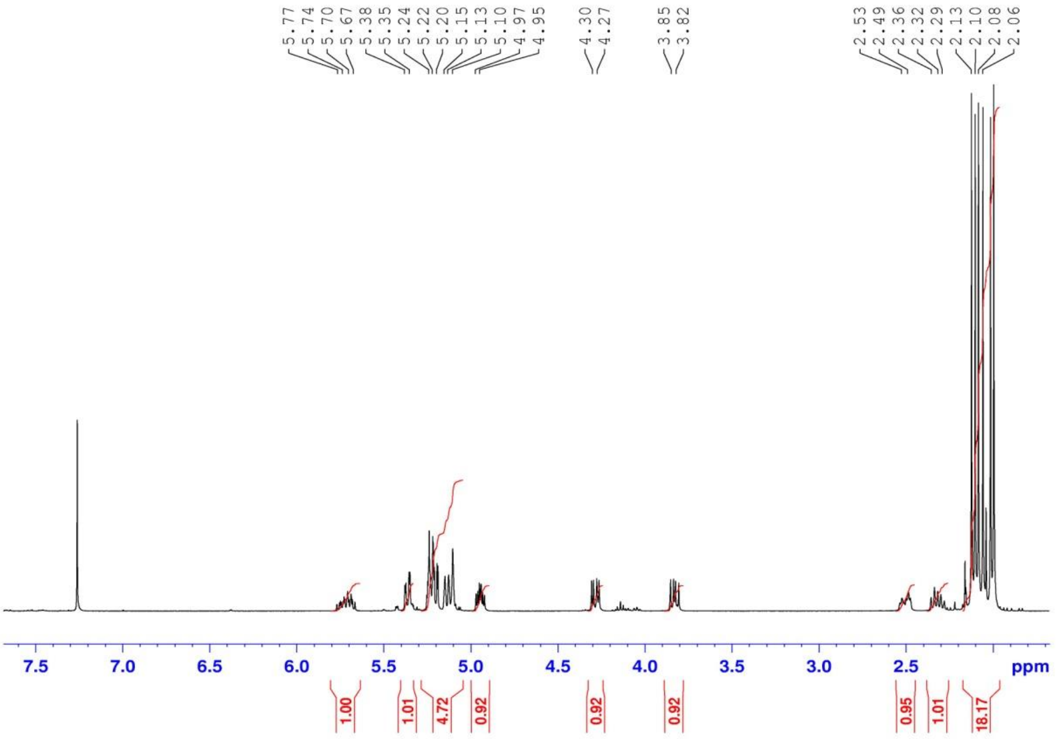

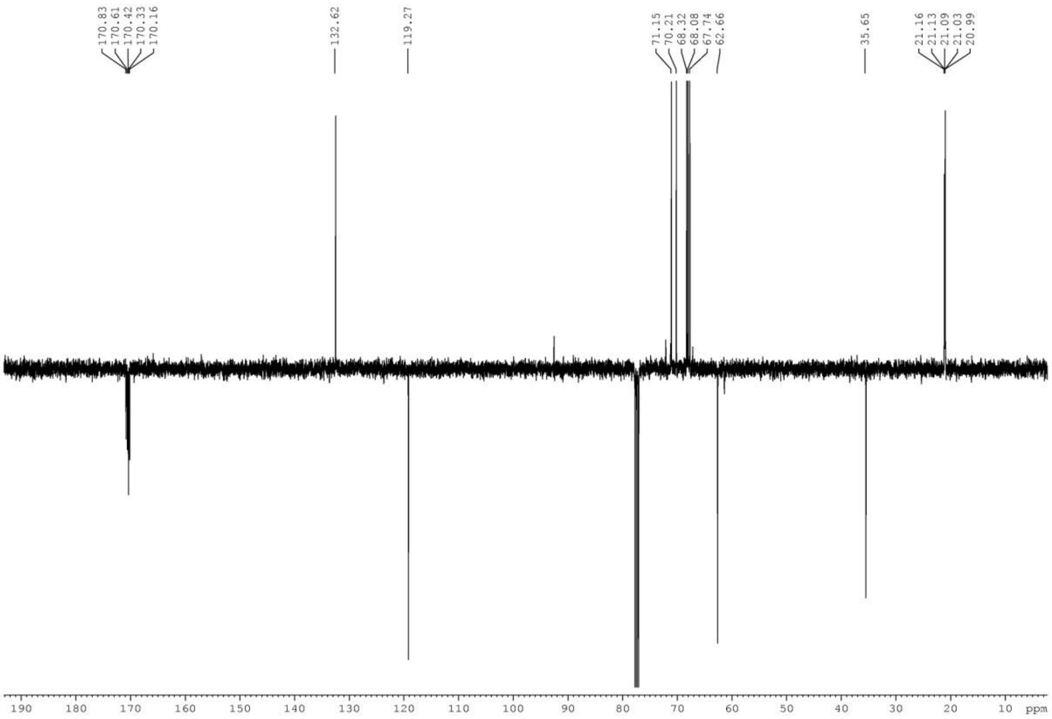

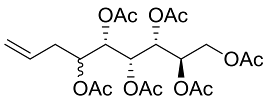
OAc OAc
OAc
OAc OAc OAc

(2c)

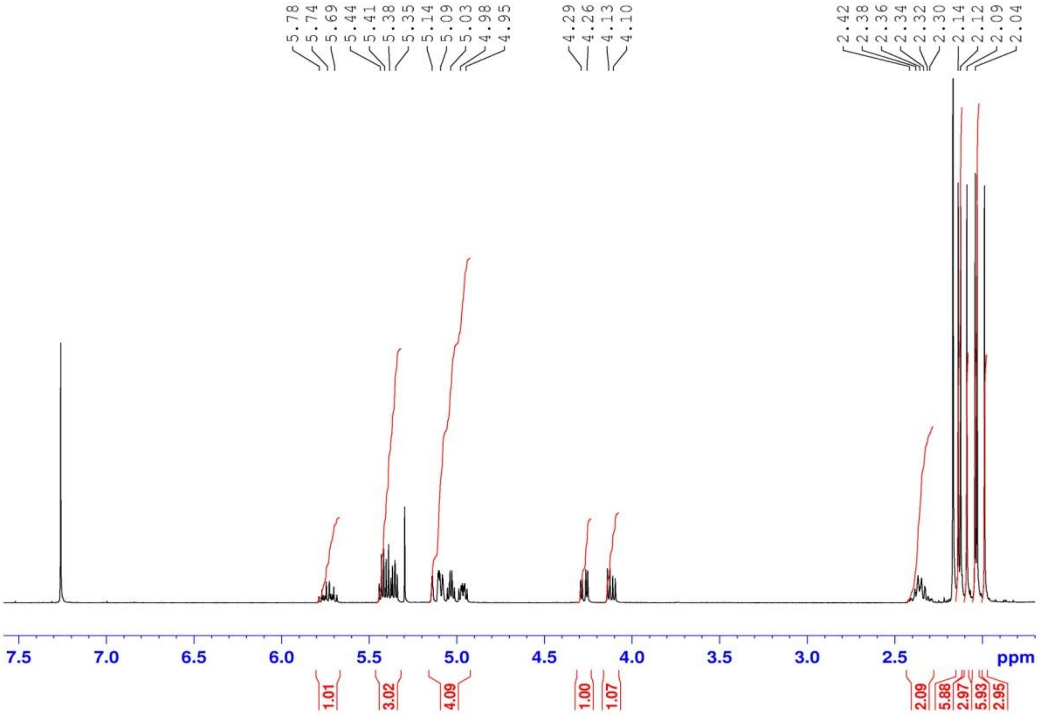
5.78 5.74 5.69 5.44 5.41 5.38 5.35 5.14 5.09 5.03 4.98 4.95
4.29 4.26 4.13 4.10
2.42 2.38 2.36 2.34 2.32 2.30 2.14 2.12 2.09 2.04
7.5 7.0 6.5 6.0 5.5 5.0 4.5 4.0 3.5 3.0 2.5 ppm
1.01 3.02 4.09 1.00 1.07 2.09 5.88 2.97 5.93 2.95

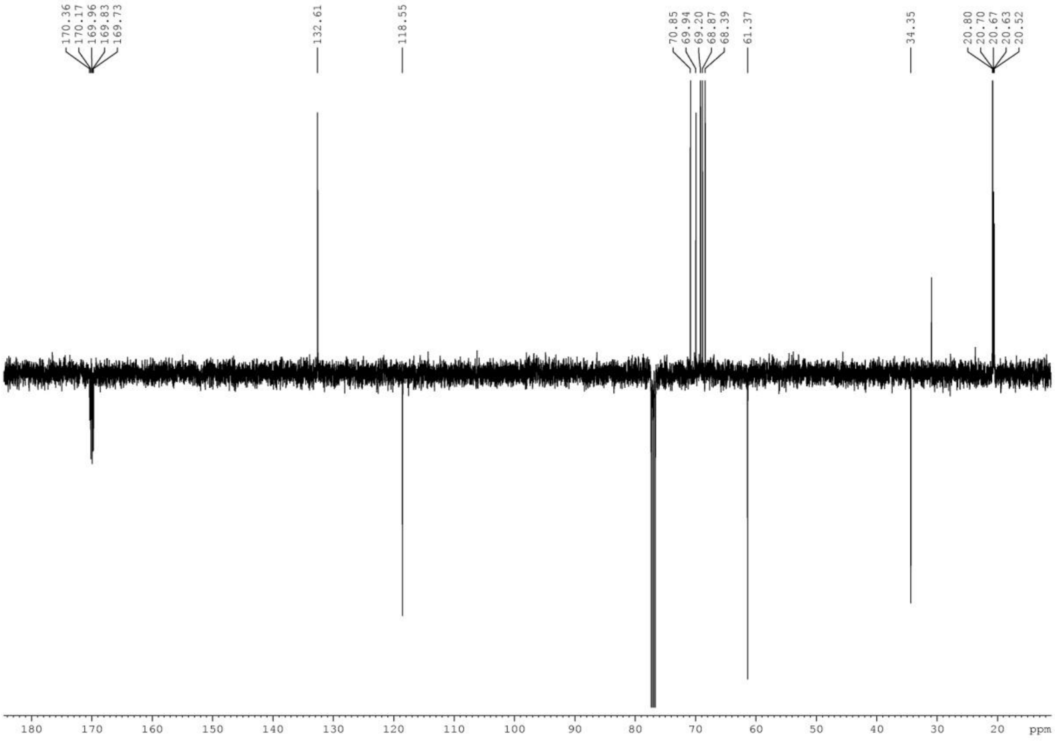
170.36 170.17 169.96 169.83 169.73
132.61
118.55
70.85 69.94 69.20 68.87 68.39
61.37
34.35
20.80 20.70 20.67 20.63 20.52
180 170 160 150 140 130 120 110 100 90 80 70 60 50 40 30 20 ppm

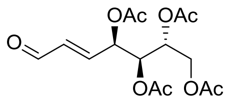
O
OAc OAc
OAc OAc

(3a)

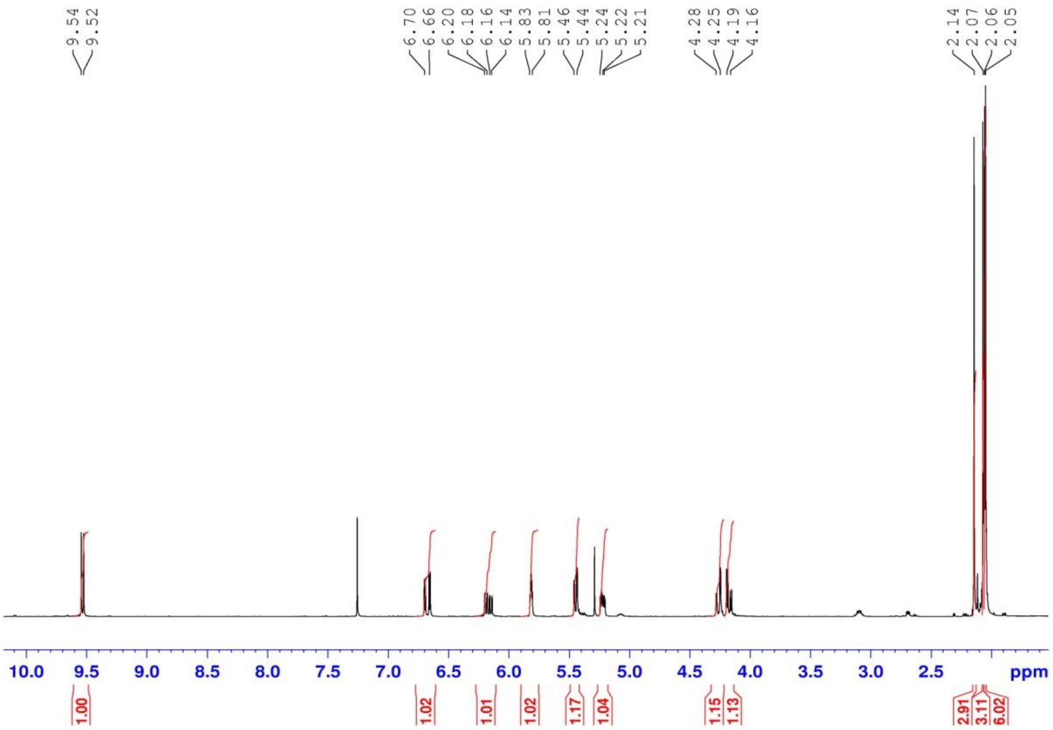
9.54
9.52
6.70
6.66
6.20
6.18
6.16
6.14
5.83
5.81
5.46
5.44
5.24
5.22
5.21
4.28
4.25
4.19
4.16
2.14
2.07
2.06
2.05
10.0 9.5 9.0 8.5 8.0 7.5 7.0 6.5 6.0 5.5 5.0 4.5 4.0 3.5 3.0 2.5 ppm
1.00
1.02
1.01
1.02
1.17
1.04
1.15
1.13
2.91
3.11
6.02

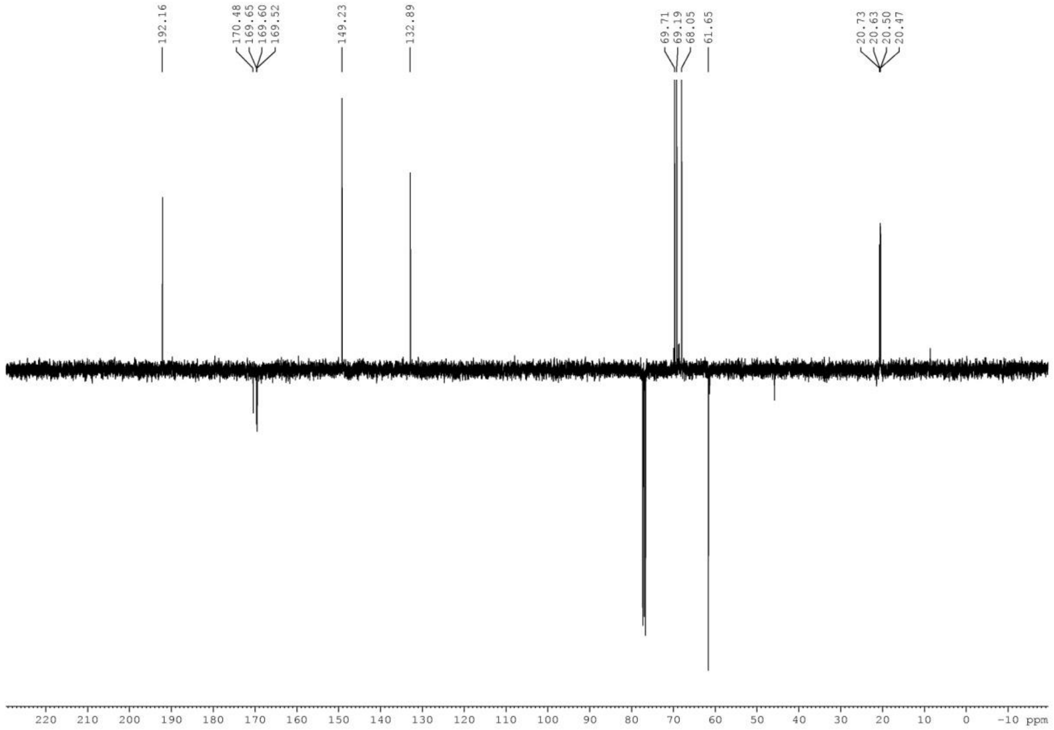
192.16
170.48
169.65
169.60
169.52
149.23
132.89
69.71
69.19
68.05
61.65
20.73
20.63
20.50
20.47
220 210 200 190 180 170 160 150 140 130 120 110 100 90 80 70 60 50 40 30 20 10 0 −10 ppm

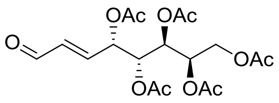
OAc OAc
O
OAc
OAc OAc

(3b)

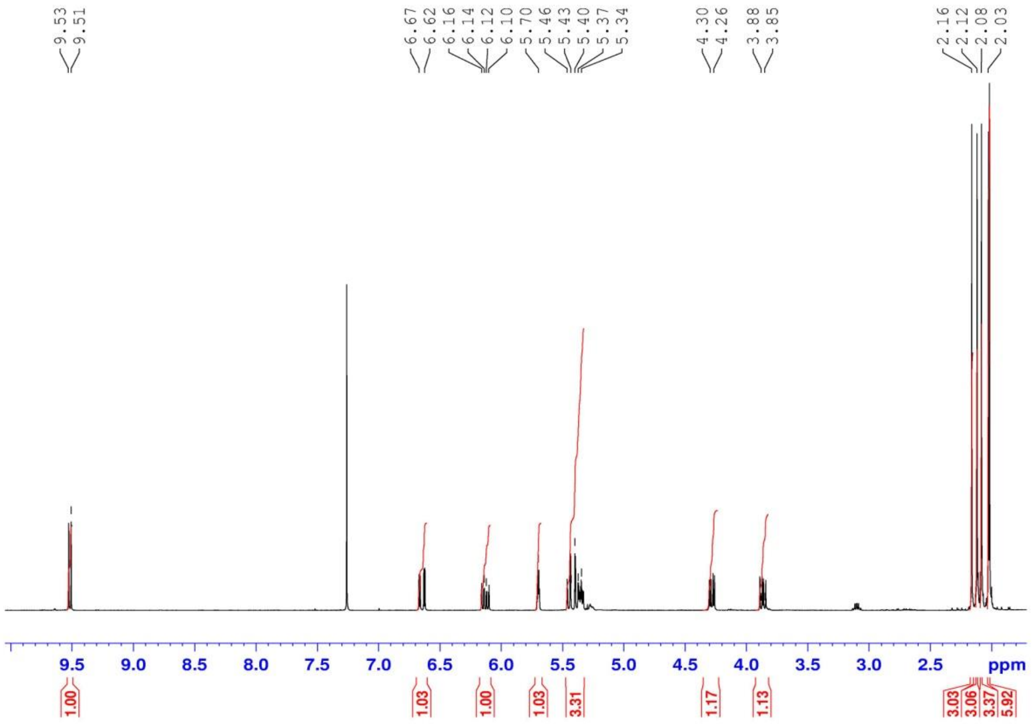
9.53
9.51
6.67
6.62
6.16
6.14
6.12
6.10
5.70
5.46
5.43
5.40
5.37
5.34
4.30
4.26
3.88
3.85
2.16
2.12
2.08
2.03
9.5 9.0 8.5 8.0 7.5 7.0 6.5 6.0 5.5 5.0 4.5 4.0 3.5 3.0 2.5 ppm
1.00
1.03
1.00
1.03
3.31
1.17
1.13
3.03
3.06
3.37
5.92

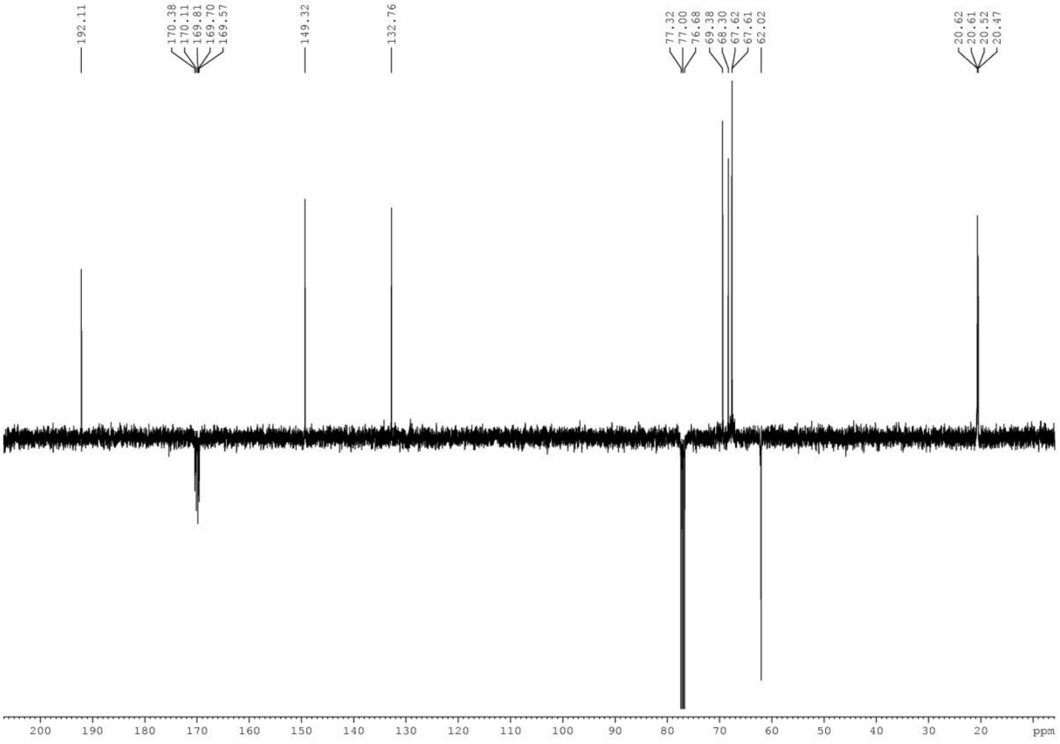
192.11
170.38
170.11
169.81
169.70
169.57
149.32
132.76
77.32
77.00
76.68
69.38
68.30
67.62
67.61
62.02
20.62
20.61
20.52
20.47
200 190 180 170 160 150 140 130 120 110 100 90 80 70 60 50 40 30 20 ppm

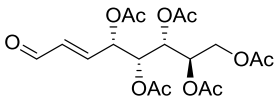
OAc
OAc
O
OAc
OAc
OAc

(3c)

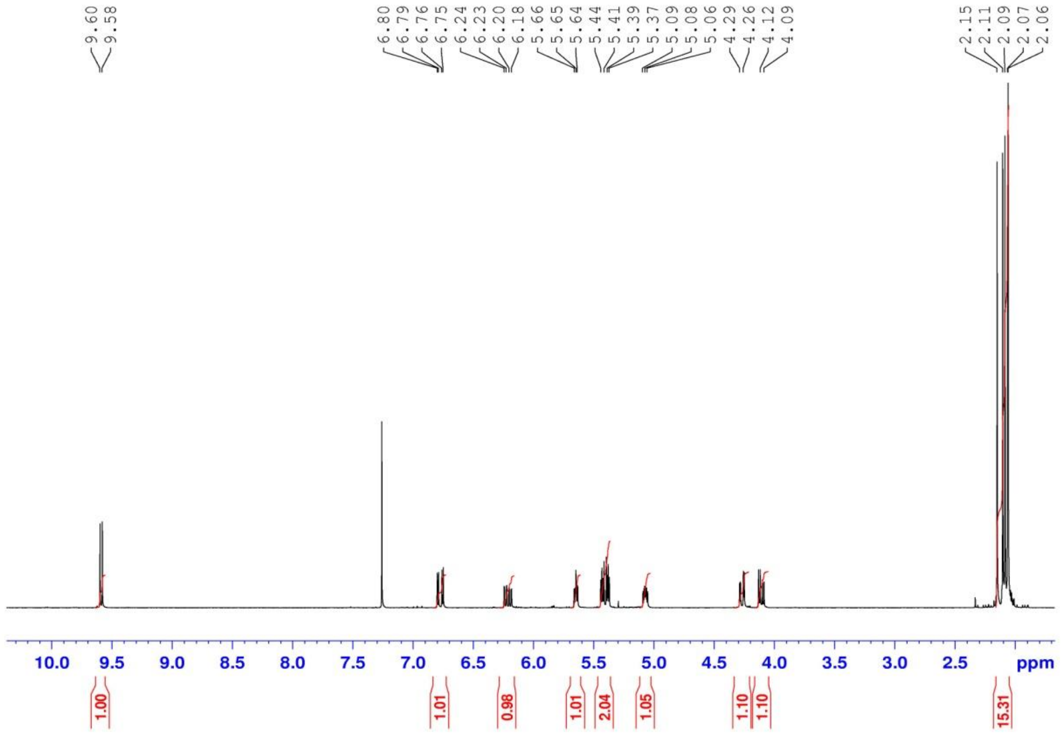
9.60
9.58
6.80
6.79
6.76
6.75
6.24
6.23
6.20
6.18
5.66
5.65
5.64
5.44
5.41
5.39
5.37
5.09
5.08
5.06
4.29
4.26
4.12
4.09
2.15
2.11
2.09
2.07
2.06
10.0
9.5
9.0
8.5
8.0
7.5
7.0
6.5
6.0
5.5
5.0
4.5
4.0
3.5
3.0
2.5
ppm
1.00
1.01
0.98
1.01
2.04
1.05
1.10
1.10
15.31

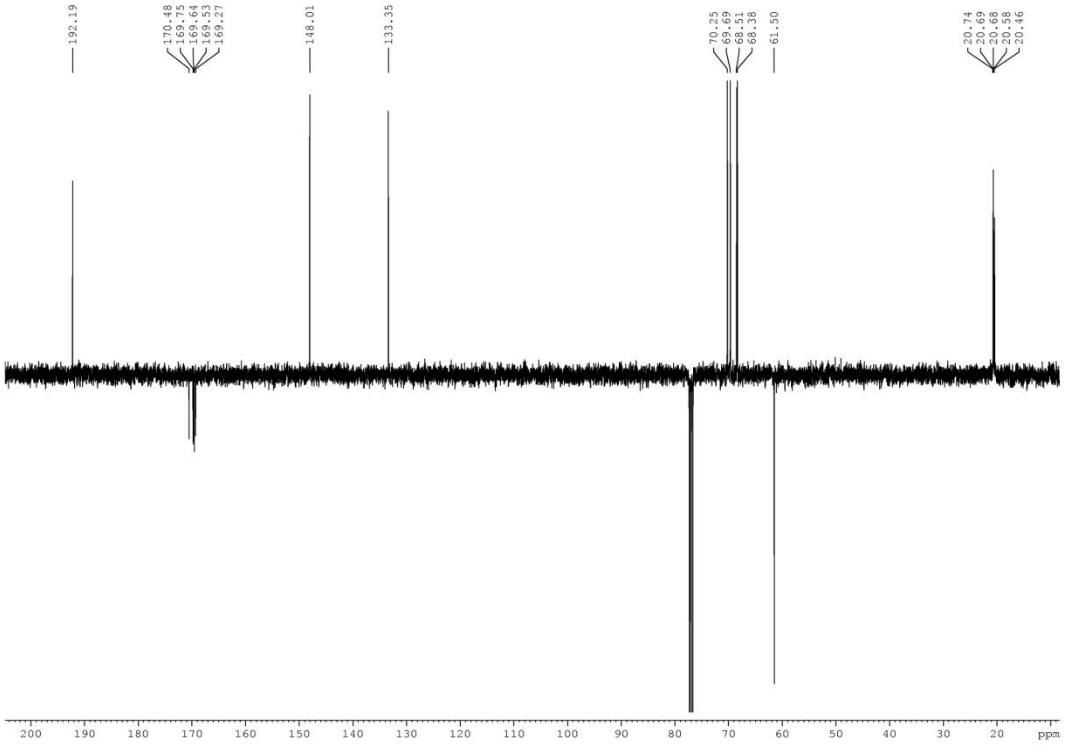
192.19
170.48
169.75
169.64
169.53
169.27
148.01
133.35
70.25
69.69
68.51
68.38
61.50
20.74
20.69
20.68
20.58
20.46
200
190
180
170
160
150
140
130
120
110
100
90
80
70
60
50
40
30
20
ppm

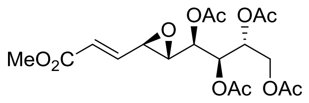
OAc
OAc
O
MeO2C
OAc
OAc

(4a)

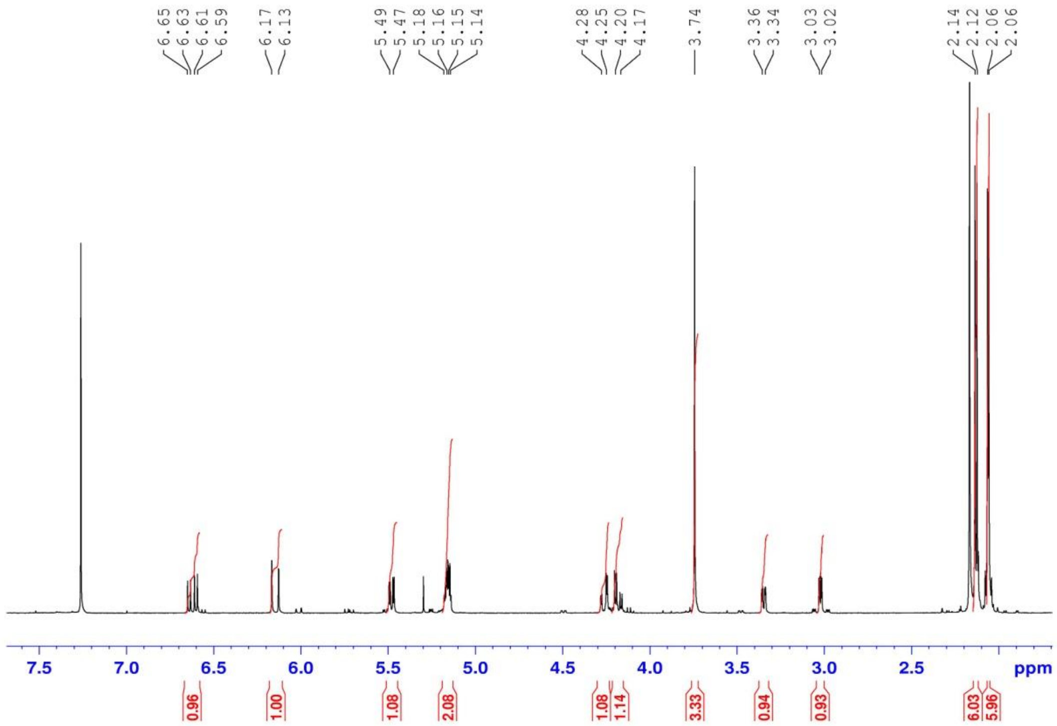
6.65
6.63
6.61
6.59
6.17
6.13
5.49
5.47
5.18
5.16
5.15
5.14
4.28
4.25
4.20
4.17
3.74
3.36
3.34
3.03
3.02
2.14
2.12
2.06
2.06
7.5
7.0
6.5
6.0
5.5
5.0
4.5
4.0
3.5
3.0
2.5
ppm
0.96
1.00
1.08
2.08
1.08
1.14
3.33
0.94
0.93
6.03
5.96

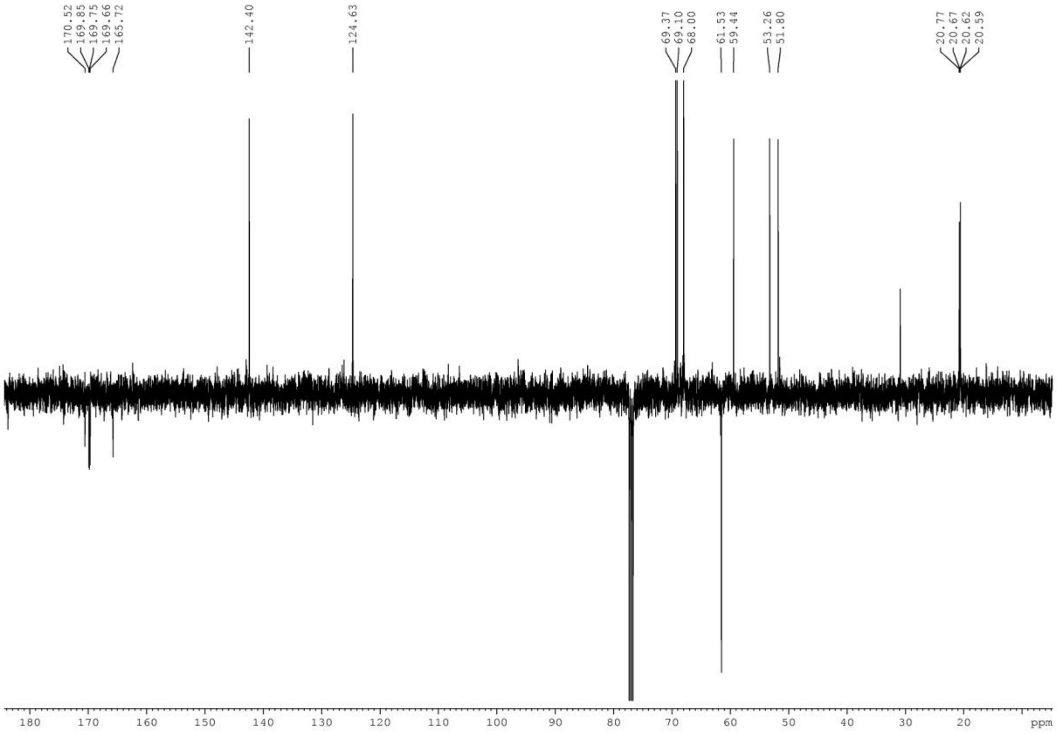
170.52
169.85
169.75
169.66
165.72
142.40
124.63
69.37
69.10
68.00
61.53
59.44
53.26
51.80
20.77
20.67
20.62
20.59
180
170
160
150
140
130
120
110
100
90
80
70
60
50
40
30
20
ppm

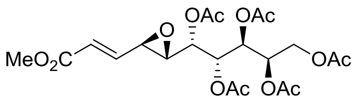

OAc OAc
O
MeO2C
OAc
OAc OAc

(4b)

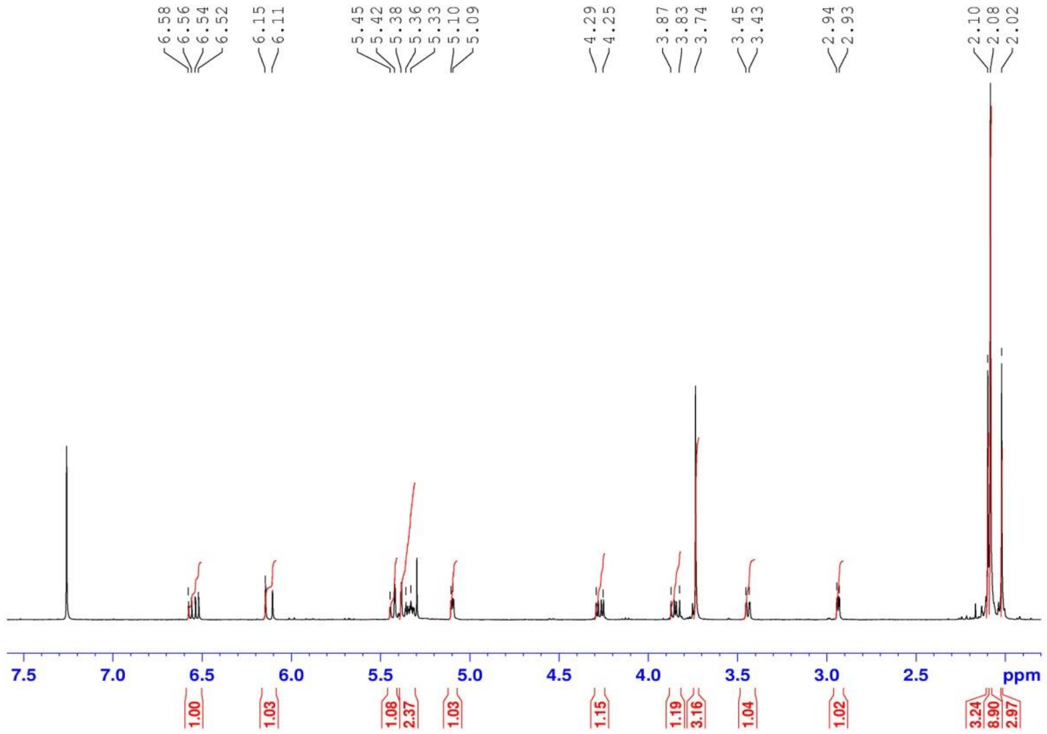

6.58 6.56 6.54 6.52 6.15 6.11 5.45 5.42 5.38 5.36 5.33 5.10 5.09 4.29 4.25 3.87 3.83 3.74 3.45 3.43 2.94 2.93 2.10 2.08 2.02
7.5 7.0 6.5 6.0 5.5 5.0 4.5 4.0 3.5 3.0 2.5 ppm
1.00 1.03 1.08 2.37 1.03 1.15 1.19 3.16 1.04 1.02 3.24 8.90 2.97

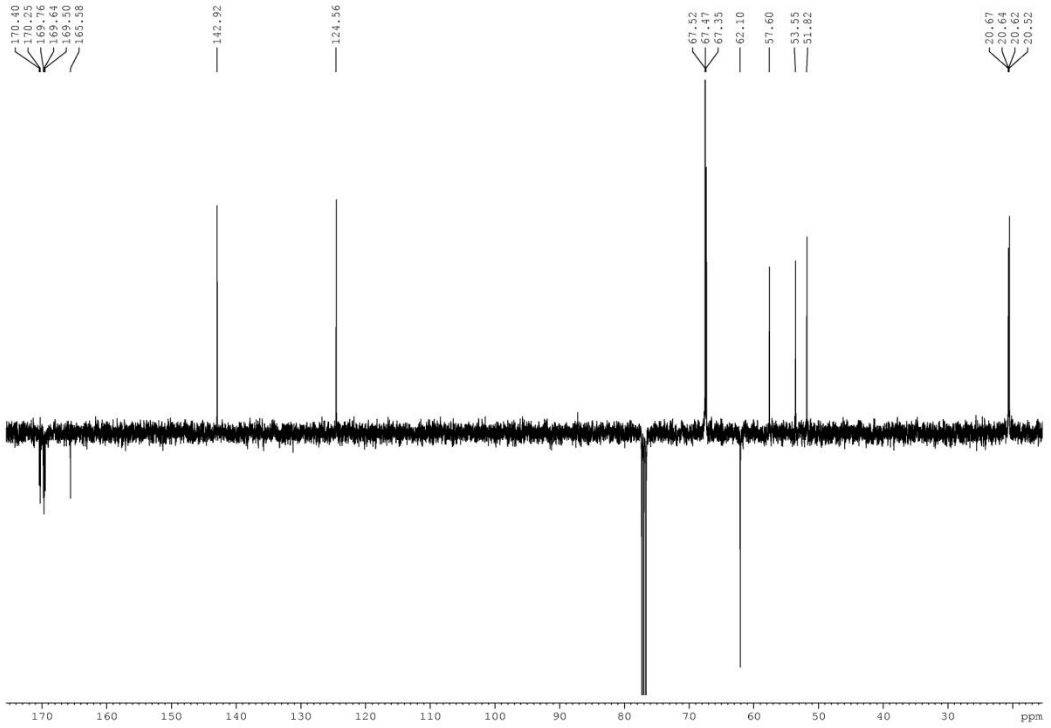

170.40 170.25 169.76 169.64 169.50 165.58 142.92 124.56 67.52 67.47 67.35 62.10 57.60 53.55 51.82 20.67 20.64 20.62 20.52
170 160 150 140 130 120 110 100 90 80 70 60 50 40 30 ppm

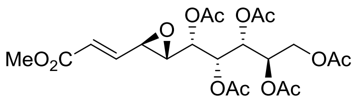
OAc OAc
O
MeO2C
OAc
OAc OAc

(4c)

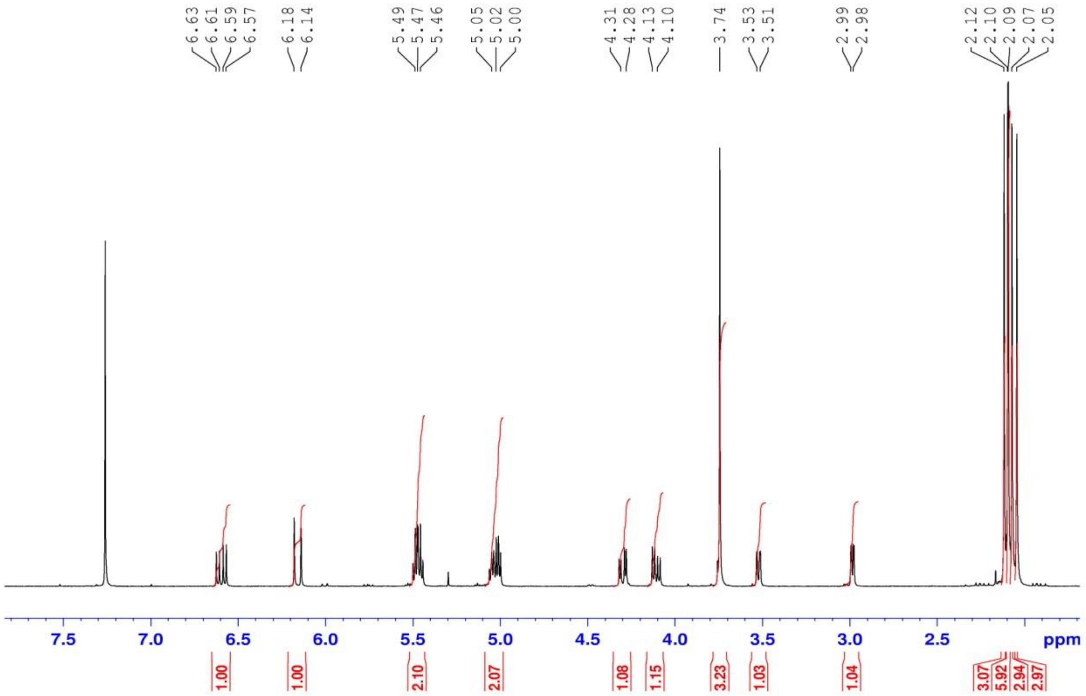
6.63
6.61
6.59
6.57
6.18
6.14
5.49
5.47
5.46
5.05
5.02
5.00
4.31
4.28
4.13
4.10
3.74
3.53
3.51
2.99
2.98
2.12
2.10
2.09
2.07
2.05
7.5
7.0
6.5
6.0
5.5
5.0
4.5
4.0
3.5
3.0
2.5
ppm
1.00
1.00
2.10
2.07
1.08
1.15
3.23
1.03
1.04
3.07
5.92
2.94
2.97

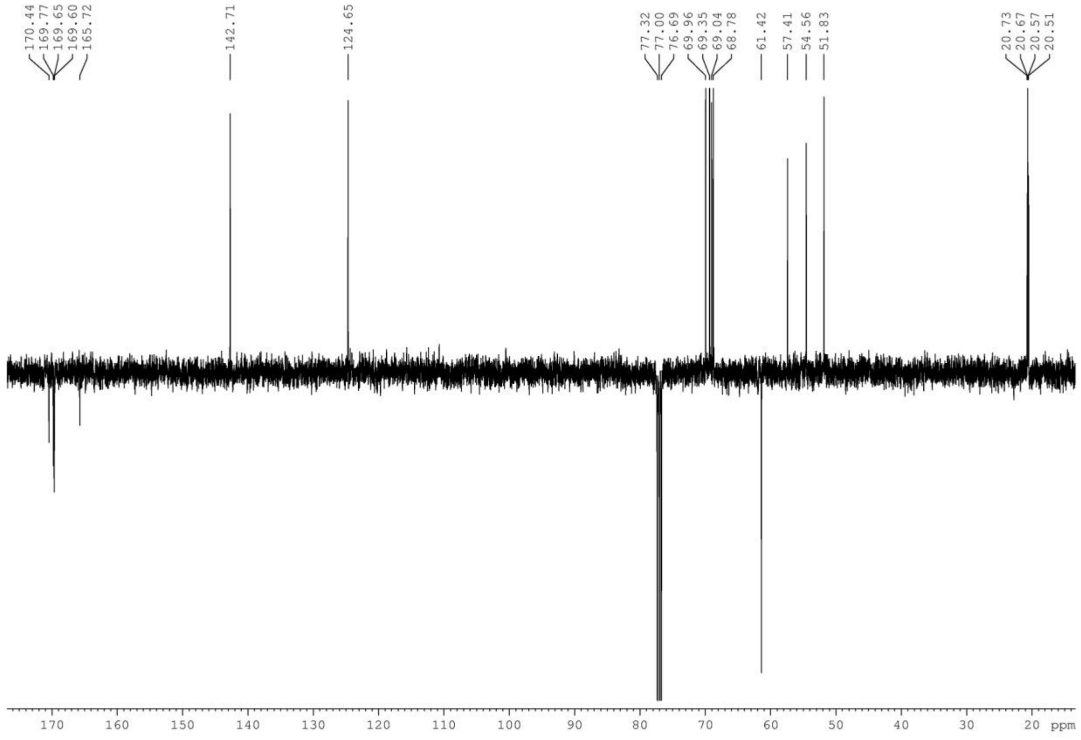
170.44
169.77
169.65
169.60
165.72
142.71
124.65
77.32
77.00
76.69
69.96
69.35
69.04
68.78
61.42
57.41
54.56
51.83
20.73
20.67
20.57
20.51
170
160
150
140
130
120
110
100
90
80
70
60
50
40
30
20 ppm

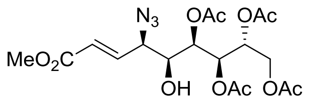
N3
OAc
OAc
MeO2C
OH
OAc
OAc

(5a)

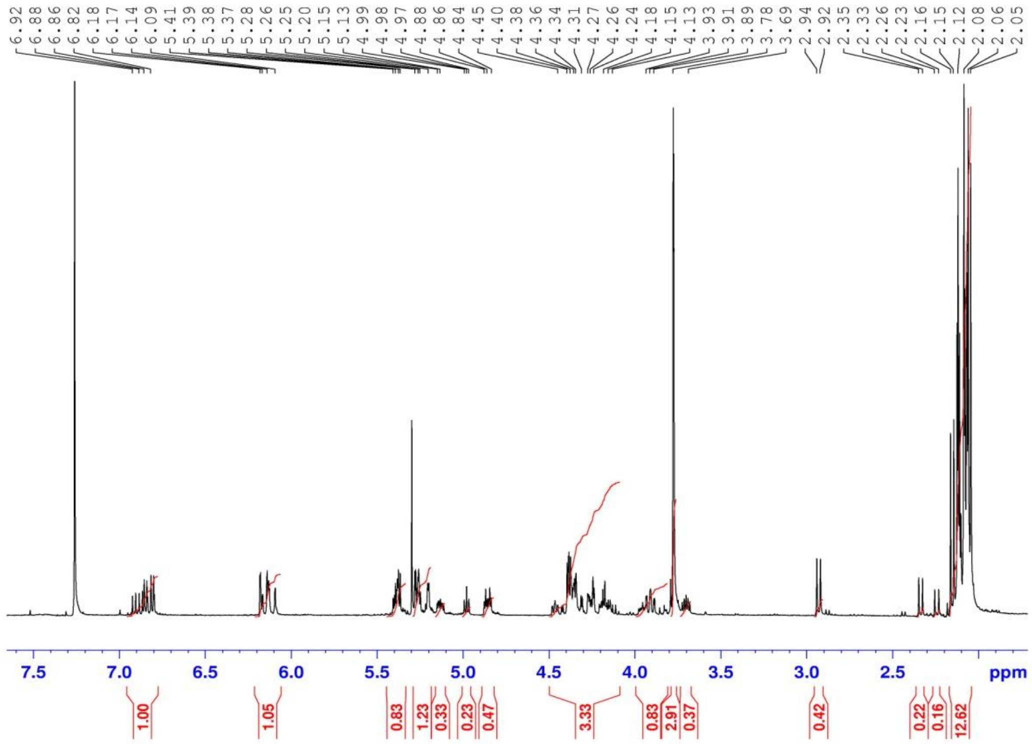
6.92
6.88
6.86
6.82
6.18
6.17
6.14
6.09
5.41
5.39
5.38
5.37
5.28
5.26
5.25
5.20
5.15
5.13
4.99
4.98
4.97
4.88
4.86
4.84
4.45
4.40
4.38
4.36
4.34
4.31
4.27
4.26
4.24
4.18
4.15
4.13
3.93
3.91
3.89
3.78
3.69
2.94
2.92
2.35
2.33
2.26
2.23
2.16
2.15
2.12
2.08
2.06
2.05
7.5
7.0
6.5
6.0
5.5
5.0
4.5
4.0
3.5
3.0
2.5
ppm
1.00
1.05
0.83
1.23
0.33
0.23
0.47
3.33
0.83
2.91
0.37
0.42
0.22
0.16
12.62

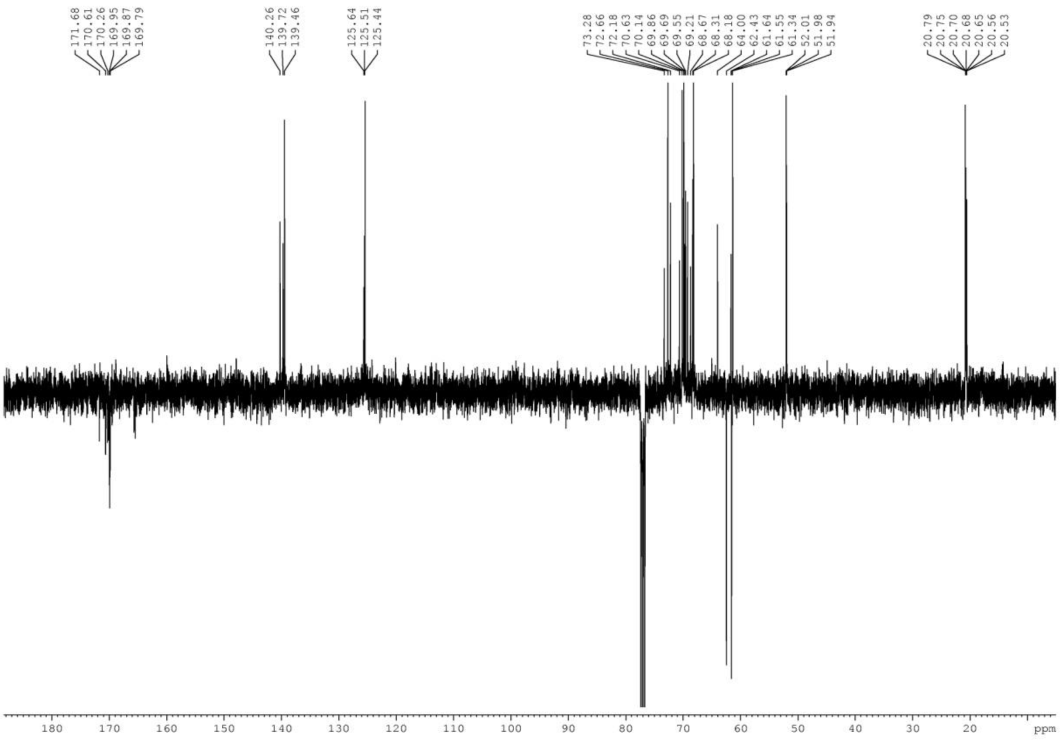
171.68
170.61
170.26
169.95
169.87
169.79
140.26
139.72
139.46
125.64
125.51
125.44
73.28
72.66
72.18
70.63
70.14
69.86
69.69
69.55
69.21
68.67
68.31
68.18
64.00
62.43
61.64
61.55
61.34
52.01
51.98
51.94
20.79
20.75
20.70
20.68
20.65
20.56
20.53
180
170
160
150
140
130
120
110
100
90
80
70
60
50
40
30
20
ppm

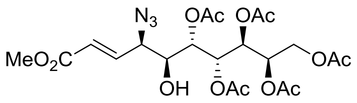
N3
OAc OAc
MeO2C
OAc
OH
OAc OAc

(5b)

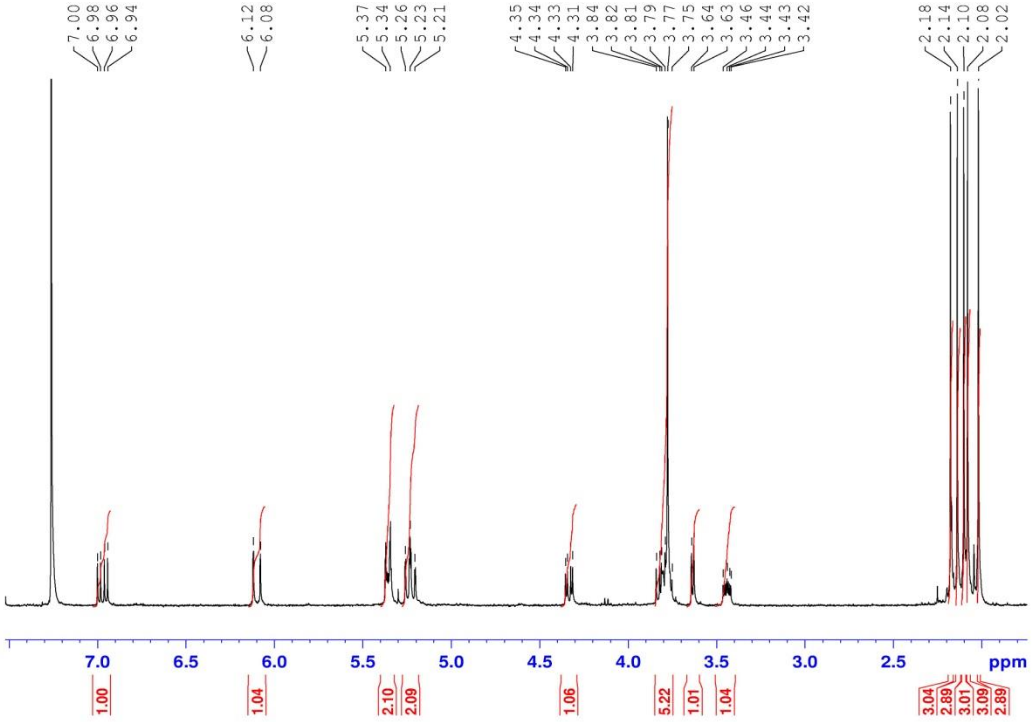
7.00
6.98
6.96
6.94
6.12
6.08
5.37
5.34
5.26
5.23
5.21
4.35
4.34
4.33
4.31
3.84
3.82
3.81
3.79
3.77
3.75
3.64
3.63
3.46
3.44
3.43
3.42
2.18
2.14
2.10
2.08
2.02
7.0
6.5
6.0
5.5
5.0
4.5
4.0
3.5
3.0
2.5
ppm
1.00
1.04
2.10
2.09
1.06
5.22
1.01
1.04
3.04
2.89
3.01
3.09
2.89

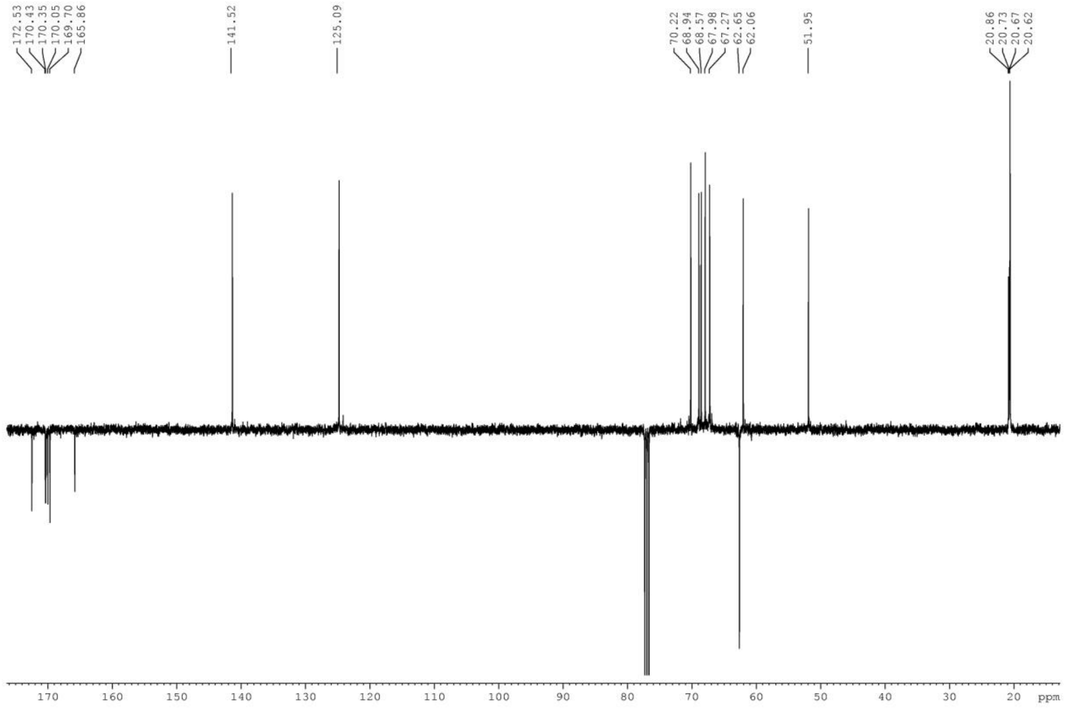
172.53
170.43
170.35
170.05
169.70
165.86
141.52
125.09
70.22
68.94
68.57
67.98
67.27
62.65
62.06
51.95
20.86
20.73
20.67
20.62
170
160
150
140
130
120
110
100
90
80
70
60
50
40
30
20
ppm

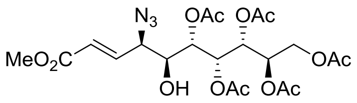
N3
OAc
OAc
MeO2C
OAc
OH
OAc
OAc

(5c)

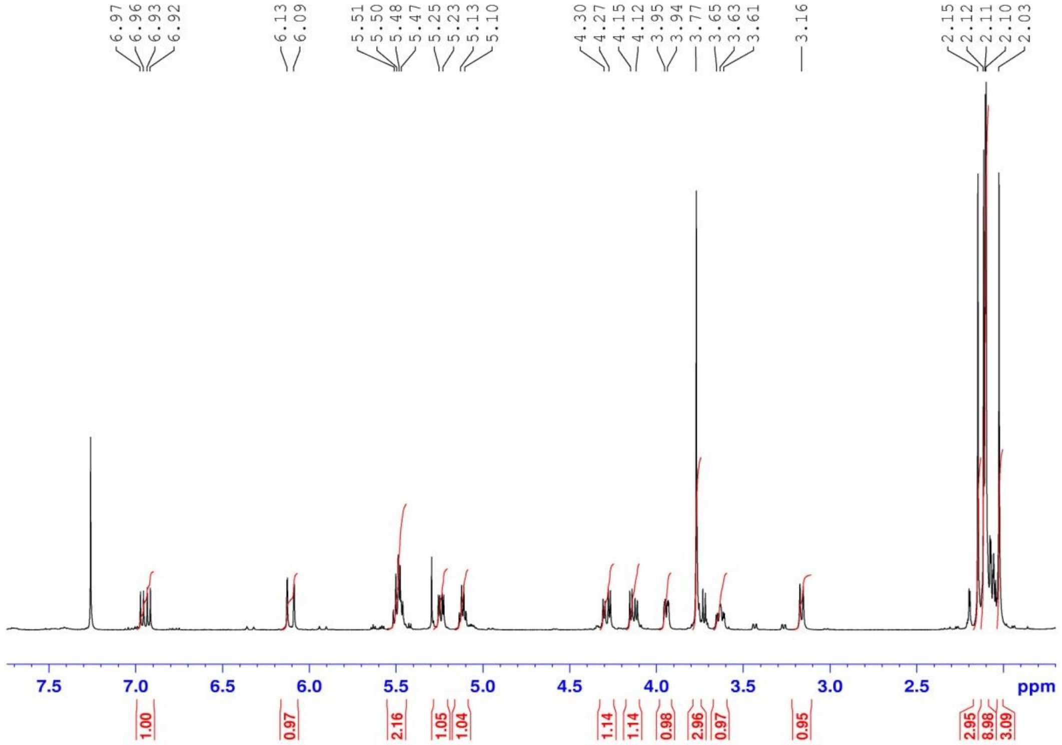
6.97
6.96
6.93
6.92
6.13
6.09
5.51
5.50
5.48
5.47
5.25
5.23
5.13
5.10
4.30
4.27
4.15
4.12
3.95
3.94
3.77
3.65
3.63
3.61
3.16
2.15
2.12
2.11
2.10
2.03
7.5
7.0
6.5
6.0
5.5
5.0
4.5
4.0
3.5
3.0
2.5
ppm
1.00
0.97
2.16
1.05
1.04
1.14
1.14
0.98
2.96
0.97
0.95
2.95
8.98
3.09

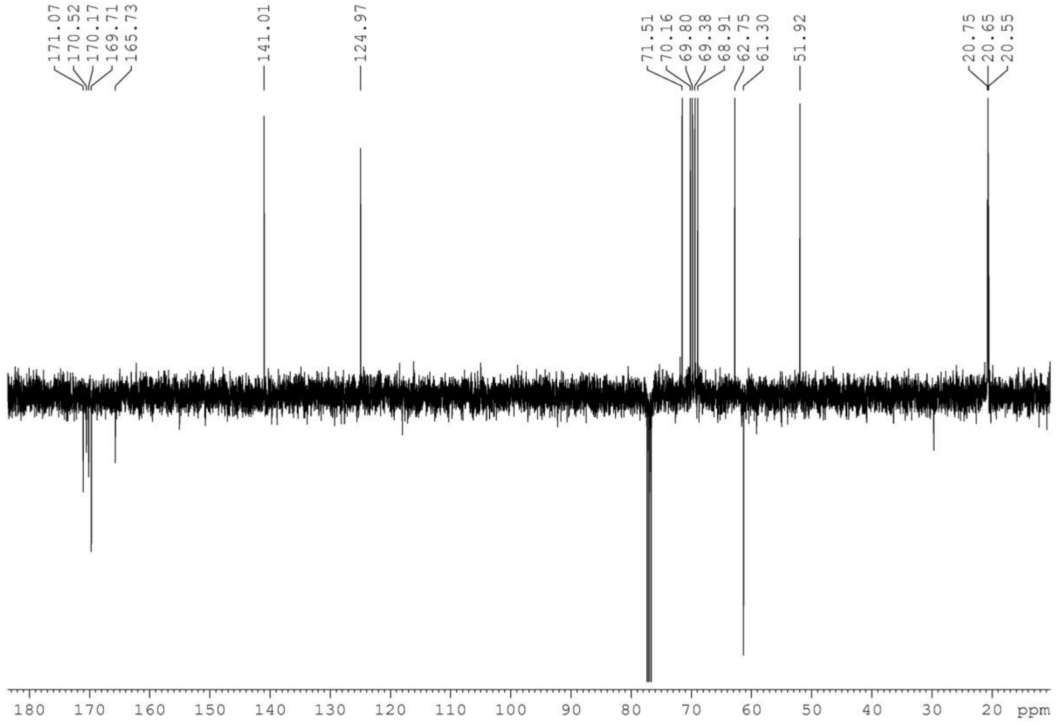
171.07
170.52
170.17
169.71
165.73
141.01
124.97
71.51
70.16
69.80
69.38
68.91
62.75
61.30
51.92
20.75
20.65
20.55
180
170
160
150
140
130
120
110
100
90
80
70
60
50
40
30
20
ppm

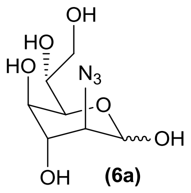
OH
HO
HO
N3
O
OH
OH

(6a)

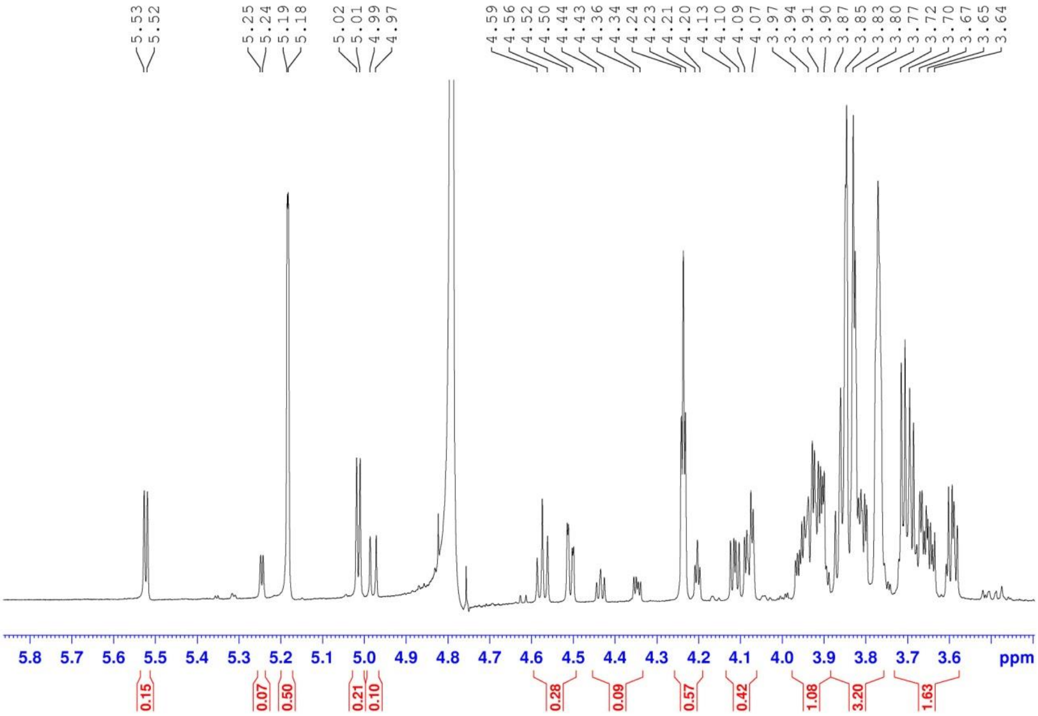
5.53
5.52
5.25
5.24
5.19
5.18
5.02
5.01
4.99
4.97
4.59
4.56
4.52
4.50
4.44
4.43
4.36
4.34
4.24
4.23
4.21
4.20
4.13
4.10
4.09
4.07
3.97
3.94
3.91
3.90
3.87
3.85
3.83
3.80
3.77
3.72
3.70
3.67
3.65
3.64
5.8 5.7 5.6 5.5 5.4 5.3 5.2 5.1 5.0 4.9 4.8 4.7 4.6 4.5 4.4 4.3 4.2 4.1 4.0 3.9 3.8 3.7 3.6 ppm
0.15
0.07
0.50
0.21
0.10
0.28
0.09
0.57
0.42
1.08
3.20
1.63

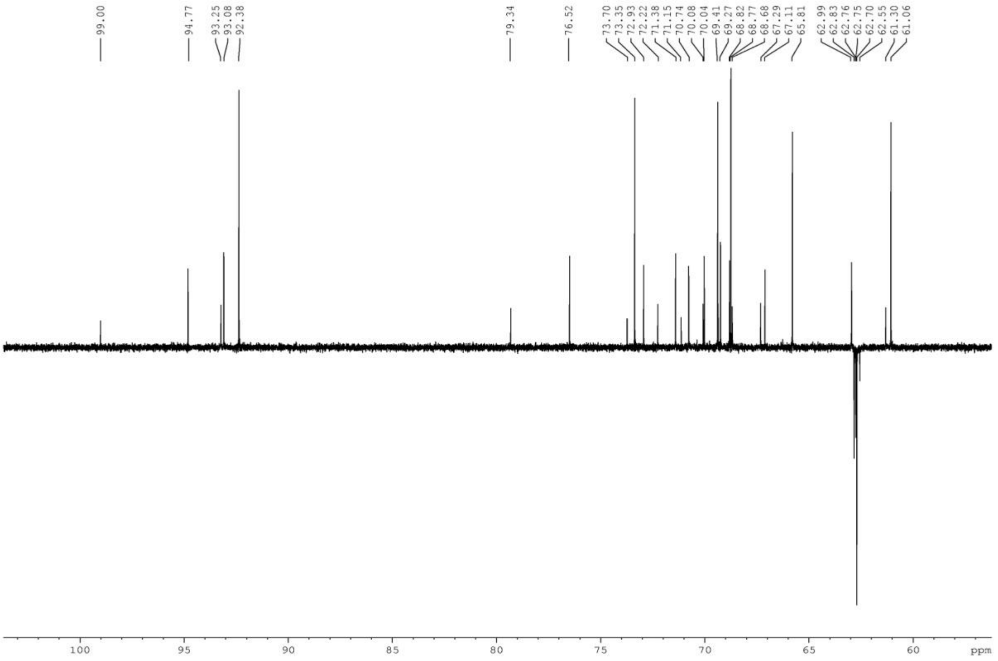
99.00
94.77
93.25
93.08
92.38
79.34
76.52
73.70
73.35
72.93
72.22
71.38
71.15
70.74
70.08
70.04
69.41
69.27
68.82
68.77
68.68
67.29
67.11
65.81
62.99
62.83
62.76
62.75
62.70
62.55
61.30
61.06
100 95 90 85 80 75 70 65 60 ppm

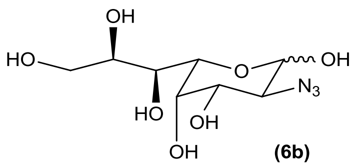

(6b)

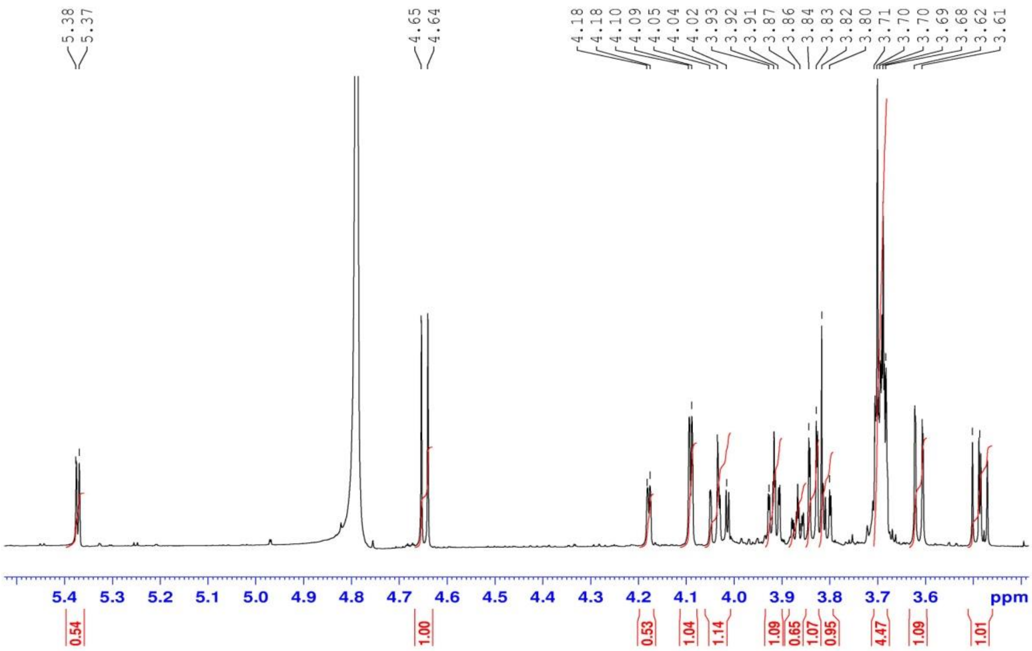

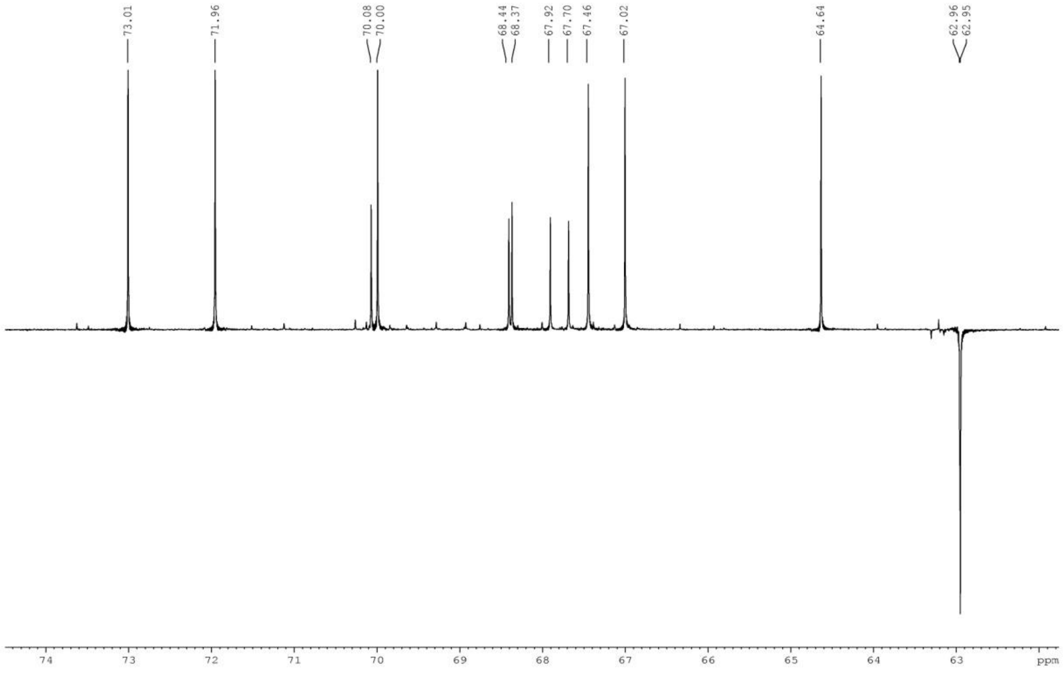

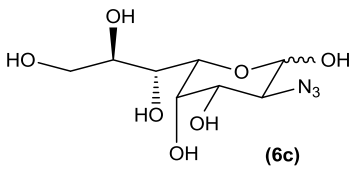
OH
HO
OH
N3
HO
OH
OH

(6c)

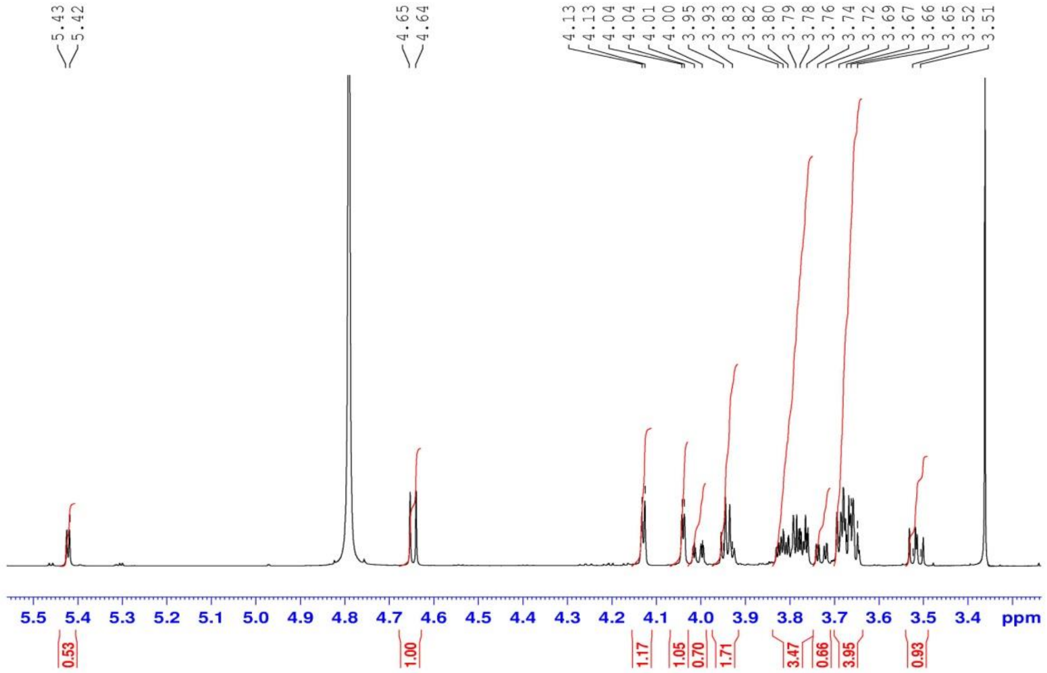
5.43
5.42
4.65
4.64
4.13
4.13
4.04
4.04
4.01
4.00
3.95
3.93
3.83
3.82
3.80
3.79
3.78
3.76
3.74
3.72
3.69
3.67
3.66
3.65
3.52
3.51
5.5 5.4 5.3 5.2 5.1 5.0 4.9 4.8 4.7 4.6 4.5 4.4 4.3 4.2 4.1 4.0 3.9 3.8 3.7 3.6 3.5 3.4 ppm
0.53
1.00
1.17
1.05
0.70
1.71
3.47
0.66
3.95
0.93

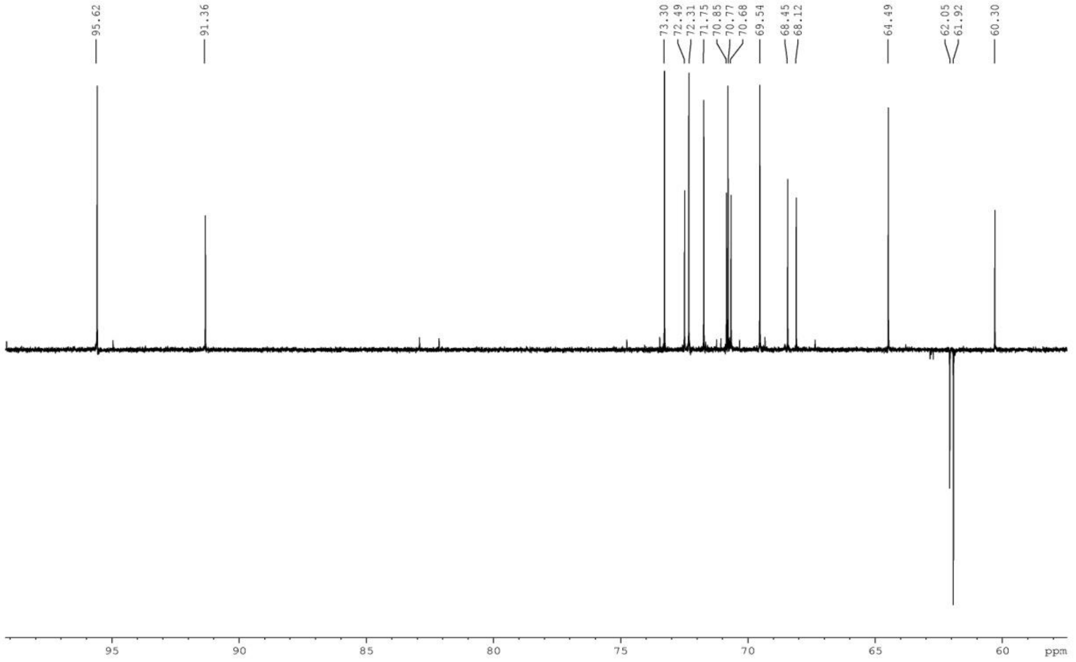
95.62
91.36
73.30
72.49
72.31
71.75
70.85
70.77
70.68
69.54
68.45
68.12
64.49
62.05
61.92
60.30
95 90 85 80 75 70 65 60 ppm

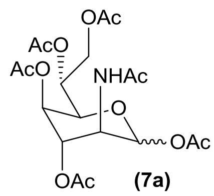

OAc
AcO
AcO
NHAc
O
OAc
OAc

(7a)

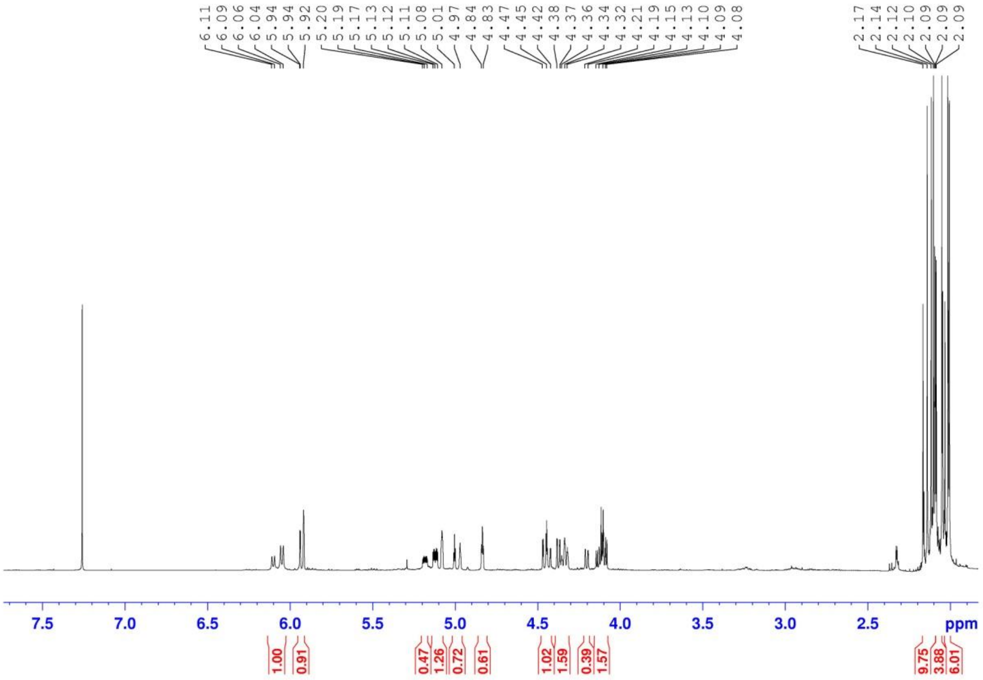

6.11
6.09
6.06
6.04
5.94
5.94
5.92
5.20
5.19
5.17
5.13
5.12
5.11
5.08
5.01
4.97
4.84
4.83
4.47
4.45
4.42
4.38
4.37
4.36
4.34
4.32
4.21
4.19
4.15
4.13
4.10
4.09
4.08
2.17
2.14
2.12
2.10
2.09
2.09
2.09
7.5
7.0
6.5
6.0
5.5
5.0
4.5
4.0
3.5
3.0
2.5
ppm
1.00
0.91
0.47
1.26
0.72
0.61
1.02
1.59
0.39
1.57
9.75
3.88
6.01

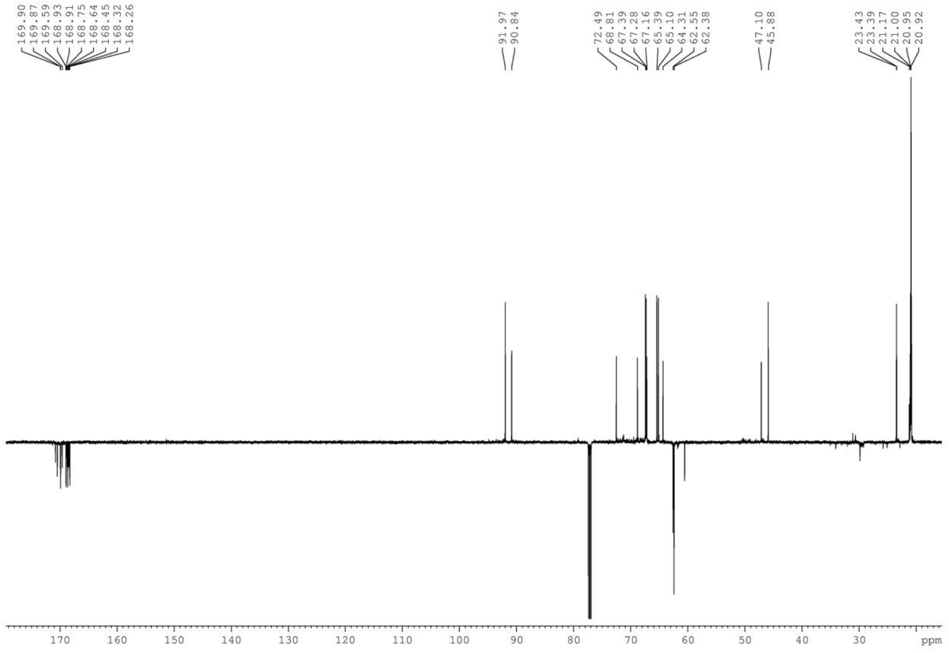

169.90
169.87
169.59
168.93
168.91
168.75
168.64
168.45
168.32
168.26
91.97
90.84
72.49
68.81
67.39
67.28
67.16
65.39
65.10
64.31
62.55
62.38
47.10
45.88
23.43
23.39
21.17
21.00
20.95
20.92
170
160
150
140
130
120
110
100
90
80
70
60
50
40
30
ppm

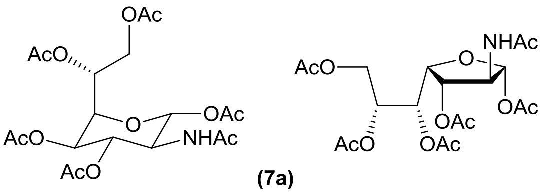
OAc
AcO
OAc
AcO
O
NHAc
AcO
NHAc
AcO
O
OAc
OAc
AcO
OAc

(7a)

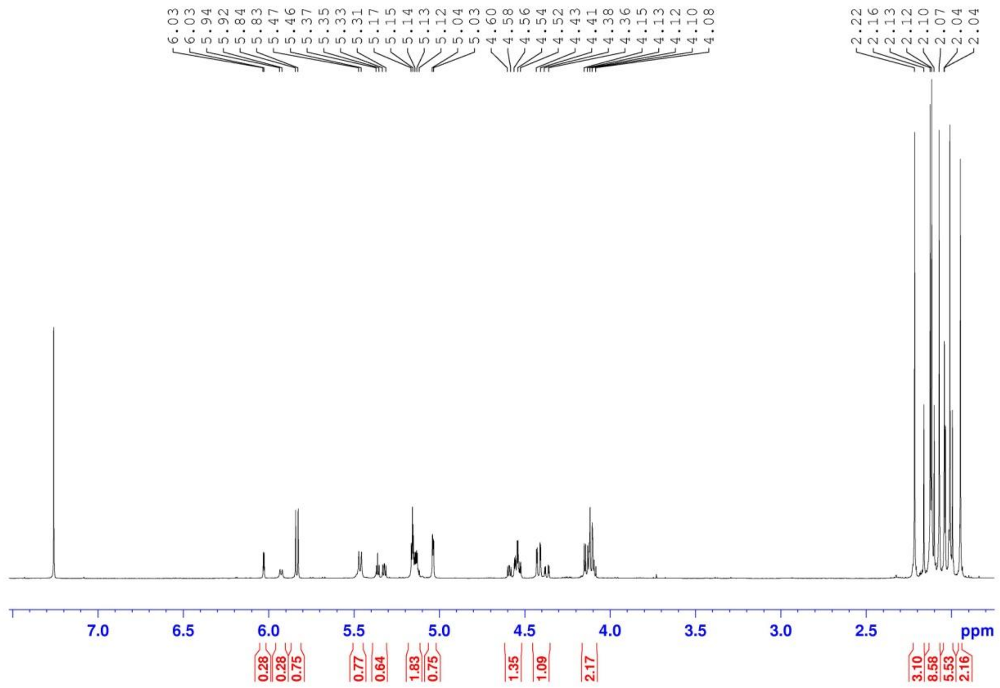
6.03 6.03 5.94 5.92 5.84 5.83 5.47 5.46 5.37 5.35 5.33 5.31 5.17 5.15 5.14 5.13 5.12 5.04 5.03 4.60 4.58 4.56 4.54 4.52 4.43 4.41 4.38 4.36 4.15 4.13 4.12 4.10 4.08
2.22 2.16 2.13 2.12 2.10 2.07 2.04 2.04
7.0 6.5 6.0 5.5 5.0 4.5 4.0 3.5 3.0 2.5 ppm
0.28 0.28 0.75 0.77 0.64 1.83 0.75 1.35 1.09 2.17 3.10 8.58 5.53 2.16

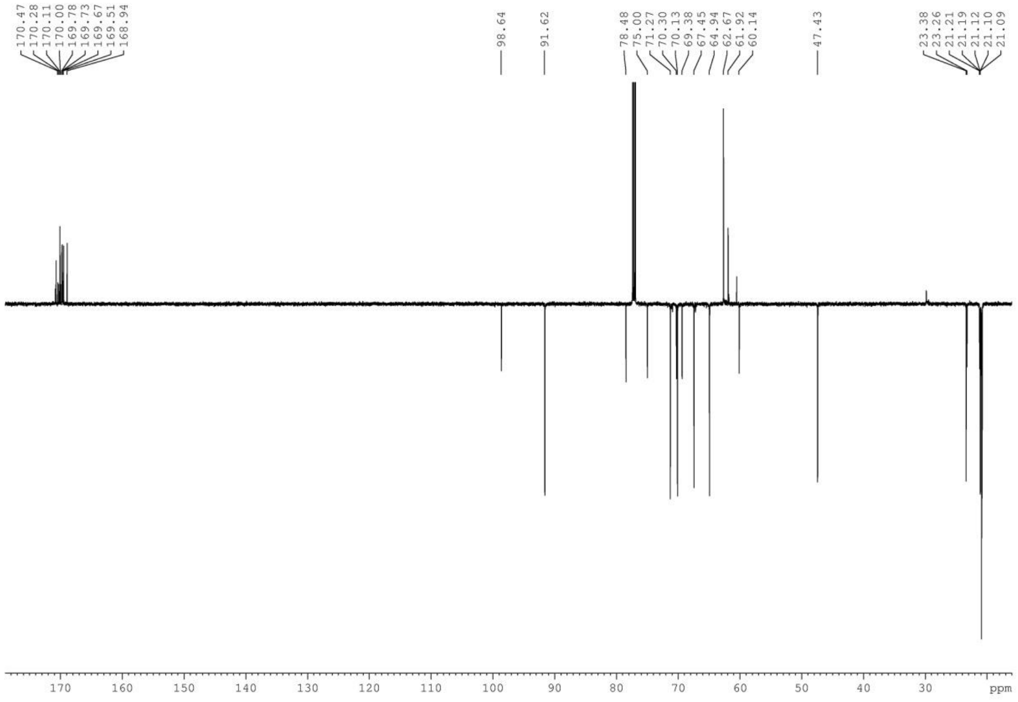
170.47 170.28 170.11 170.00 169.78 169.73 169.67 169.51 168.94
98.64
91.62
78.48 75.00 71.27 70.30 70.13 69.38 67.45 64.94 62.67 61.92 60.14
47.43
23.38 23.26 21.21 21.19 21.12 21.10 21.09
170 160 150 140 130 120 110 100 90 80 70 60 50 40 30 ppm

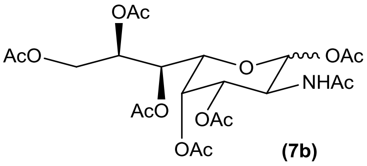
OAc
AcO
OAc
NHAc
AcO
OAc
OAc

(7b)

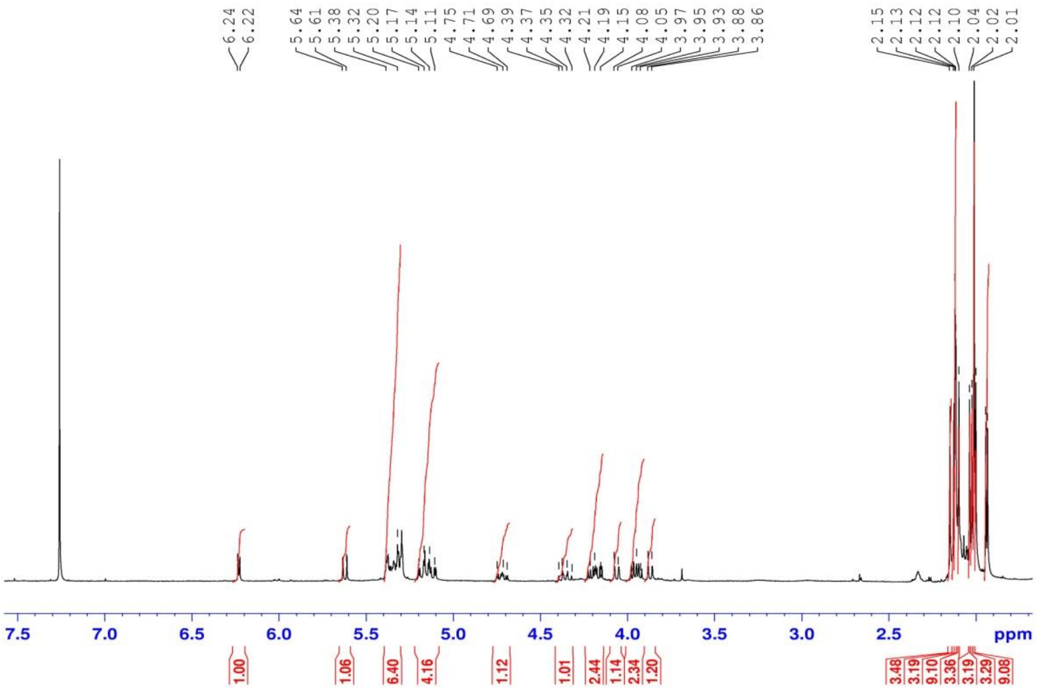
6.24
6.22
5.64
5.61
5.38
5.32
5.20
5.17
5.14
5.11
4.75
4.71
4.69
4.39
4.37
4.35
4.32
4.21
4.19
4.15
4.08
4.05
3.97
3.95
3.93
3.88
3.86
2.15
2.13
2.12
2.12
2.10
2.04
2.02
2.01
7.5
7.0
6.5
6.0
5.5
5.0
4.5
4.0
3.5
3.0
2.5
ppm
1.00
1.06
6.40
4.16
1.12
1.01
2.44
1.14
2.34
1.20
3.48
3.19
9.10
3.36
3.19
3.29
9.08

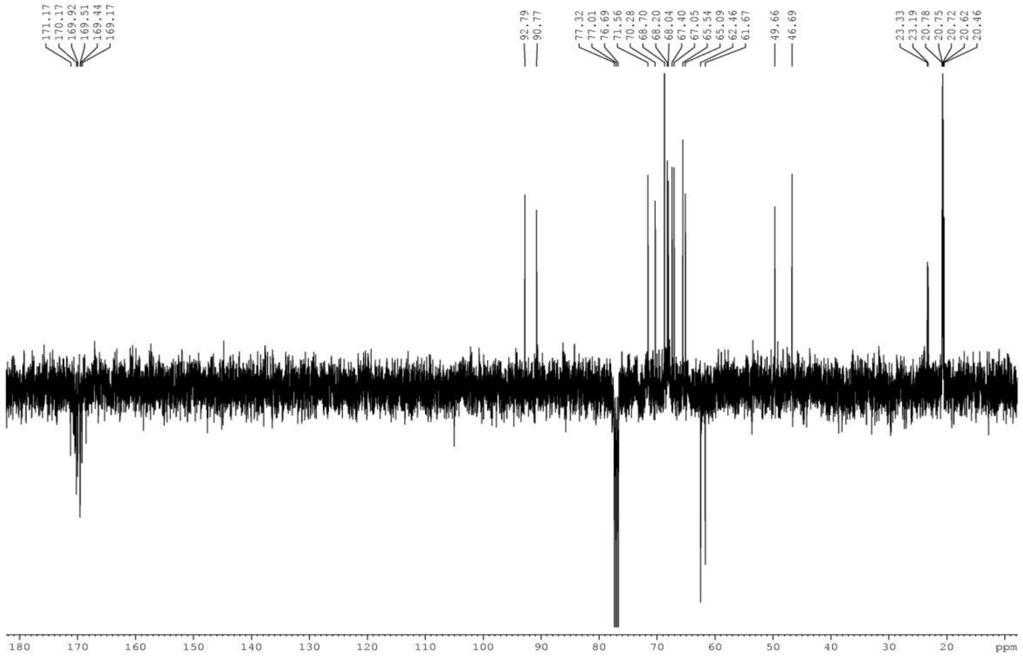
171.17
170.17
169.92
169.51
169.44
169.17
92.79
90.77
77.32
77.01
76.69
71.56
70.28
68.70
68.20
68.04
67.40
67.05
65.54
65.09
62.46
61.67
49.66
46.69
23.33
23.19
20.78
20.75
20.72
20.62
20.46
180
170
160
150
140
130
120
110
100
90
80
70
60
50
40
30
20
ppm

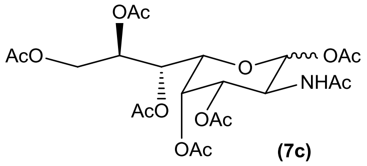

(7c)

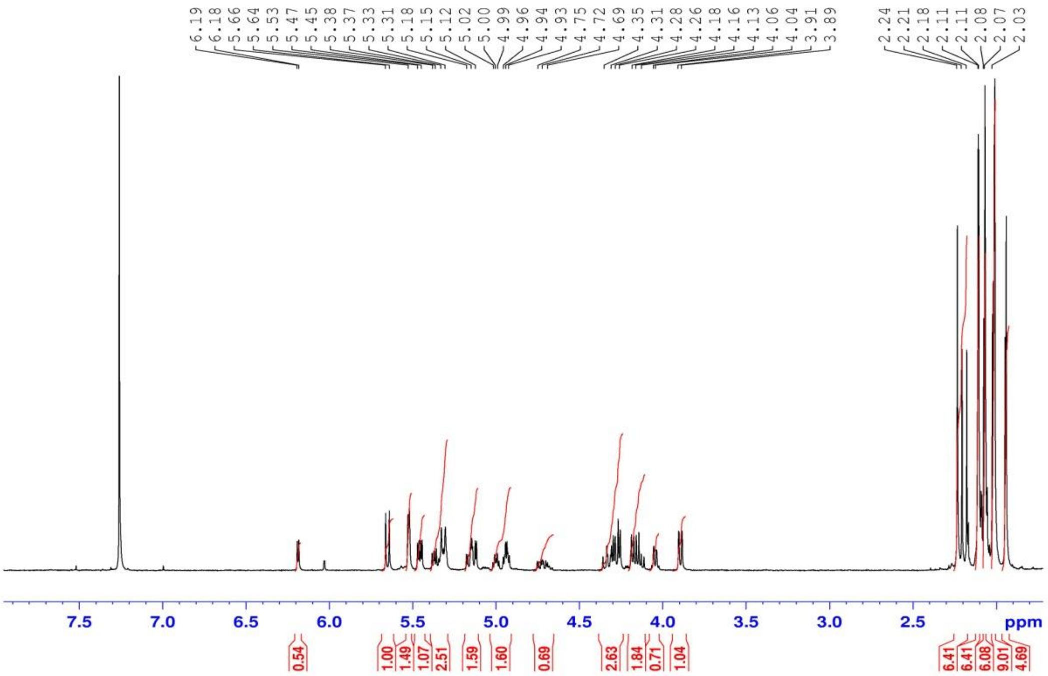

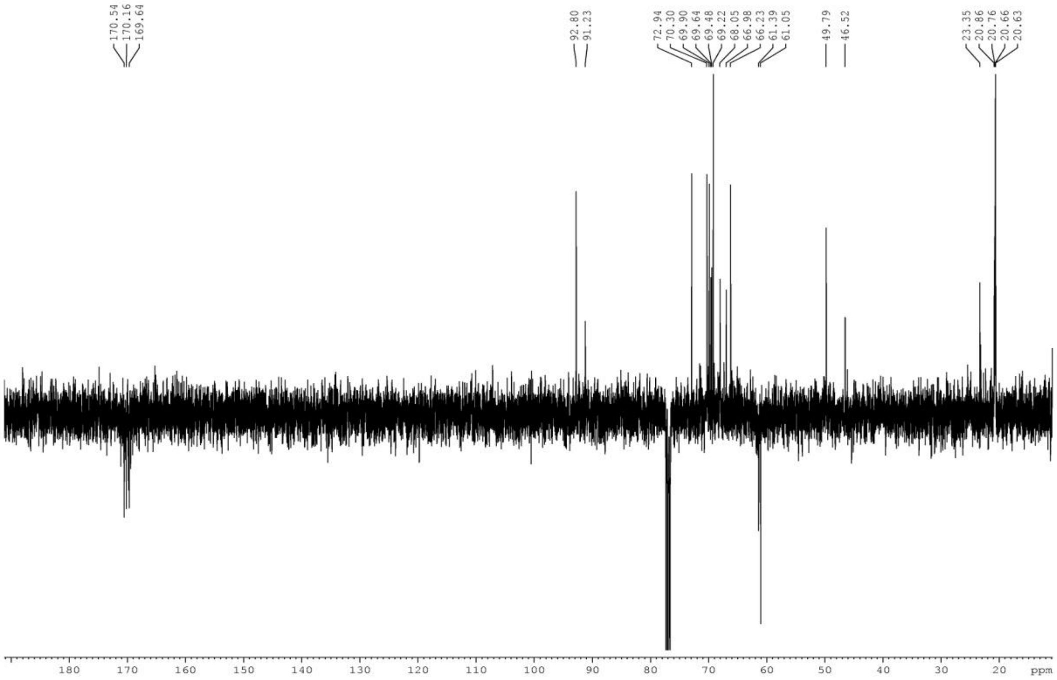

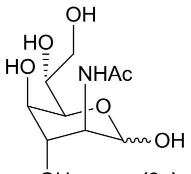
OH
HO
HO
NHAc
O
OH
OH

(8a)

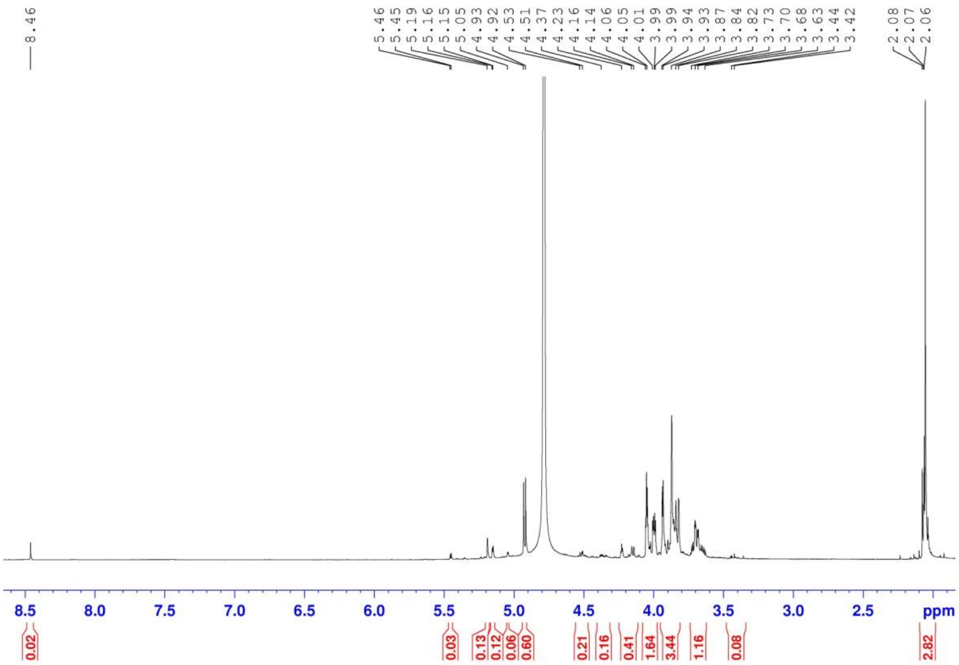
8.46
5.46 5.45 5.19 5.16 5.15 5.05 4.93 4.92 4.53 4.51 4.37 4.23 4.16 4.14 4.06 4.05 4.01 3.99 3.99 3.94 3.93 3.87 3.84 3.82 3.73 3.70 3.68 3.63 3.44 3.42
2.08 2.07 2.06
8.5 8.0 7.5 7.0 6.5 6.0 5.5 5.0 4.5 4.0 3.5 3.0 2.5 ppm
0.02 0.03 0.13 0.12 0.06 0.60 0.21 0.16 0.41 1.64 3.44 1.16 0.08 2.82

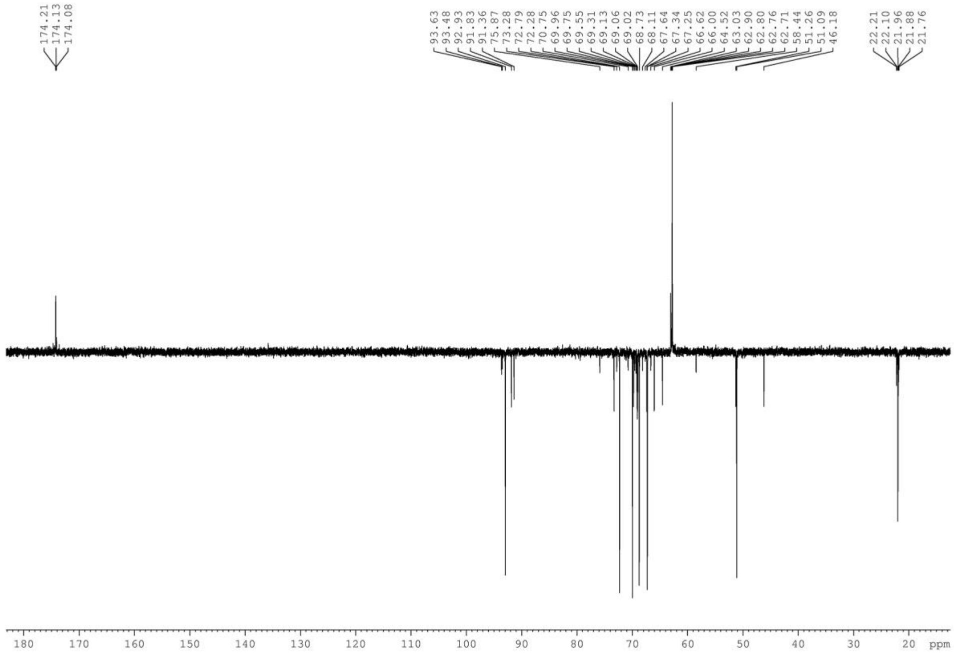
174.21 174.13 174.08
93.63 93.48 92.93 91.83 91.36 75.87 73.28 72.79 72.28 70.75 69.96 69.75 69.55 69.31 69.13 69.06 69.02 68.73 68.11 67.64 67.34 67.25 66.62 66.00 64.52 63.03 62.90 62.80 62.76 62.71 58.44 51.26 51.09 46.18
22.21 22.10 21.96 21.88 21.76
180 170 160 150 140 130 120 110 100 90 80 70 60 50 40 30 20 ppm

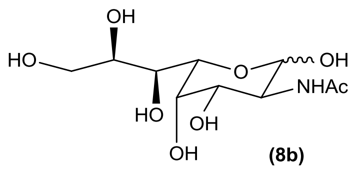
OH
HO
O
OH
NHAc
HO
OH
OH

(8b)

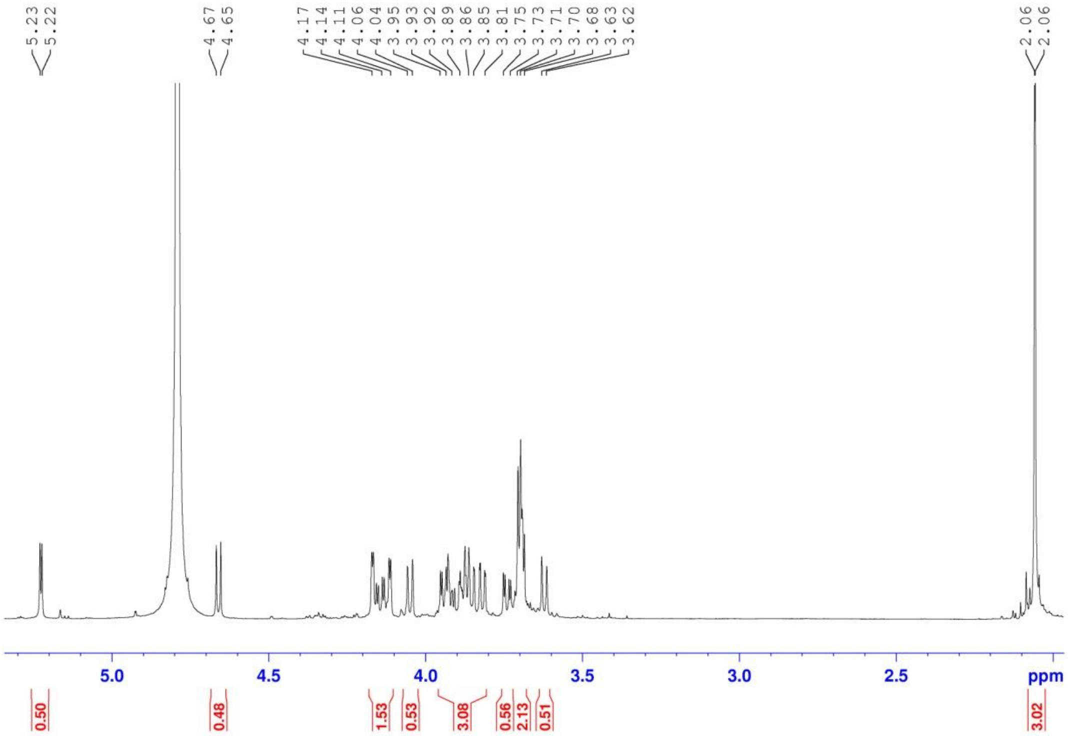
5.23
5.22
4.67
4.65
4.17
4.14
4.11
4.06
4.04
3.95
3.93
3.92
3.89
3.86
3.85
3.81
3.75
3.73
3.71
3.70
3.68
3.63
3.62
2.06
2.06
5.0
4.5
4.0
3.5
3.0
2.5
ppm
0.50
0.48
1.53
0.53
3.08
0.56
2.13
0.51
3.02

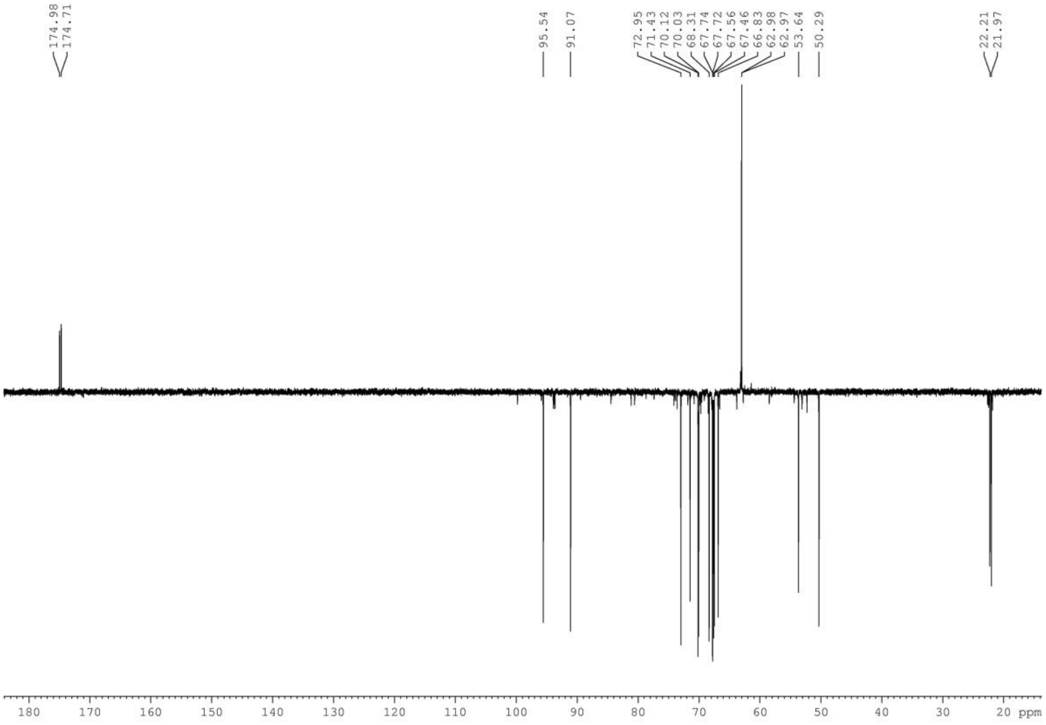
174.98
174.71
95.54
91.07
72.95
71.43
70.12
70.03
68.31
67.74
67.72
67.56
67.46
66.83
62.98
62.97
53.64
50.29
22.21
21.97
180
170
160
150
140
130
120
110
100
90
80
70
60
50
40
30
20 ppm

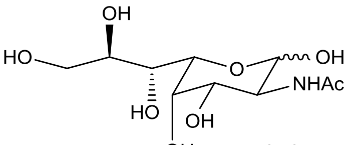
OH
HO
O
OH
NHAc
HO
OH
OH

(8c)

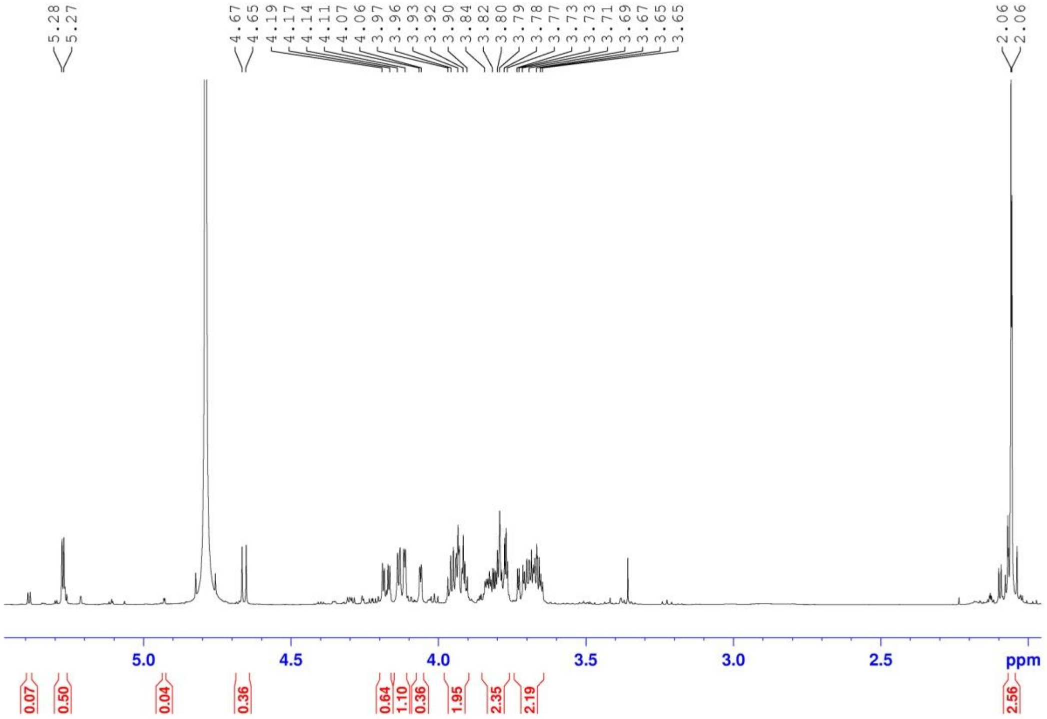
5.28
5.27
4.67
4.65
4.19
4.17
4.14
4.11
4.07
4.06
3.97
3.96
3.93
3.92
3.90
3.84
3.82
3.80
3.79
3.78
3.77
3.73
3.73
3.71
3.69
3.67
3.65
3.65
2.06
2.06
5.0
4.5
4.0
3.5
3.0
2.5
ppm
0.07
0.50
0.04
0.36
0.64
1.10
0.36
1.95
2.35
2.19
2.56

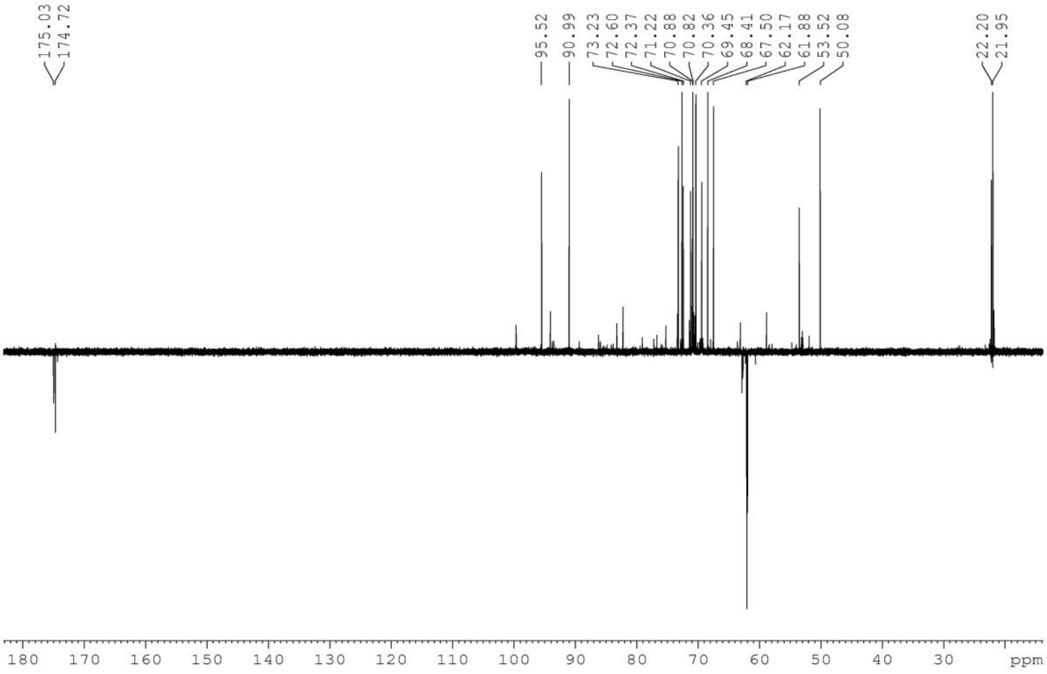
175.03
174.72
95.52
90.99
73.23
72.60
72.37
71.22
70.88
70.82
70.36
69.45
68.41
67.50
62.17
61.88
53.52
50.08
22.20
21.95
180
170
160
150
140
130
120
110
100
90
80
70
60
50
40
30
ppm
